# Evaluating the impact of COVID-19 on the HIV care continuum across global income levels: a mixed-methods systematic review

**DOI:** 10.1186/s12981-025-00778-w

**Published:** 2025-10-28

**Authors:** Emmanuela Ojukwu, Ava Pashaei, Juliana Cunha Maia, Oserekpamen Favour Omobhude, Abdulaziz Tawfik, Yvonne Nguyen

**Affiliations:** 1 School of Nursing, University of British Columbia, Vancouver, BC Canada; 2 Federal University of Ceará, Fortaleza, Brazil; 3 New York Medical College, New York, NY USA

**Keywords:** HIV care continuum, COVID-19, PrEP, Telemedicine, Income level, Facilitators, Barriers, Systematic review

## Abstract

**Background:**

The COVID-19 pandemic caused significant disruptions to global healthcare systems, including essential services along the HIV care continuum (HCC). While several studies have examined these impacts in specific countries or populations, limited evidence exists on cross-country differences in service disruptions, barriers, and facilitators stratified by national income levels.

**Methods:**

We conducted a mixed-methods systematic review following the Joanna Briggs Institute methodology and PRISMA 2020 guidelines. We searched CINAHL, MEDLINE, Embase, and CAB Direct for quantitative and qualitative studies published between March 2020 and January 2024. Eligible studies assessed the pandemic’s impact on one or more stages of the HIV care continuum, including prevention, testing, linkage to care, treatment engagement, antiretroviral therapy (ART) adherence, and viral suppression. Data were extracted, appraised, and synthesized using a convergent integrated approach across low-, middle-, and high-income countries as defined by the World Bank.

**Results:**

A total of 200 studies were included. The most frequently disrupted services were HIV testing, prevention (including pre-exposure prophylaxis [PrEP] use), and medical appointments, particularly in high- and middle-income countries. ART adherence and viral suppression showed greater resilience across all settings. Structural barriers, such as lockdowns, healthcare repurposing, and transportation limitations, were widespread, while digital exclusion, stigma, and socioeconomic inequities disproportionately affected marginalized populations. Key facilitators included telemedicine, multi-month dispensing of ART and PrEP, community-based service delivery, and national-level adaptations. The extent of disruption and success of mitigation strategies varied by income level, reflecting differences in health system preparedness and flexibility.

**Conclusions:**

The COVID-19 pandemic disrupted HIV care globally, with variation across income levels and care continuum stages. Health system resilience, equity in access, and pre-existing adaptive infrastructure significantly shaped outcomes. Findings highlight the need to institutionalize flexible, decentralized, and equity-informed service models to strengthen routine HIV care and pandemic preparedness.

**Supplementary Information:**

The online version contains supplementary material available at 10.1186/s12981-025-00778-w.

## Introduction

Throughout the COVID-19 crisis, healthcare delivery and public health services encountered significant disruptions globally [1]. The profound impact of the pandemic has strained healthcare systems, prompting the implementation of stringent lockdown measures to contain viral transmission [2, 3]. Consequently, access to healthcare services has been notably diminished, with healthcare facilities compelled to limit the provision of chronic disease management and treatment services [4, 5].

The impact of COVID-19 varies across countries, particularly concerning income levels, with challenges ranging from job loss to food insecurity and managing pre-existing health conditions. Adhering to preventive measures like social distancing and using personal protective equipment poses additional obstacles. Vulnerable groups, including people living with HIV (PLWH), individuals at heightened risk of HIV acquisition, such as those engaged in sex work, people who use drugs, men who have sex with men, and individuals with autoimmune disorders, face elevated health risks. Ensuring HIV prevention and care during global crises like COVID-19 is essential to addressing this public health challenge. This effort requires a strong focus on the full HIV Care Continuum (HCC), which includes diagnosis, linkage to care, retention in care, adherence to antiretroviral therapy, and viral suppression [6–8].

Many studies have reported the negative impact of the COVID-19 pandemic on HIV care access and delivery across different regions, particularly among PLWH [9–12]. However, few studies have taken a global perspective on how COVID-19 affected the entire HIV Care Continuum (HCC), especially through cross-country comparisons. To address this gap, we conducted a mixed-methods systematic review to synthesize both quantitative and qualitative evidence. This review aims to examine how the COVID-19 pandemic affected each stage of the HIV Care Continuum globally, including HIV prevention and pre-exposure prophylaxis (PrEP) use, testing and diagnosis, linkage to and receipt of care, HIV medical appointments, antiretroviral therapy (ART) adherence, treatment engagement, and viral suppression, and to explore how these impacts varied by country income level. The review further investigates key barriers and facilitators influencing HIV care delivery and outcomes across diverse health system and socioeconomic contexts.

## Materials and methods

### Study design and setting

This research constitutes a systematic review conducted in accordance with the Joanna Briggs Institute (JBI) methodology for mixed-methods systematic reviews (MMSR), which guided the design, data collection, and synthesis of quantitative and qualitative evidence. The review was initially undertaken in June 2022 and updated in January 2024. It was registered with PROSPERO (CRD42021285677), and a review protocol was developed to guide the process [13]. To ensure transparency and completeness in reporting, the study also adhered to the Preferred Reporting Items for Systematic Reviews and Meta-Analyses (PRISMA) 2020 guidelines.

This study is part of an extensive project examining the repercussions of the COVID-19 pandemic [13], including associated barriers and facilitators, across distinct country groups categorized by income levels, namely, low, middle, and high-income according to the World Bank Group classifications in the July 2022 [14]. Specifically, the current investigation compares findings in low, middle, and high-income countries. Comprehensive results for low-income countries [15], middle-income countries [16], and high-income countries [17] have already been compiled and are readily accessible.

### Data sources

Our search strategy was developed in consultation with a subject expert librarian at the University of British Columbia to locate both published and unpublished studies. Multiple variations and synonyms of the index terms “HIV” and “COVID-19” were searched in CINAHL (EBSCOhost), MEDLINE (Ovid), CAB Direct, and Embase (OVID). Using the COVID-19 special filter in OVID-Embase, a refined search approach was used to comb through databases including CINAHL, OVID-Medline, CAB Direct, and OVID-Embase. The COVID-19 special filter was used in Embase (Ovid), and a sample search strategy is presented in Appendix I. In addition, references in articles selected for full-text review were manually reviewed to identify further citations that match the criteria of this review.

### Study selection

The study selection process began with an initial search in the CINAHL database using the keywords “HIV” and “COVID-19.” Additional relevant terms were identified through the titles and abstracts of retrieved articles, and reference lists of included studies were manually reviewed to capture any additional eligible papers. The search covered literature published between March 2020 and January 2024.

All search results were uploaded into Covidence, where duplicate records were automatically removed. Two reviewers independently screened titles and abstracts to assess relevance. Full-text articles were then reviewed by the same two reviewers to determine eligibility, with reasons for exclusion documented within Covidence. Any disagreements during the selection process were resolved through discussion or, when needed, consultation with a third reviewer. The selection process was documented using a PRISMA flowchart to ensure transparency and reproducibility.

### Eligibility criteria

Studies were eligible for inclusion if they:

- Focused on the impact of the COVID-19 pandemic on the HIV Care Continuum (HCC), including prevention, diagnosis, treatment, or viral suppression;
- Included populations living with HIV or at risk of HIV acquisition;
- Employed observational, cross-sectional, qualitative, or mixed-method designs;
- Were published between March 2020 and January 2024;
- Were available in English or translatable with adequate accuracy.

Studies were excluded if they:

- Were duplicates or conference abstracts;
- Had no full text available;
- Were considered gray literature;
- Did not address the HCC;
- Were intervention studies, as the purpose of this review was to explore the pandemic’s real-world impact on HIV care access and delivery across settings, rather than evaluate the effectiveness of specific interventions.

### Data extraction

Both quantitative and qualitative data were extracted from studies included in the review by 2 independent reviewers using a self-developed extraction tool. When necessary, the data extraction tool was modified to accommodate the differences of each included study, and modifications were detailed in the systematic review. The data extracted were included specific details about the populations; study methods; theoretical framework, where applicable; phase of HCC; context; and outcomes of relevance to the review question, including the implications for clinical practice. Specifically, quantitative data were composed of outcomes of descriptive and inferential statistical tests. In addition, qualitative data were composed of verbatim themes or subthemes with corresponding illustrations and will be assigned a level of credibility. Any disagreements that arose between the reviewers were resolved through discussion or with an additional third reviewer. Where necessary, authors of papers were contacted to request missing or additional data.

#### Data synthesis and integration

This review employed a convergent integrated approach in accordance with JBI methodology for MMSR. Quantitative and qualitative data were initially extracted and analyzed independently. Thematic analysis was conducted on qualitative data to identify salient concepts, which were subsequently organized into descriptive themes. Quantitative findings that aligned with the review objectives were synthesized narratively. Where appropriate, quantitative data were qualitied to enable methodological integration.

Data synthesis was structured across seven predefined domains of the HIV care continuum: 1. HIV prevention and PrEP use (use of strategies, especially PrEP, to prevent HIV acquisition among at-risk populations), 2. HIV testing services (detection of HIV infection through provider-initiated, voluntary, or self-testing methods), 3. linkage and receipt of care (Initial connection to HIV care services after diagnosis, including ART initiation), 4. HIV medical appointments (ongoing clinic visits for HIV care, treatment monitoring, and health assessments), 5. HIV treatment engagement (long-term retention in HIV care and sustained use of ART services), 6. viral suppression (Reduction of HIV viral load to undetectable or low levels due to effective ART), and 7. retention in ART (consistent use of antiretroviral therapy as prescribed to maintain treatment effectiveness). Included studies were stratified by country income level, based on the World Bank classification system (low-, middle-, and high-income), to enable both within-group and cross-group comparisons.

During the integration phase, qualitative findings from mixed-methods and quantitatively oriented studies were transformed into narrative summaries and mapped to the relevant thematic domains. This process facilitated the synthesis of heterogeneous data sources into integrated line-of-action statements, which reflected areas of thematic convergence, divergence, and contextual variation. These statements emphasized the implications of COVID-19-related disruptions and adaptations across the continuum of HIV care.

All stages of data management and synthesis were conducted using Covidence, following the structured procedures outlined in the JBI MMSR framework to ensure transparency, methodological rigor, and reproducibility.

### Quality assessment

Given the inclusion of diverse study designs, multiple critical appraisal tools from the JBI were employed to assess methodological quality [18]. Qualitative studies were evaluated using the JBI Critical Appraisal Checklist for Qualitative Research, while quantitative studies were assessed using the corresponding JBI checklists appropriate to their design, including tools for analytical cross-sectional studies, cohort studies, and case series.

Quality assessment was conducted independently by two reviewers. Discrepancies were resolved through discussion or, when necessary, with the involvement of a third reviewer. Studies that did not meet the minimum quality criteria were excluded from the synthesis. Each study was classified according to its overall risk of bias: Low Risk of Bias, Medium Risk of Bias, or High Risk of Bias, based on the number of criteria met.

## Results

**Fig. 1:**
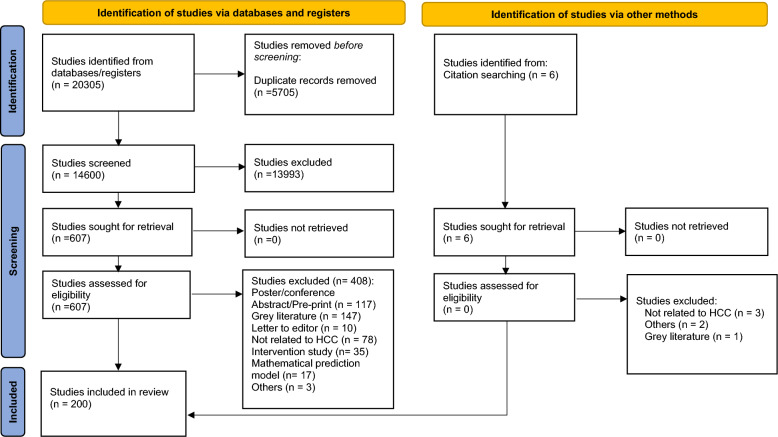
PRISMA 2020 flow diagram. *LIC Low-income countries, *MIC* Middle-income countries, *HIC* High-income countries

A total of 200 studies were included in the review. Of these, 143 were quantitative, 43 were qualitative, and 14 used mixed methods. The quantitative studies employed a variety of designs including cross-sectional, cohort, retrospective, longitudinal, and quasi-experimental approaches. The qualitative studies primarily involved qualitative descriptive and descriptive phenomenology. The mixed-methods studies combined quantitative and qualitative data collection and analysis, often using cross-sectional and descriptive formats. We employed a PRISMA diagram (Fig. 1) to illustrate our selection process. Each article underwent quality assessment, with all scoring 65% or higher, indicating moderate to high methodological quality (87% have high quality and low risk of bias). For a concise overview of our findings, please refer to Supplementary material Table 1.

Overall, the COVID‑19 pandemic had a predominantly negative impact on HIV services across all income settings and continuum stages, with testing, prevention and ART adherence studies most frequently reporting disruptions, especially in middle‑ and high‑income contexts. Positive outcomes were rare (most evident in viral suppression studies), and neutral findings made up only a small proportion of reports.

### HIV prevention and PrEP use

Prevention and PrEP use (PP) emerged as one of the most affected domains across the HIV care continuum during the COVID-19 pandemic. Among included studies, 38.6% in high-income and 21.8% in middle-income countries reported negative impacts on this domain. In contrast, disruptions were less frequently reported in low-income and multi-country studies, a trend that may reflect the limited baseline availability of PrEP services in these contexts. Positive impacts were documented only in high- and middle-income settings, where the implementation of adaptive service models, including remote and decentralized service delivery, may have supported continued access.

In low-income countries, only one study reported on HIV prevention and PrEP use [19]. This study documented a complete interruption in condom supply during the pandemic. Although quantitative data on PrEP uptake were not available, qualitative findings indicated reduced availability of condoms, family planning, and antenatal services, primarily due to funding constraints and limited clinic operations. Key barriers included lockdowns and fear of contracting COVID-19, while national PrEP expansion efforts helped mitigate service disruptions in some isolated areas.

Middle-income countries demonstrated more variability, with both declines and increases in PrEP and condom use. For example, 7% discontinued PrEP during lockdown, 6.6% reported reduced condom use, and 48% were unaware of PrEP [20]. In contrast, Brazil reported a 41.3% increase in PrEP dispensation from 2019 to 2020, with a further 22% increase in 2021[21]. Service disruptions were attributed to clinic closures, resource constraints, and behavioral barriers such as fear of exposure and stigma. However, multi-month dispensing, HIV self-testing, and telemedicine platforms emerged as consistent facilitators across contexts, supported by national PrEP programs and community-based interventions [22, 23].

In high-income countries, disruptions to PrEP services were substantial, with up to 41.8% discontinuation [24], and 9–25% reporting difficulty accessing prescriptions [25]. Barriers included fear of COVID-19, repurposing of services, logistical challenges, and disparities among marginalized populations. Despite this, condom access remained stable (89.4%), and many adopted on-demand PrEP or reduced sexual risk behaviors. Facilitators included telemedicine, at-home testing, and sustained insurance coverage, which helped mitigate the impact of service interruptions [26].

Multi-country studies revealed considerable heterogeneity in pandemic-related disruptions and adaptations. Quantitative data indicated that 56% reported PrEP interruptions and 10% reduced condom access [27]. Despite these challenges, PrEP uptake increased significantly in PEPFAR-supported countries, 157.2% across 21 countries, with a 214% rise in the PrEP-to-Need Ratio [28]. Common barriers included lockdowns, health worker reallocation, and closure of community channels. Facilitators consistently reported included telemedicine and multi-month dispensing, particularly among vulnerable populations such as transgender persons and youth.

### HIV testing services

HIV testing services (HTS) represented one of the most consistently disrupted components of the HIV care continuum during the COVID-19 pandemic. Negative impacts were reported in 38.6% of studies from high-income countries, 35.7% of multi-country studies, 34.6% of middle-income countries, and 28.6% of low-income countries. Only a small proportion of studies identified positive or neutral outcomes, underscoring the widespread nature of service disruptions. Although modest recoveries in testing volume were documented in some settings following the relaxation of public health restrictions, the overall evidence indicates that HIV testing was highly vulnerable to pandemic-related shocks across all income levels.

HIV testing services experienced sharp declines across LICs. Quantitative data showed a 39% reduction in overall testing uptake during lockdown [29], with 37.9% and 32.0% declines in VCT and PICT attendance, respectively [30]. Qualitative data echoed widespread disruptions [31]. Common barriers, also noted across other HIV care domains, included fear of infection, healthcare facility avoidance, travel restrictions, and eroding trust in health systems. Facilitators included mobile clinics, HIV self-testing, national guidelines, and PEPFAR support.

Middle-income countries exhibited heterogeneous outcomes. In some contexts, testing volumes declined sharply. In Brazil, testing decreased by 20.4% from 2019 to 2020, while the distribution of self-testing kits rose by over 200% [21]. Other studies identified over 50% reductions in any form of testing and challenges accessing both HIV self-tests and facility-based diagnostics [23, 32, 33]. Barriers included lockdowns, economic strain, mental health burdens, and reduced clinic access, alongside decreased demand due to reduced risk behavior. Facilitators such as telemedicine, e-commerce HIV self-testing, and community-based services helped sustain testing. Civil society and digital innovations were vital in maintaining access.

In high-income countries, HIV testing services faced moderate but widespread disruptions. Testing declines ranged from 26.9% to 60%, especially in outpatient and community-based settings [34–37]. Emergency department testing remained relatively stable or increased [24, 38] or, in some cases, increased [39–41]. Barriers included reduced hours, staffing shortages, and structural disparities, particularly affecting marginalized groups. Key facilitators were telehealth, self-testing, and opt-out testing models, with innovations like outdoor sites and virtual consultations supporting access.

Multi-country studies highlighted both the breadth of disruptions and the diversity of mitigation efforts. Quantitative analyses revealed a reduction ranging from 26.2% to 44.6% in testing volume, with Latin America and the Caribbean exhibiting some of the steepest regional declines [42]. Over half of surveyed sites across five countries reported suspension of community-based testing services [43]. Barriers and facilitators largely mirrored other settings, with telemedicine and mobile testing helping offset service disruptions. Equity gaps remained more prominent among girls, young women, and gender-diverse groups.

### HIV medical appointments

HIV medical appointments (HMA) were substantially disrupted during the COVID-19 pandemic, emerging as one of the most affected domains across all income settings. Negative impacts were reported in 28.6% of studies from low-income countries**,** 25.6% from middle-income countries, and 25% from high-income countries. While a greater proportion of neutral findings was observed in high-income contexts, likely reflecting the broader availability of telemedicine and remote consultation platforms, the overall continuity of care was compromised in all regions. The consistent disruption of scheduled visits, coupled with variable adaptation strategies, highlights the persistent vulnerabilities of appointment-based HIV care delivery during public health emergencies.

In low-income countries, disruptions to HIV medical appointments were consistently documented across both quantitative and qualitative data sources. Approximately 24–26% of individuals reported missed HIV care visits or counseling sessions [44, 45], and over half of those lacking access to community outreach experienced difficulties obtaining TB or HIV services [46]. Barriers such as lockdowns, transport issues, provider stigma, food insecurity, and fear of COVID-19 overlapped with other stages of the HIV care continuum. Older adults (≥ 55 years) were especially affected. Reduced wait times and service streamlining were key facilitators [47]. In middle-income countries, appointment disruptions were more heterogeneous but remained substantial. Quantitative studies reported missed appointment rates ranging from 20% to 36.4% [48–50], and nearly 57% experienced at least mild difficulty accessing services [51]. In certain contexts, the median number of clinic visits declined markedly, and one study observed a 48% reduction in hospital visits [52]. Barriers included staffing shortages, transportation issues, and limited access to telemedicine. Intimate partner violence and psychological distress also interfered with access. Facilitators included remote consultations, clinic flexibility, and virtual support platforms.

In high-income countries, despite relatively more robust healthcare infrastructure, considerable changes to service delivery and patient behavior were observed. Missed or cancelled appointments ranged from 6 to 45% [53–56], and approximately one-third of participants reported reduced clinic attendance [40, 57, 58]. However, some studies during the pandemic reported no difficulty for 55.7% of participants [38], no change in clinic visits [59], a 3% increase in visit completion [60], and a 37% rise in virtual visits [59]. Some reported dissatisfaction with telemedicine, disruptions to lab services, and privacy concerns [61–63]. Barriers included social determinants like food insecurity and mental health challenges. Facilitators included telehealth expansion, proactive outreach, and home-based care options.

Findings from multi-country studies highlighted significant regional variation, often shaped by local health system capacity and policy response. While no country reported complete HIV clinic closures, approximately 15% experienced reduced operating hours or postponed non-urgent appointments [43, 64]. Countries in Africa and Latin America experienced more pronounced reductions in in-person consultations (7.1% to 24.3%), while certain European and Asian settings reported modest increases, potentially reflecting stronger uptake of virtual care models [42]. Common barriers such as lockdowns and staffing shortages were counteracted by telemedicine, same-day ART initiation, and community-based ART distribution, which supported continuity of care.

### ART adherence

Across all income-level settings, adherence to ART (AA) experienced notable disruptions during the COVID-19 pandemic. The magnitude and nature of these disruptions varied by context, with structural, individual, and systemic factors shaping both barriers and facilitators to maintaining consistent ART use. Middle-income countries saw the highest rate of negative findings (51.3%), followed closely by low-income settings (52.4%). High-income countries presented a more mixed picture, with more studies reporting neutral or positive effects, likely due to stronger infrastructure and more reliable medication delivery systems. Despite this variation, ART adherence remained a vulnerable aspect of care globally, particularly in low- and middle-income countries, where adaptive mechanisms were often limited.

ART adherence was significantly impacted in LICs. Quantitative findings showed a 20.2% drop in ART clinic visits [30] and with 11% discontinuing ART [44], and up to 14% reporting COVID-19’s adverse effect on adherence [10]. Common barriers mirrored those in other stages, COVID-19 fears, travel issues, food insecurity, healthcare worker shortages, and stigma. Facilitators included multi-month dispensing, home delivery via village health teams, continued community-based services, and mental health support [65].

In middle-income countries, ART adherence outcomes were mixed, with both disruptions and adaptive responses documented. Quantitative data revealed that 41% of patients exhibited inadequate adherence [57], while between 14.5% and 50% reported challenges in medication access or consistency [21, 48, 50, 66–69]. ART interruptions ranged from mild to severe, with some studies reporting a 40% decrease in adherence [69] and a 94% drop in ART initiation among key populations like female sex workers [23]. Key barriers included fear of disclosure, stigma, and limited access to ART. Yet, multi-month dispensing, community ART delivery, and telemedicine facilitated ongoing adherence, supported by NGOs and peer networks.

In high-income countries, ART adherence was relatively more stable but still affected for certain populations. Quantitative studies reported that 13.2% of patients avoided ART pickup, and 8.2% missed at least one dose in the prior month [53]. While 72% of participants reported no difficulty accessing ART [70], disruptions were observed in specific subgroups, including people with lower income, racial and ethnic minorities, and individuals with mental health or substance use challenges [61, 71]. Disparities persisted for those with low income, mental health conditions, or housing insecurity. Facilitators included drive-thru pharmacies, mail delivery, extended refills, and provider outreach.

Multi-country studies reflected varied experiences across settings, with both high and low disruption rates observed. While 93.2% of surveyed participants were on ART, only 33.3% achieved viral suppression [72], and 18.9% reported difficulties accessing ART [27]. Barriers included structural inequities and stigma, particularly among marginalized populations. Facilitators such as telemedicine, web-based tools, and same-day ART contributed to program resilience.

### Linkage and receipt of care

Linkage to and receipt of HIV care (LRC) were moderately disrupted across all income settings during the COVID-19 pandemic, with the severity of impact varying by context. The highest proportions of negative outcomes were observed in low-income (38.1%) and middle-income (28.2%) countries, where service delivery interruptions and delayed treatment initiation were more frequently reported. In contrast, studies from high-income and multi-country contexts indicated fewer disruptions, with some documenting successful adaptations such as virtual consultations and modified service delivery protocols. The near absence of neutral findings suggests that most changes were directional, typically negative, rather than reflective of system stability. Despite evidence of resilience in certain settings, timely access to HIV care remained vulnerable, particularly for marginalized populations. These findings underscore the critical importance of flexible, equity-focused strategies to sustain HIV care continuity during public health emergencies.

In low-income countries, linkage to care was notably disrupted, with 76% reporting negative impacts on clinic travel and 54% perceiving increased COVID-19 exposure risk in clinical settings [10]. Reductions in ART initiation (5–19%) and care reengagement were driven by lockdowns, transportation barriers, livelihood loss, and provider stigma, particularly for those newly initiating ART [73–77]. Individuals who had recently initiated ART were among the most vulnerable to care discontinuation. Key barriers included COVID-19 lockdowns, restricted transportation, fear of COVID-19 exposure, and disruptions to social life and livelihoods. Structural limitations such as food insecurity, clinic access disruptions, and negative provider attitudes further compounded these challenges. Conversely, reported facilitators included individual-level characteristics such as having basic education, being married, and access to healthcare facilities.

In middle-income countries, findings showed heterogeneous patterns. Some sites maintained 100% linkage to care due to uninterrupted ART services [78], while others observed a substantial decline in ART initiation, up to 48% in some cases [21, 69, 79], and overall 56% reduction in HIV service uptake [80, 81]. Specific groups, such as female sex workers, experienced a 94% drop in ART initiation [82]. A few settings documented recovery phases, with 13–39% increases in ART initiation during later stages of the pandemic [21, 83]. Barriers included economic hardship, stigma, and service closures, especially for adolescents and sex workers. Facilitators included telemedicine, NGO support, flexible clinics, and targeted youth services.

In high-income countries, LRC disruptions were more moderate and often mitigated through virtual services. A study reported that 55% of participants were able to access their HIV provider during the pandemic [84], while 20.6% faced untimely access to ARTs [58]. New patient interactions declined by 23.5% in early 2020, and some racial and ethnic minority groups (e.g., Hispanics and Haitians) were more likely to experience care access challenges [85]. Barriers like technological gaps, privacy issues, and economic instability were common. Facilitators included video visits, proactive outreach, and multi-channel care access.

Multi-country studies showed varied experiences, highlighting disparities between countries with strong digital health systems and those facing persistent structural inequities. Quantitative data indicated that nearly 20% of participants were unable to access their provider, while only 14% succeeded via telemedicine innovations [27]. ART initiation declined during the strictest phases of the pandemic, with partial recovery noted later [86]. Lockdowns, economic strain, and service disruption hindered engagement, especially among minority groups. Facilitators such as community ART pickup and web-based support promoted continuity.

### HIV treatment engagement

HIV Treatment engagement (HTE) represented one of the least disrupted domains across the HIV care continuum during the COVID-19 pandemic. Quantitative data indicated that negative impacts were most frequently observed in middle-income countries (10.3%), while high-income settings reported a greater proportion of neutral outcomes, suggesting relative preservation of service continuity through adaptive strategies. Nevertheless, engagement in HIV care was substantially challenged across all country income levels. Individuals encountered interruptions in routine services, reduced access to clinical care, and a range of psychosocial and structural barriers that impeded sustained engagement. These challenges were particularly pronounced in low- and middle-income contexts, though high-income and multi-country studies also documented significant difficulties among vulnerable subpopulations. Despite variability in the magnitude of disruption, findings underscore the importance of resilient, patient-centered service models capable of maintaining care engagement during public health emergencies.

In low-income countries, treatment engagement was markedly disrupted. Quantitative findings indicated that 27.4% of individuals missed ART refill visits [45], and only 19.1% remained retained in care [87]. Barriers included lockdowns, clinic closures, financial hardship, fear of disclosure, and stigma, echoing challenges noted in adherence and linkage. Facilitators included mobile clinics, appointment reminders, and differentiated service delivery via national programs and PEPFAR support.

In middle-income countries, treatment engagement was affected by a range of systemic and individual-level stressors. Studies reported that up to 60.4% of PLWH were unable to receive regular follow-up services [88], and 37.2% reported difficulty accessing routine HIV care [48]. Contributing factors included transport disruption, limited psychosocial support, and adolescent service gaps. Facilitators included virtual counseling, multi-month dispensing, and peer support systems.

In high-income countries, treatment engagement outcomes were more varied. While healthcare systems were generally more adaptable [40, 89, 90], certain populations, such as people who use drugs [91], younger PLWH [92], and individuals facing unstable income or housing [93], encountered significant barriers. Lack of behavioral health coverage and mental health burden were prominent. Facilitators included telehealth, free transport, and expanded access models.

Multi-country studies underscored the pandemic’s disproportionate effect on treatment engagement among marginalized groups such as MSM**,** racial/ethnic minorities**,** immigrants**,** and sex workers [94]. Barriers included job loss and educational disparities. Facilitators were effective among older, educated individuals with socioeconomic stability.

### Viral suppression

Viral suppression (VS) was the least affected domain of the HIV care continuum during the COVID-19 pandemic. Across all income-level settings, suppression rates remained largely stable, with few studies reporting negative impacts. In some cases, particularly in high-income countries, positive or neutral findings were observed, likely due to stable ART adherence or delays in viral load monitoring. While reductions in laboratory access and testing frequency were noted, these did not appear to compromise overall viral suppression outcomes. These findings highlight viral suppression as the least disrupted phase of the continuum, demonstrating the resilience of ART programs and the success of differentiated service delivery models in maintaining viral control throughout a public health emergency.

In low-income countries, viral suppression rates either remained stable or slightly improved [75, 87], increasing from 93 to 94% in one study [95]. This occurred despite disrupted viral load testing, travel costs, PPE shortages, and clinic closures. Facilitators like multi-month dispensing, national service continuity guidelines, and ART access programs supported stability. Older adults, those on long-term ART, and women showed stronger viral control.

Viral suppression in middle-income countries generally remained stable. Most studies reported neutral outcomes, with no statistically significant changes observed [83, 96]. One study indicated an improvement in viral suppression during the pandemic compared to the pre-pandemic period, despite an increased proportion of individuals presenting with low CD4 counts (CD4 < 350 cells/mm^3^ in 36.4% and 47.9%, respectively) [97]. Facilitators included early ART and telemedicine, even where viral load testing declined.

In high-income countries, viral suppression rates were largely preserved [40, 59, 97]. Some sites even reported improvements, with suppression increasing from 98% to 99.2% during the pandemic [98]. Challenges included racial disparities and service repurposing. Facilitators included home delivery, virtual adherence counseling, and digital access tools.

Multi-country studies offered a heterogeneous picture. While a 21.9% reduction in viral load testing was reported in some contexts [99], there was no evidence of widespread suppression failure. Facilitators like multi-month dispensing and telemedicine were widely credited for success, especially in systems with existing infrastructure.

**Fig. 2:**
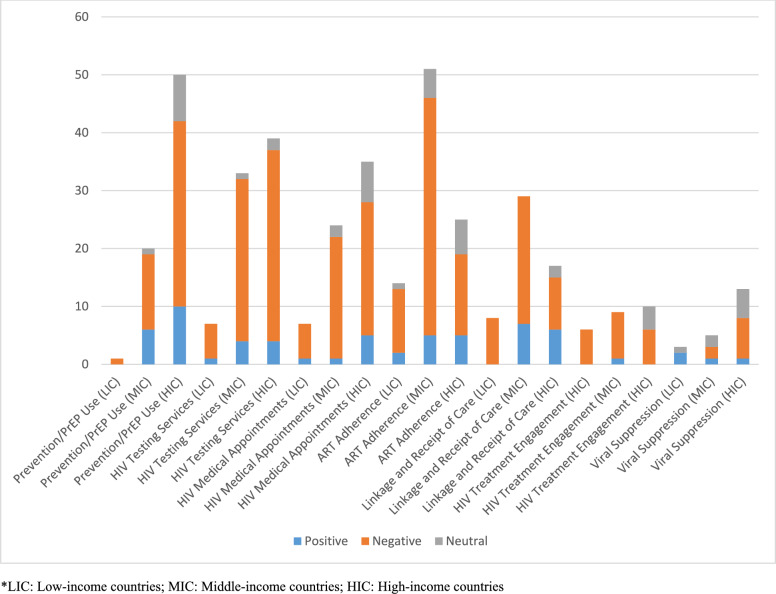
A comparative analysis of impact of COVID-19 pandemic on HCC across different income categories

The Fig. 2 presents a comparison of the impact of different stages in the HIV Care Continuum across countries categorized by income levels. The most impacted stage, ART adherence, holds the highest impact compared to other stages, this suggests that maintaining adherence to antiretroviral therapy has been challenging during the pandemic. Conversely, the lowest stages, viral suppression and HIV treatment engagement, exhibit the lowest impact across income categories. In high-income countries, the stage most significantly impacted is Prevention and PrEP use, whereas in middle- and low-income countries, it is ART adherence. Positive impacts frequently stem from telemedicine, a factor notably lacking in low-income countries. High-income countries demonstrate the most neutral impact compared to other countries. The overall impact depicted in the figure is negative, yet without further context or specific measures, it remains unclear which aspect of impact is referenced, such as health outcomes or program effectiveness. Moreover, the figure indicates that most studies conducted or available data focus on stages within middle- and high-income countries, highlighting a relatively higher level of research and attention given to these stages in countries with middle- and high-income levels.

**Fig. 3:**
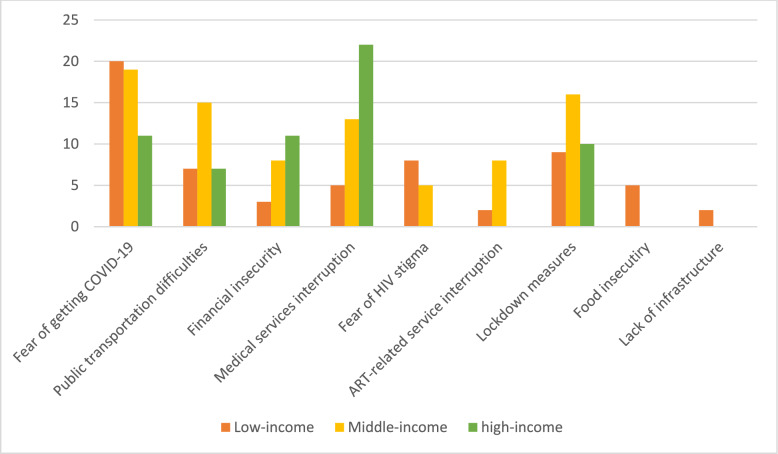
Comparison of the HCC barriers in high-income, middle-income, and low-income countries

*Barriers and Facilitators of HCC:* During the pandemic, fear of contracting COVID-19 emerged as the primary barrier to seeking HIV care across all countries. This fear was particularly pronounced in low-income countries (20/21) and middle-income countries (19/76) (see Fig. 3). In high-income countries, medical service interruptions took precedence as the leading barrier (22/88), highlighting concerns about healthcare accessibility amidst the crisis (see Fig. 3).

Additionally, lockdown measures, disruptions in public transportation, and financial insecurity were prevalent barriers across all income levels (see Fig. 3). Notably, food insecurity and lack of infrastructure emerged as unique challenges exclusively observed in low-income countries (see Fig. 3).

**Fig. 4:**
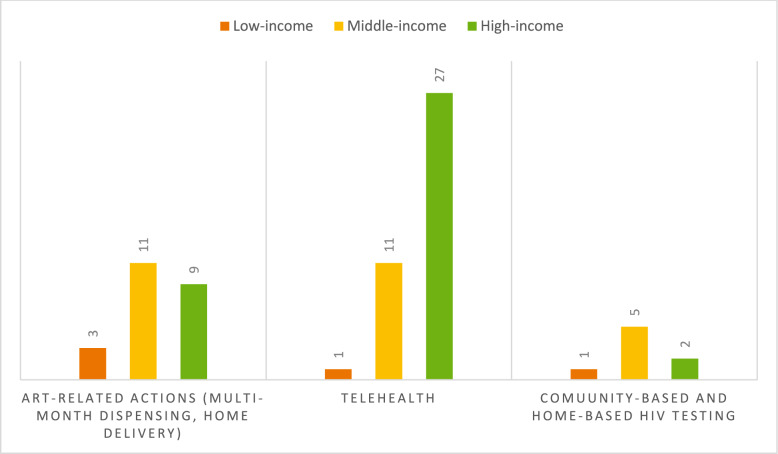
Comparison of the HCC facilitators in high-income, middle-income, and low-income countries

**Fig. 5:**
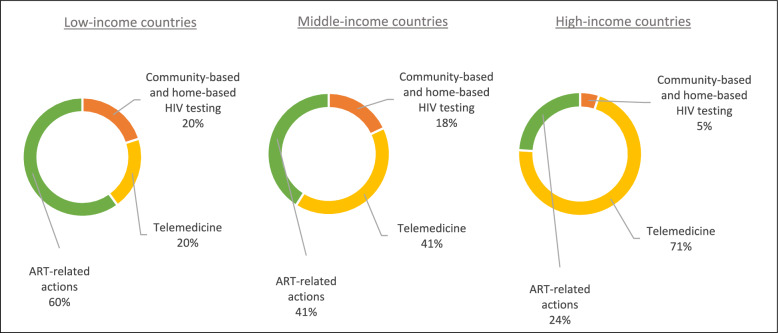
HCC facilitators distribution in low-income, middle-income, high-income countries

Telemedicine emerged as a pivotal component of HCC strategies during the pandemic, particularly prevalent in high-income countries where it was mentioned in 27 out of 88 instances (See Figs. 4**, **5). Following closely were ART-related initiatives such as multi-month dispensing and home delivery of ART, which played a significant role in ensuring uninterrupted access to medication. Additionally, community-based and home-based HIV testing initiatives proved instrumental in enabling PLWH and people at risk to continue undergoing HIV tests despite the challenges posed by lockdown measures. In low-income countries, as illustrated in Fig. 5, ART-related actions emerged as the primary facilitator. Similarly, in middle-income countries, ART-related measures, along with telemedicine, also took precedence as the leading facilitator.

## Discussion

### Income-level disparities

The COVID-19 pandemic revealed substantial disparities in the resilience and adaptability of HIV care systems across different country income levels. Although high-income countries benefited from more robust health infrastructures and digital capacities, critical disruptions were still observed, particularly in prevention and testing services. Middle-income countries exhibited heterogeneous responses, with outcomes shaped by the degree of coordination, resource availability, and policy implementation. Low-income countries experienced the most severe service interruptions, primarily due to limited infrastructure, constrained workforce capacity, and challenges in implementing alternative care models. The effectiveness of adaptive strategies, such as telemedicine, multi-month medication dispensing, and community-based service delivery, varied significantly across settings, reflecting differences in system preparedness and population reach. Additionally, intersecting structural and psychosocial vulnerabilities, including economic instability, stigma, and mental health burdens, further influenced service continuity.

### Structural capacity and health system responses

High-income countries, although better resourced and equipped, experienced service disruptions across multiple domains of HIV care, particularly in prevention and testing. Despite the availability of electronic medical records, telemedicine infrastructure, and a larger healthcare workforce, the reallocation of services to support the COVID-19 response resulted in delayed HIV appointments and the suspension of outreach programs. While these countries were comparatively better positioned to adapt service delivery, particularly through telemedicine and pharmacy-based models, their existing infrastructure did not uniformly prevent disruptions. This indicates that health system resilience depends not only on resource availability but also on the capacity to reorganize care pathways efficiently during public health emergencies.

In contrast, low-income countries were constrained by limited infrastructure, a less flexible workforce, and minimal contingency planning. The reliance on facility-based care, compounded by transportation barriers, staff shortages, and inadequate access to protective equipment, contributed to more prolonged service interruptions. Middle-income countries presented a more heterogeneous picture. Some settings, such as Brazil and South Africa, implemented timely adaptations, such as multi-month dispensing and community-based service delivery, while others struggled due to system fragmentation and limited coordination across public health sectors. Similarly, a systematic review reported that low- and middle-income countries experienced substantial disruptions in the provision of healthcare services [100]. A literature review also identified multi-month dispensing of antiretroviral therapy as a facilitating policy to prevent disruptions in antiretroviral supply and reduce individuals’ exposure to COVID-19 when accessing HIV services [101]. These cross-country comparisons highlight the importance of not only building robust health systems but also establishing agile, decentralized, and equity-focused service models capable of maintaining continuity during future systemic shocks.

### Intersectional inequities in service access

Pre-existing inequities significantly shaped access to HIV services during the COVID-19 pandemic. In low- and middle-income countries, rural populations, women, and individuals from economically marginalized groups experienced greater service disruptions due to geographic isolation, limited digital and transportation infrastructure, and persistent social barriers. In high-income countries, disparities were observed along racial, gender, and immigration lines. Transgender, non-binary, and racialized populations reported increased difficulty accessing services, including telemedicine, and in some cases were deprioritized for care.

Across all settings, the pandemic exacerbated structural inequalities. For instance, in LICs, adolescent girls and young women faced heightened challenges following the closure of safe spaces and reproductive health clinics. In high income countries, youth and LGBTQ + individuals frequently encountered privacy constraints when attempting to access virtual services from shared or unsupportive living environments. These patterns illustrate how intersecting factors, such as gender, age, socio-economic position, and geographic location contributed to varied experiences of service disruption. Addressing such disparities requires targeted, inclusive interventions that are responsive to the multiple and overlapping forms of marginalization encountered across diverse population.

### Adaptation strategies and system flexibility

Several interventions functioned as critical facilitators across different country contexts. In high-income countries, telemedicine (e.g., U.S. implementation of virtual clinics and remote prescription refills), home-based testing, and prescription delivery programs were rapidly implemented and expanded, contributing to the maintenance of care continuity. In middle-income countries, multi-month dispensing of ART and PrEP (e.g., Brazil’s utilization of community health agents for ART delivery), in conjunction with e-health initiatives and community-based ART distribution, were essential in mitigating service disruptions. However, the extent and consistency of implementation varied significantly depending on infrastructural readiness and governmental coordination. In low-income countries, adaptations such as medication delivery through village health teams and the use of simplified ART regimens (e.g., South Africa’s national rollout of 3–6 month ART dispensing during COVID-19) showed promise, although these efforts were frequently constrained by limited scale and inconsistencies in supply chains. A review examining the role of telemedicine in HIV care delivery emphasized its effectiveness in maintaining service continuity. However, the study also identified significant disparities in telemedicine accessibility across countries with varying income levels, which may contribute to differential health outcomes [102].

In several low- and middle-income country contexts, community-based strategies played a critical role in maintaining HIV care delivery. These included the deployment of community health workers, community-based distribution of ART and PrEP, and engagement of community-based organizations (CBOs) to support home-based care. Such initiatives were particularly valuable in reaching underserved populations and ensuring continuity of services during lockdowns. Future emergency response planning should incorporate these equity-oriented, community-led models more systematically to enhance resilience and reach in public health crises.

The most effective adaptations were those integrated into pre-existing health system structures or supported by policies established prior to the pandemic, including national PrEP initiatives and differentiated service delivery models. Institutionalizing such equity-oriented and community-led approaches within national HIV emergency frameworks could enhance system resilience and responsiveness in future public health crises. These observations suggest the importance of embedding flexible, decentralized strategies into routine HIV care delivery as a standard approach, rather than as reactive measures during periods of crisis.

### Service domain-specific impacts

The COVID-19 pandemic did not affect all components of the HIV care continuum uniformly. Service domains that required regular, in-person interactions, such as HIV testing and prevention, including PrEP, were particularly susceptible to disruption. Their dependence on facility-based service delivery, in-person outreach, and ongoing clinical engagement increased vulnerability to lockdown measures and the reallocation of resources toward COVID-19 responses. In contrast, domains such as ART adherence and viral suppression demonstrated greater stability, particularly in settings where multi-month dispensing and community-based medication distribution were already established. Supporting this observation, a global review highlighted the expansion of multi-month dispensing and alternative ART delivery mechanisms during the pandemic. In sub-Saharan Africa, multi-month dispensing rates rose substantially, and various delivery approaches, including home-based delivery, pharmacy refills without new provider orders, and ART distribution via community centers or mail, were adopted to reduce clinic visits and sustain treatment access [103].

Notably, viral suppression remained largely stable despite reductions in viral load monitoring. This observation suggests that sustained access to treatment, rather than frequent laboratory testing, was more influential in maintaining viral control. These findings support the effectiveness of simplified and decentralized care delivery models, which are less reliant on frequent patient-provider contact and more adaptable during public health emergencies. This is further supported by evidence that, although face-to-face provider engagement facilitates client linkage to HIV services, such interactions were often unfeasible during the pandemic, yet viral control was maintained through alternative models of care [104].

### ‌Behavioral and psychological factors

In addition to structural and system-level challenges, behavioral and psychosocial factors played a critical role in shaping engagement with HIV care during the COVID-19 pandemic. Fear of contracting COVID-19 was a common deterrent to care-seeking behavior across all country income levels. In low- and middle-income countries, this fear was further compounded by stigma, misinformation, and financial instability. In high-income countries, individuals frequently reported concerns related to privacy, dissatisfaction with telemedicine services, and heightened anxiety as contributing factors to disengagement from care. These patterns are consistent with prior evidence suggesting that pandemic-related disruptions, social isolation, and heightened psychological stress exacerbated mental health issues among people living with HIV, potentially compromising treatment adherence and overall HIV management [105]. Consistently, a literature review emphasized the value of multidisciplinary approaches in supporting the mental health of people living with HIV [101].

Behavioral changes, such as reductions in sexual activity or increased monogamy, were associated with decreased demand for HIV testing and PrEP use. Concurrently, social isolation and economic pressures contributed to the worsening of mental health conditions, which negatively affected medication adherence and attendance at medical appointments. Integrated mental health services and peer support interventions were used to address psychological stress and stigma and to support continued engagement in HIV care during crises. Integrating mental health services, peer support mechanisms, and adaptable service delivery options into HIV care programs was used as a strategy to maintain engagement during public health emergencies.

## Policy implications and future readiness

The COVID-19 pandemic revealed both the strengths and weaknesses of global HIV care systems, underscoring the importance of differentiated and equity-oriented approaches tailored to specific contexts. For low-income countries, future preparedness should emphasize the scale-up of community-based care models, decentralized antiretroviral distribution systems, and mobile outreach services. While expanding telemedicine remains a long-term objective, its success depends on concurrent investments in infrastructure, digital literacy, and culturally appropriate service provision.

Middle-income countries require sustained political commitment to implementing differentiated service delivery models and reinforcing subnational health systems. Promising interventions, such as government-led PrEP programs, e-health infrastructure development, and public–private collaborations, should be further institutionalized and scaled.

In high-income countries, health system resilience must extend beyond technological readiness to address persistent structural inequities. Ensuring continuity of care during emergencies necessitates policies that protect access for marginalized populations, including reforms in insurance coverage, anti-stigma measures, and equitable digital access strategies.

At the global level, pandemic preparedness efforts must integrate the ongoing management of chronic conditions such as HIV. HIV care should be embedded within broader emergency response frameworks, supported by predefined protocols for service continuity and multisectoral coordination. Achieving system resilience requires designing healthcare infrastructure that is not only efficient but also inclusive and responsive to the needs of all populations, particularly during times of crisis.

## Conclusion

The COVID-19 pandemic highlighted critical policy deficiencies affecting the continuity of HIV care across country income levels. In high-income settings, digital infrastructure facilitated some adaptations; however, disruptions persisted due to systemic inequities and insufficient mechanisms to protect marginalized populations. Middle- and low-income countries experienced more substantial service interruptions, largely attributed to constrained resources, limited system flexibility, and inconsistent policy implementation.

The most effective responses were supported by pre-existing policies that enabled telemedicine, multi-month medication dispensing, and decentralized delivery models. These findings align with the review’s aim to examine HIV service disruptions across the care continuum and identify cross-cutting barriers, facilitators, and adaptation strategies. They underscore the importance of equity-oriented policy frameworks that institutionalize differentiated HIV service models, ensure continuity of care for priority populations, and integrate HIV services into broader emergency preparedness plans. Policy environments that prioritize system adaptability, access, and inclusivity are essential for maintaining HIV care during future public health emergencies. Integrating these strategies into routine HIV care delivery may enhance service continuity and support preparedness for future public health emergencies.

## Strengths and limitations

This systematic review presents several methodological and analytical strengths. The study offers a comprehensive synthesis of the impact of the COVID-19 pandemic on all phases of the HIV care continuum, disaggregated by country income levels. Stratification by low-, middle-, and high-income contexts enabled context-specific interpretation of service disruptions, resilience patterns, and adaptation strategies. The integration of quantitative and qualitative evidence using a convergent integrated approach allowed for a more holistic assessment of both measurable outcomes and experiential dimensions of care. Alignment with the JBI methodology for mixed methods reviews and adherence to PRISMA 2020 reporting standards strengthened the methodological rigor and transparency of the review process.

Critical appraisal of included studies using JBI tools, conducted independently by multiple reviewers, ensured consistent evaluation of study quality. The use of a seven-domain framework to organize findings along the HIV care continuum facilitated structured synthesis and cross-domain comparison. Moreover, the identification of barriers and facilitators across country contexts provided valuable insights for informing future HIV service adaptation strategies under public health emergencies.

Several limitations should be considered. The inclusion of studies published in English or translatable with adequate accuracy may have resulted in language bias, potentially excluding relevant evidence from non-English-speaking regions. The exclusion of intervention studies, while purposeful to focus on naturalistic service disruptions, limits insight into the effectiveness of mitigation strategies. Substantial heterogeneity in study design, population characteristics, and outcome measurement limited the potential for meta-analysis and required reliance on narrative synthesis. In handling mixed-methods integration, discordant findings between quantitative and qualitative data were synthesized thematically, though methodological limitations in some studies may have influenced the degree of integration.

Data representation was uneven across income groups, with a higher proportion of studies originating from high- and middle-income countries. Moreover, key populations such as migrants, incarcerated individuals, and other marginalized groups were underrepresented, limiting the generalizability of findings to these subgroups. In addition, the reliance on peer-reviewed sources may have contributed to publication bias, potentially underrepresenting studies with null or negative findings.

## Supplementary Information

Supplementary material 1.

Supplementary material 2.

## Data Availability

No datasets were generated or analysed during the current study.

## Abbreviations

ART: Antiretroviral therapy
COVID-19: Coronavirus disease 2019
GNI: Gross national income
HCC: HIV care continuum
HIC: High-income countries
HMA: HIV medical appointments
HTS: HIV testing services
HTE: HIV treatment engagement
JBI: The Joanna Briggs Institute
LIC: Low-income countries
LRC: Linkage/receipt to care
MIC: Middle-income countries
MMD: Multi-month dispensing
MMSR: Mixed-methods systematic reviews
PAR: People at risk of HIV
PEPFAR: The Presidential Emergency Plan for AIDS Relief
PLWH: People living with HIV
PrEP: Pre-exposure prophylaxis
PRISMA: Preferred Reporting Items for Systematic Reviews and Meta-Analyses
QA: Quality assessment
VCT: Voluntary counseling and testing
VS: Viral suppression
