# Supplementary material for: Evaluating the impact of COVID-19 on the HIV care continuum across global income levels: a mixed-methods systematic review
Source: AIDS Res Ther. 2025 Oct 28;22:115. doi: 10.1186/s12981-025-00778-w (PMC12560595; doi:10.1186/s12981-025-00778-w)
Supplement: Supplementary file 1 — Supplementary material 1. [file 12981_2025_778_MOESM1_ESM.docx]

**Appendix I:** Search strategy

Ovid Embase

Date searched: June 19, 2022

Search update: January 12. 2024

| Search  number | Keywords |
| --- | --- |
| 1 | exp Human immunodeficiency virus/ |
| 2 | exp acquired immune deficiency syndrome/ |
| 3 | Human immunodeficiency virus infected patient/ |
| 4 | anti human immunodeficiency virus agent/ |
| 5 | attitude to AIDS/ |
| 6 | hiv or acquired human immunodeficiency syndrome or human immunodeficiency virus).mp. [mp =title, abstract, heading word, drug trade name, original title, device manufacturer, drug manufacturer, device trade name, keyword heading word, floating subheading word, candidate term word] |
| 7 | ((exp Coronavirus/ or exp Coronavirus Infections/ or (coronavirus∗ or corona virus∗ or OC43 or NL63 or 229E or HKU1 or HcoV∗ or ncov∗ or covid∗ or sars-cov∗ or sarscov∗ or Sars-coronavirus∗ or Severe Acute Respiratory Syndrome Coronavirus∗ or D614G).mp.) not (SARS or SARS-CoV or MERS or MERS-CoV or Middle East respiratory syndrome or camel∗ or romedary∗ or equine or coronary or coronal or covidence∗ or covidien or influenza virus or HIV or bovine or calves or TGEV or feline or porcine or BcoV or PED or PEDV or PDCoV or FIPV or FcoV or SADS-CoV or canine or Ccov or zoonotic or avian influenza or H1N1 or H5N1 or H5N6 or IBV or murine corona∗). mp.) or coronavirus disease 2019/ or (((pneumonia or covid∗ or coronavirus∗ or corona virus∗ or ncov∗ or 2019-ncov or sars∗).mp. or exp pneumonia/) and Wuhan.mp.) or (“coronavirus disease 2019” or 2019-ncov or ncov19 or ncov-19 or 2019-novel CoV or severe acute respiratory syndrome coronavirus 2 or sars-cov2 or sarscov- 2 or sarscov2 or sarscov-2 or Sars-coronavirus2 or Sars-coronavirus-2 or SARS-like coronavirus∗ or coronavirus-19 or covid19 or covid-19 or “covid 2019” or “B.1.1.7” or “B.1.351” or “B.1.617.1” or “B.1.617.2” or (variant∗ adj2 (India∗ or “South Africa∗” or UK or English or Brazil∗ or alpha or beta or delta or gamma or kappa or lambda or “P.1” or “C.37”)) or ((novel or new or nouveau) adj2 (CoV or nCoV or coronavirus∗ or corona virus))).mp. |
| 8 | 1 or 2 or 3 or 4 or 5 or 6 |
| 9 | 7 and 8 |
| 10 | limit 9 to dc =20200101-20221231 |

∗Special OVID-Embase Filter for COVID-19
