# Supplementary material for: Evaluating the impact of COVID-19 on the HIV care continuum across global income levels: a mixed-methods systematic review
Source: AIDS Res Ther. 2025 Oct 28;22:115. doi: 10.1186/s12981-025-00778-w (PMC12560595; doi:10.1186/s12981-025-00778-w)
Supplement: Supplementary file 2 — Supplementary material 2. [file 12981_2025_778_MOESM2_ESM.docx]

**Table 1.** Comprehensive details of the included articles

| NO | **Author, year, country** | **Study method and design** | **Sample size** | **Results** | | **Barriers** | **Facilitators** |
| --- | --- | --- | --- | --- | --- | --- | --- |
|  |  |  |  | **Stage of care continuum*** | **COVID impact on HCC stage** |  |  |
| 1 | (Galaviz et al. 2022)  USA | Quantitative, Cross-sectional | 101 | HMA, HTS, AA | HMA: 40% delay in follow-up visits; HTS: 35% difficulty in testing for viral load; AA: 21% difficulty accessing ART | NA | Telemedicine |
| 2 | (Bogart et al. 2021)  USA | Quantitative, Cross-sectional | 101 | AA | AA: Lower ART adherence among those heavily and negatively impacted by the pandemic. | High medical mistrust toward COVID-19 as a barrier to HIV care, reduced working hours (33%), inability to pay bills, rent, or utilities (29%), less access to public transportation (25%), barriers potentially affecting ART access | NA |
| 3 | (Chang et al. 2022)  USA | Quantitative, Cross-sectional | 4,193,334 | HTS | HTS: 29% decline in HIV testing rate; 24% decrease in HIV diagnoses during the pandemic. | changes in care delivery, testing shortages, and reduced care-seeking behaviors |  |
| 4 | (Chow et al. 2020)  Australia | Quantitative, Cross-sectional | 204 | PP | PP: 73% continued daily PrEP use during lockdown; 4.5% switched to on-demand; 22.5% discontinued use. | Difficulty scheduling PrEP appointments, fear of contracting COVID-19 at clinic visits during lockdown | Sexual health centers remained open for HIV testing and post-exposure prophylaxis, lockdown seen as opportunity to end HIV, main reason for stopping PrEP: reduced casual sex or fewer casual partners |
| 5 | (Di Ciaccio et al. 2022)  France | Quantitative, Cross-sectional | 8345 | HTS, PP | HTS: 32.3% tested for HIV/STIs post-lockdown vs 70.2% pre-lockdown; PP: 68.5% used PrEP on demand pre-lockdown vs 44.8%; fewer sexual partners and more stable partnerships reported during lockdown. | 86.5% stopped PrEP due to no sexual activity, 8.5% chose to stop PrEP, 3.6% ran out of medication, 5.9% had cancelled or postponed PrEP appointments,  9.4% stopped for other reasons | NA |
| 6 | (Gillespie et al. 2022)  Wales, UK | Quantitative, Cross-sectional | 60 | PP | PP: Gradual decline in PrEP use; 51–63% of CAS episodes covered by adequate PrEP among daily users; high individual variation in use and risk behaviors. | Reduced PrEP use due to pandemic-related controls | NA |
| 7 | (Hongsermeier-Graves et al. 2022)  USA | Quantitative, Case series | 37 | NA | NA | Overrepresentation of Hispanic patients, non-citizens, refugees, and undocumented immigrants among PWHC | NA |
| 8 | (Joseph et al. 2022)  USA | Qualitative, Descriptive | 32 | HMA, AA | HMA: Most remained in close contact with physicians; AA: Pharmacy access issues and appointment disruptions; 72% reported no difficulty with ART access or adherence. | Cancelled appointments, decreased healthcare access, disruption of care routine, pharmacy access, exacerbation of medical conditions | Pharmacy shipping medications for remote patients, proactive patient engagement by providers, increased access through telemedicine, education, and patient empowerment |
| 9 | (Krist et al. 2022)  Netherlands | Quantitative, Cohort | 319 | HTS, PP | PP: PrEP discontinuation rose from 2.3% to 16.9%; daily use increased from 33.7% to 36.8%; HTS: HIV testing in past 3 months dropped from 92.4% to 69.8%. | NA | NA |
| 10 | (D. Lee et al. 2021)  Australia | Quantitative, Cross-sectional | 4551 | AA; VS | AA: ART postal delivery rose to 14% in 2020; MPR remained stable and >1 across years; no ART shortages reported; VS: Controlled viral load remained high (2018–2020) with no significant decline. | NA | ART delivery via postal service for a small fee, telehealth used to monitor and manage PLWH |
| 11 | (J.-A. Lee, Kim, and Choi 2021)  Korea | Quantitative, Cross-sectional | 286: 112 PLWH and 174 people at risk of HIV (PAR) | HMA, HTS, AA, PP | HMA: 17% of PLWH and 59.2% of PAR reduced or stopped clinic visits; prescribers reported 44.4% (PLWH) and 77.8% (PAR) visit decline due to closures; HTS: HIV-related testing declined for 6.3% of PLWH and 50.6% of PAR; ART: 12.5% of PLWH faced ART interruptions; 44.4% of prescribers noted reduced ARV refills; PP: 25% of PAR reduced and 25% discontinued HIV prevention drug use; 14.3% of prescribers reported limited access. | Fear of contracting COVID at hospital/clinic, travel constraints | Engaging in less risky behavior |
| 12 | (Levy et al. 2022)  Israel | Quantitative, Cross-sectional | 1194 | PP, HTS | PP: 66.7% reported fewer casual sex partners; PrEP users had higher risky behavior scores; 45% maintained pre-lockdown PrEP use.  HTS: 26.9% received less medical follow-up and fewer HIV/STD tests during lockdown. | Reduced availability of medical services | Less sexual behaviors with fewer partners |
| 13 | (Liu et al. 2022)  Taiwan | Quantitative, Cross-sectional | 66 PLWH, 104 individuals at risk of HIV infection (IAR), 32 prescribers | HMA, HTS, ART, PP | HMA: Clinic visit interruptions reported by 31.3% (PLWH) and 59.4% (IAR) of prescribers; self-reported by 13.6% (PLWH) and 29.8% (IAR); HTS: Reduced HIV testing access seen by 31.3% (PLWH) and 40.6% (IAR) of prescribers; self-reported by 3.0% (PLWH) and 24.0% (IAR); ART: 28.1% of prescribers saw reduced ART refills; 9.1% of PLWH reported ART interruptions; 28.8% were concerned about ART access; PP: 10.5% of IAR reduced preventive drug use; 29% were concerned about future access; 53.1% (PLWH) and 42.3% (IAR) reported fewer/no sexual partners. | Fear of contracting COVID-19 in hospitals/clinics, temporary cancellation of evening clinics and refills, longer hospital visits due to control measures, travel restrictions, privacy concerns from contact tracing | Reduced engagement in high-risk behaviors, improved ART access through info on community pharmacies, medication shipping for overseas patients |
| 14 | (Corneli et al. 2022)  USA | Qualitative, Descriptive | 20 | PP | PP: Reduced risky sexual behavior and fewer partners; PrEP use continued among sexually active men and all women; no reported access issues; provider communication remained strong. | Decreased sexual activity led to stopping/reducing PrEP, pandemic distractions affected PrEP adherence, restricted in-person clinic visits, inadequate telecommunication (felt rushed or uncomfortable) | Maintained daily routine, followed healthcare provider recommendations, PrEP cost covered by insurance |
| 15 | (Dark et al. 2022)  USA | Qualitative, Descriptive | 10 | LRC, HMA | LRC, HMA: Some clinics increased new patient referrals and ensured prompt care for new diagnoses; limited in-person visits and challenges with routine lab testing reported; some YLWH re-engaged in care. | Limited time to prepare clinics for COVID-19 restrictions, lack of transportation, financial burden, stress from the pandemic and social justice issues, limited virtual visits due to technical, scheduling, and privacy issues | Quick adaptations by providers and administrators, use of telehealth, clinics covered lab costs not paid by insurance |
| 16 | (Devlin et al. 2022)  USA | Qualitative, Descriptive | 25 | HMA, AA, LRC, HTS | HMA: Appointment cancellations reported by HIV care physicians; AA: Mixed ART adherence, some reported difficulties, others became more adherent; HTS, LRC: One woman diagnosed with HIV and linked to care due to pandemic-driven symptom evaluation. | Fear of contracting COVID, the stress of the pandemic, financial burden, and food insecurity. | Telehealth, prescription delivery, pandemic forced some to take their health (and HIV care) more seriously |
| 17 | (Pampati et al. 2021)  USA | Quantitative, Cross-sectional | 78 | PP, HTS | PP, HTS: 19.2% discontinued or changed PrEP; 25.6% faced challenges accessing PrEP, HIV, or STD testing; decrease in high-risk sexual behavior reported. | Social distance, fear of getting COVID-19 | At-home HIV/STD testing, self-testing for HIV via an oral swab-based test, telemedicine |
| 18 | (Quiros-Roldan et al. 2021)  Italy | Quantitative, Retrospective | 130 | HTS | HTS: 31.2% decline in HIV diagnosis | Stopping HIV screening in some community-based organizations and health clinics | NA |
| 19 | (Torres et al. 2021)  Brazil | Quantitative, Cross-sectional | 814 | AA | AA: 18.2% poor ART adherence | Social distancing, fear of going out, non-availability of public transportation | NA |
| 20 | (Harkness et al. 2022)  USA | Quantitative, Retrospective | 20 | HTE, AA, PP | AA, HTE:100% inherent to HIV care and ART service; PP: 30% reported using behavioral health services | Stay safe home, closure of HIV centers due to pandemic, difficulty in using system for getting behavioral health services, unawareness about insurance coverage | Telehealth, free public transportation |
| 21 | (Lesko et al. 2022)  USA | Quantitative, Cohort | Observations 1286 persons 773 | HMA, AA | HMA: 13% missed an HIV visit, AA: 19% missed at least one dose of ART | For HMA: unstable housing, food insecurity, anxiety, low resiliency, mental health care disruption, substance use (cigarette, alcohol, cocaine, cannabis); for AA: being male, low resiliency, mental health care disruption, cigarette smoking, alcohol use, cocaine, cannabis, previous disruption in substance use treatment | Telehealth, telephone visits |
| 22 | (Wenlock et al. 2022)  UK | Quantitative, Cohort | Pre-pandemic 120,066 pandemic 16,458 | HTS | HTS: 64% drop in HIV testing and 66% drop in diagnoses in sexual health services; testing remained stable in secondary care, dropped in specialist services, and increased 4-fold in emergency departments (mainly elderly); 50% fewer new diagnoses in secondary care. | Lockdown | Self-testing (mostly by women and younger individuals) |
| 23 | (Vanbaelen et al. 2022)  Belgium | Mixed-method, Cross-sectional | 1073 | PP | PP: 143 (13.3%) patients discontinued PrEP care | Stopping PrEP use (difficulties to maintain the PrEP schedule and access clinic), COVID-19, decreased sexual activity due to COVID-19 and not COVID-19 related, monogamous relationship, consistent condom use, experiencing side effects | NA |
| 24 | (Zubiago et al. 2021)  USA | Quantitative, Cross-sectional | 8126 | HTS | HTS: HIV testing rose from 10.4% (Jan 2020) to 28.2% (Apr 2020), then declined to 12% (Aug 2020); increase during pandemic peak followed by post-peak drop. | NA | NA |
| 25 | (Howarth et al. 2022)  UK | Quantitative, Cross-sectional | 2018 | HTS | HTS: Reduced unmet need for STI testing vs 2017; shift from face-to-face testing to self-sampling during the pandemic. | Social distancing, financial hardship, interruption of clinical services | Decrease in sexual partnership |
| 26 | (Eckardt, Niu, and Montalvo 2021)  USA | Quantitative, Cohort | 206 | HTS | HTS: Monthly average dropped from 1,745 to 726 during the pandemic (statistically significant). | Social, economic, and psychological factors | Opt-out emergency room testing |
| 27 | (Stephenson et al. 2021)  USA | Quantitative, Cross-sectional | 518 | PP, HTS | PP: 9% reduction in accessing PrEP prescriptions; HTS: 32.2% reported the pandemic prevented HIV testing. | Social distancing | NA |
| 28 | (Quiros-Roldan et al. 2020)  Italy | Quantitative, Cohort | 3875 | HTS. HMA, AA, | HTS: New HIV diagnoses dropped from 6.4 to 2.5 per month; HMA: Missed outpatient HIV visits increased from 5% to 8%; AA: ART dispensation decreased by 23.1%. | Social distancing, quarantine, movement restrictions, reduced access to routine HIV testing | NA |
| 29 | (El-Nahal, Shen, Keruly, Jones, Fojo, Lau, et al. 2022)  USA | Quantitative, Cohort | 1834 | HMA | NA | NA | Telemedicine mitigate barriers to care, 20-39 age group, women, black patients, and patients with detectable viremia. |
| 30 | (Rick et al. 2021)  Brazil | Quantitative, Cross-sectional | 847 | PP, HTS | PP: 7% reported more sexual partners, 6% reported using condoms less often, 7% withdrew HIV PrEP, 48% of participants reported not knowing what PrEP was. HTS: 5% reported difficulties obtaining HIV tests. | Difficulty in accessing service-based HIV rapid test, lockdown | Access to expedited test results, avoiding physical attendance at health units, privacy and trust in HIV self-testing with a known perso |
| 31 | (Sanchez et al. 2020)  USA | Quantitative, Cross-sectional | 1051 | PP, HTS, LRC, HMA | PP: 51.3% reported fewer sex partners; 68% had fewer opportunities for sex; 9.4% reported reduced condom access.  HTS: 18.8% reported reduced HIV testing access.  LRC: 8.2% had difficulty getting ART prescriptions; 6.6% had difficulty obtaining ART meds.  HMA: 19.7% had trouble making or keeping HIV appointments. | Lockdown, loss of income, loss of health insurance or housing | Reduced HIV/STI transmission risk due to less high-risk sexual behavior |
| 32 | (Rhodes et al. 2021)  USA | Quantitative, Retrospective | 15 | PP, HMA, AA | PP: Preventive behaviors increased due to reduced social contact and staying home; AA: No changes reported; HMA: Visits canceled or postponed; dissatisfaction with telemedicine. | Social isolation, economic stability, access to health care, | NA |
| 33 | (Hammoud et al. 2021)  Australia | Quantitative, Cohort | 847 | PP; HTS | PP: 41.8% discontinued PrEP after restrictions began; HTS: HIV testing remained stable (36.9% pre-pandemic vs 40.3% during pandemic) among PrEP users. | Need to monitor long-term impact of COVID-19 on GBM sexual behavior, potential changes in prevention coverage and HIV risk | NA |
| 34 | (Tamargo et al. 2021)  USA | Quantitative, Cohort | 299 | PP, HMA, AA | HMA: 6% missed HIV appointments; 2% had cancellations; AA: 13.2% avoided ART pickup; 8.2% missed ≥1 dose in past month; PP: 66.7% of HIV-uninfected missed a PrEP dose in past month. | Avoidance of public transportation, fear of COVID-19 infection, social distancing measures | NA |
| 35 | (El-Nahal, Shen, Keruly, Jones, Fojo, Manabe, et al. 2022)  USA | Quantitative, Cohort | 8,785 | VS | VS: Viral load follow-up in suppressed patients dropped from 91% (pre-pandemic) to 59% (lab closed), then rose to 87% (lab open); in non-suppressed patients: 90% to 75%, then up to 88%. | Anxiety about catching COVID-19, loss of insurance, financial insecurity | NA |
| 36 | (McGinnis et al. 2021)  USA | Quantitative, Cohort | 27,674 | HMA, AA, VS, HTS | HMA: No change in clinic visit, 37% increase in virtual visit during pandemic; AA: No change in ART adherent,12% increase in longer refills for ART; VS: No change in suppressed viral load;  HTS: 8% reduction in viral load test | NA | NA |
| 37 | (Chow et al. 2021)  Australia | Quantitative, Cohort | 71,361 | PP, HTS | PP: PrEP prescriptions dropped by 68% in Melbourne and 60% in Sydney; HTS: HIV testing fell by 41% (Melbourne) and 32% (Sydney); new HIV diagnoses declined by 44% and 47%, respectively. | NA | NA |
| 38 | (Weerasuria et al. 2021)  Australia | Quantitative, Cross-sectional | 153 | AA, LRC | AA: 98% were able to access ART; LRC: 55% were able to access HIV provider during the pandemic | NA | Access to HIV care facilities during lockdown |
| 39 | (Wion and Miller 2021)  USA | Quantitative, Cross-sectional | 85 | LRC | LRC: Low moderate difficulty in communicating with providers about HIV-related concerns. | Difficulty in accessing healthcare settings | NA |
| 40 | (Keane et al. 2022)  Ireland | Quantitative, Cohort | 546 patients, 1660 HIV tests on MSM | PP, HTS | PP: 19.4% altered PrEP dosing schedule; HTS: New HIV diagnoses decreased from 95 to 73. | NA | Significant decline in HIV positive cases during the pandemic, decline partly linked to launch of free PrEP program |
| 41 | (Nguyen et al. 2021)  USA | Quantitative, Cross-sectional | 100 | AA, HTE | AA: 24% missed a dose of HIV medication; HTE: 46% experienced disruptions to health care | Financial changes due to COVID-19, younger ages | NA |
| 42 | (Qiao, Li, et al. 2021)  USA | Mixed-method, cross-sectional & phenomenological | 27 HIV clinic | HMA | HMA: 56% of HIV clinics partially interrupted; 26% experienced complete closure. | Limited access to computer or internet to benefit from telehealth | Telehealth and mobile applications |
| 43 | (MacNeill et al. 2022)  USA | Qualitative, Descriptive | 15 | HTE, VS, LRC, HTS | HTE/VS: Staff concerned that lack of in-person contacts impacted ART adherence and viral suppression; LRC: Pandemic-related unemployment enabled service access for some; new intakes and face-to-face encounters were restricted; HTS: Significant drop in HIV testing during limited in-person access. | Perceived increased risk of mortality from COVID, lack of PPE, geographic and technological limitations, pre-existing mental health conditions, social isolation, unemployment, lack of preparedness by organizations, COVID restrictions | Unemployment allowed more time for health management, organizational preparedness (early ID of high-risk clients), self-swab tests and outdoor testing, telehealth and Zoom for virtual testing and education, paid phone bills to support virtual care, provided tablets for clients, supply drop-offs in accessible open areas, government and private sector funding for initiatives |
| 44 | (Mazzitelli et al. 2021)  Italy | Quantitative, Cohort | 131 | HTS, VS | HTS: AIDS-defining illnesses diagnosed in 31.2% (pre-COVID) vs 11.1% (COVID period) of PLWH; VS: Lower CD4+ counts and higher HIV-RNA levels at diagnosis; 66.2% (pre-COVID) vs 57.4% (COVID) were late presenters. | NA | NA |
| 45 | (Moitra et al. 2022)  USA | Quantitative, Cross-sectional | 40620 | HTS | HTS: HIV testing declined sharply in outpatient settings, then increased post-lockdown; 27–59% decline in four metro areas; 65% drop in community testing in New Orleans; smaller declines in ED testing in Minneapolis (13%) and Seattle (17%). | Stay-at-home orders | Lifting of stay-at-home orders, overlapping HIV and COVID symptoms and social determinants, increased symptomatic screening in emergency departments, ED remained open, driving HIV testing there over closed clinics |
| 46 | (B. G. Rogers et al. 2022)  USA | Quantitative, Cross-sectional | 177 | PP | PP: 2.72 fewer sexual partners and 1.65 fewer condomless anal sex partners reported during plateau phase vs pre-COVID; no significant change in PrEP adherence. | Limited social contact | NA |
| 47 | (B. G. Rogers et al. 2021)  USA | Quantitative, Cross-sectional | 600 clinic visits | PP | PP: Evolving Phase, 6% drop in overall PrEP visits, 44% drop in initial visits, no change in follow-ups; Plateau Phase, 16% drop in overall visits, 49% drop in initial visits, 12% drop in follow-ups; no substantial declines overall. | Difficulty accessing PrEP due to COVID-19 restrictions | Clinics allowed PrEP initiation after required lab tests, continued PrEP refills for regular users |
| 48 | (Niu, Sareli, and Eckardt 2022)  USA | Quantitative, Cohort | 45185 | HTS | HTS: 54.7% drop in monthly opt-out HIV tests from pre-pandemic to pandemic period; sharpest decline in March 2020; gradual monthly increase observed, returning to pre-pandemic levels by October 2020. | Decrease in ED visits | NA |
| 49 | (Norwood et al. 2022)  USA | Quantitative, Cross-sectional | 1686 | HMA, LRC, VS | HMA: 33% drop in medical interactions in 2020 vs 2019 (−827 visits); LRC: 23.5% decline in new patient interactions in Q2 2020 vs 2019; VS: Viral suppression dropped from 83.4% to 76.9%. | Decreased community HIV testing, patients avoided testing to reduce COVID-19 exposure, reduced testing capacity due to staffing shortages | Increased utilization of telehealth |
| 50 | (Petrova et al. 2022)  USA | Qualitative, Descriptive | 12 | AA | AA: 3.30% decreased in ART adherence | Required in-person pharmacy visits for medication; COVID-19 protocols as barriers, lack of daily structure in life | Curtailed substance use, increased attention for personal health |
| 51 | (Shilo and Mor 2020)  Israel | Quantitative, Cross-sectional | 2562 | PP | PP: 11.5% increase in condom/PrEP use; 22.4% decrease in sexual risk behavior score during social distancing vs pre-distancing. | Being younger, single, prior high-risk sexual behaviors | Reduced physical contact with sexual partners; changes in sexual activities to avoid virus exposure |
| 52 | (Trepka et al. 2022)  USA | Quantitative, Cross-sectional | 298 | HMA, LRC | HMA: 7.6% of cisgender women and 13.2% of cisgender men reported more difficulty accessing case managers during the pandemic; LRC: Difficulty obtaining HIV drugs reported by 13.3% of Hispanics, 5.8% of Haitians, and 4.4% of non-Hispanic Blacks. | Transportation costs as a barrier to care, difficulty taking time off work for appointments, structural barriers for low-income and racial minority groups | Multiple options to access providers supported HIV care, diverse methods of ART delivery tailored to individual needs |
| 53 | (Zapata et al. 2022)  USA | Qualitative, Descriptive | 41 | PP, HTS | PP: Only 22% of eligible participants were using PrEP; reasons for discontinuation included appointment cancellations, celibacy, and service disruptions; HTS: Most participants lacked access to HIV testing due to reduced services, fear of COVID-19, financial hardship, or parental supervision. | Barriers to HIV prevention for YSMM; limited appointment availability; subpar service quality; structural and cultural healthcare disparities | NA |
| 54 | (Ejima et al. 2021)  Japan | Quantitative, Case-control | 31,076 (consultations) | HTS, HMA | HTS: 3.7-fold decrease in HIV tests, 9.8% reduction in new HIV cases with an AIDS diagnosis; HMA: 2.8-fold decrease in consultation | Missed more cases of HIV before developing AIDS | Use of self-testing |
| 55 | (El Moussaoui et al. 2021)  Belgium | Quantitative, Cohort | 1162 | HTS, HMA, VS | HTS: 46% drop in number of new HIV diagnoses; HMA: 17% decrease in number of medical visits; VS: Proportion of patients with HIV VL >400 copies/mL dropped from 9% to 5% in 2020. | Closure of screening centers, reduced public transportations, high workload of healthcare facilities | Telemedicine |
| 56 | (Gabster et al. 2022)  Panama | Quantitative, Cross-sectional | 960 | PP, HTS, HMA | PP: 45.6% difficulty finding condoms during COVID-19 measures; HTS: 58% of those needing an HIV test (10.4%) were unable to get one; HMA: 53% cancelled or postponed HIV care appointment. | Limited transportation, testing facility closures or the covidization of health services, lack of HIV self-testing and STI self-collection policies in Panama | NA |
| 57 | (Gaspar et al. 2022)  Canada | Qualitative, phenomenology | 25 | PP | PP: Some participants discontinued PrEP during the first lockdown due to reduced sexual activity; others continued use throughout. | Decreased sexual activity, lower risk due to fewer casual partners | Having various sexual partners |
| 58 | (Gwadz et al. 2021)  USA | Mixed-methods, Cross-sectional & Descriptive | 96 | AA, HTE, HMA | HTE: HIV management continued due to prior study involvement; AA: 90% take ART regularly, 2% stop ART, 40% increase in frequency of taking ART; HMA: several appointments were cancelled. | Social distancing, travel restrictions, avoidance of public transportation, limited internet access | Fear of COVID-19 infection, virtual appointments |
| 59 | (Hensley et al. 2022)  Netherlands | Quantitative, Cross-sectional | 41272 | HTS | HTS: 69% drop in potentially HIV-positive patients; 37% decrease in new HIV diagnosis referrals; 56% decline in weekly HIV testing. | Reluctance to leave home | NA |
| 60 | (Hill, Anderson, and Lock 2021)  USA | Quantitative, Retrospective cross-sectional | 12102 | PP, HTS | PP: New patients made up 31.3% of PrEP visits in 2019 vs 16.3% in 2020; HTS: 58.7% decrease in HIV testing compared to pre-pandemic period. | NA | NA |
| 61 | (Hong et al. 2022)  USA | Quantitative, Cross-sectional | 239 | PP, HTS | PP: 14.3% quit PrEP because of pandemic, 20% had trouble getting prescriptions from doctor, 8.6% had trouble getting prescription from pharmacy. HTS: 11.7% had trouble getting HIV test, 49.4% hadn't tried to get HIV test since pandemic began. | Lockdown restrictions, fear of getting COVID-19, difficulty obtaining prescriptions from pharmacy, trouble accessing HIV testing | NA |
| 62 | (Mistler et al. 2021)  USA | Quantitative, Cross-sectional | 110 | PP, HTS | PP: 25% had difficulty obtaining PrEP prescriptions; 20% stopped PrEP during the pandemic; HTS: 11.8% reported decreased HIV testing access; 77.3% hadn’t attempted testing since COVID onset. | Difficulties in obtaining appointments | NA |
| 63 | (Brown et al. 2022)  UK | Quantitative, Cross-sectional | 5066 | HTS | HTS: Recent HIV testing increased from 29.7% (P1) to 39.4% (P2) and 40.5% (P3) among HIV-negative/unknown status participants. | COVID-19 restrictions | NA |
| 64 | (Wu et al. 2022)  Taiwan | Quantitative, Cohort | NA | HTS, HTE, VS | HTS: HIV screening test increased; HTE: No change in engagement; VS: No significant change in viral suppression | Limited access to healthcare facilities, lockdown, reduced testing opportunities, shortened clinic opening hours, staff shortage, equipment shortage, travel restrictions | Telemedicine, Implementation of programs facilitating rapid and same-day ART initiation, multi-month dispensing and ART provision outside conventional healthcare facilities |
| 65 | (Unigwe et al. 2023)  USA | Quantitative, Cohort | 19,581 | PP | PP: Among 19,581 new PrEP users (96% male, 55% aged 18–34), adherence to recommended testing increased from 2016–2019 but declined during the COVID-19 pandemic. | COVID-19 restrictions | NA |
| 66 | (Davis et al. 2023)  USA | Quantitative, Cross-sectional | 196 | LRC | HMA: Pre-pandemic challenges included connecting to resources and scheduling; during the pandemic, added difficulties included provider communication and wait times. | Transportation, healthcare cost, feeling supported by healthcare system | Peer navigation telehealth |
| 67 | (Schmidt et al. 2023)  USA | Quantitative, Cohort | 625 | PP | PP: Eligibility among PrEP users ranged from 81.3%–91.1% pre-pandemic vs 70.5%–81.4% early-pandemic; overall eligibility was 92.6% pre-pandemic and 90.2% early-pandemic. | COVID-19 restrictions | Telehealth |
| 68 | (Hazell et al. 2024)  UK | Quantitative, Cohort | 176 | LRC, VS | LRC: Face-to-face visits dropped from 87.6% (Period 1) to 71.3% (Period 2); mean F2F visits per patient fell from 3.55 to 2.19; 168-day median gap between periods; VS: Viral suppression increased from 83.5% pre-lockdown to 88.6% during lockdown. | Low mood and anxiety | NA |
| 69 | (Carbonero-Lechuga et al. 2023)  Spain | Quantitative, Cross-sectional | 25 | AA, VS | AA: 12.5% of previously non-adherent patients became adherent after the pandemic began; VS: Detectable viral load decreased from 36% to 24%; 80% of participants had reached AIDS stage. | COVID-19 restrictions and lockdown | Greater amount of experience that older patients have with the disease, having adapted the treatment to fit into their daily lives |
| 70 | (Rosen et al. 2022)  USA | Qualitative, Descriptive | 20 | PP | PP: Providers reported decreased PrEP demand, high discontinuation, and loss to follow-up in year one of COVID-19; linked to changes in sexual behavior and shifting priorities due to mitigation measures. | Structural barriers, including outreach service suspension, personnel shortages, and facility restrictions on face-to-face visits | Telemedicine |
| 71 | (McKay et al. 2023)  Canada | Qualitative, Descriptive | 18 | HTE | HTE: Among ACB WLWH, access, affordability, and motivation to use HIV care were affected; virtual care felt dismissive for some, but disengagement was rare; fear of COVID-19 and misinformation reduced motivation. | Felt dismissed by virtual-only healthcare providers, preference for hybrid care models, reduced access to mental health supports and groups, affordability concerns beyond provincial health coverage | NA |
| 72 | (Harkness et al. 2022)  USA | Mixed-methods, Cross-sectional & Descriptive | 106 | HTS, PP | HTS: HIV service delivery innovations in case management (40%) and antibody testing (39.1%); PP: 32.6% reported impact on PrEP initiation. | Reduced staff availability and morale due to COVID outbreaks and deaths, social isolation, layoffs, and fear of infection affected providers; lack of PPE and space hindered pandemic response efforts | Remote services (e.g., telehealth) enhanced access during the pandemic |
| 73 | (Voisin et al. 2023)  USA | Qualitative, Descriptive | 28 | HMA, HTS, AA, LRC | HMA: Decrease in in-person visits; virtual visits tolerated with dissatisfaction, but no major changes reported; HTS: Decrease in HIV testing; some went six months without testing; AA: Mixed ART adherence, some forgot doses, others improved due to more self-care time; LRC: No changes reported due to virtual visits. | Pandemic restrictions, over-the-phone visit, disruption of daily routine by working from home, staffing disruptions, need longer time to plan for medical appointment, confusion about effectiveness of ART, concomitant racial and social upheaval | More time for self-reflection, addressing unresolved issues with family contributing to more stable mental and emotional state, deeper self-care, telemedicine |
| 74 | (Bleasdale et al. 2022)  USA | Qualitative, Descriptive | 25 | HTE, AA, HMA | HTE: 64% reported limited care engagement and difficulty receiving needed care; HMA: 52% experienced disruptions in medical appointments; family support encouraged continued care; AA: 56% reported reduced willingness/ability to take ART; 84% maintained adherence due to social support. | Unstable income, being unemployed because of COVID-19, inadequate housing, food insecurity, contracting COVID-19, poor internet connection | Social support from friends, family, social service providers, and clinicians, telehealth |
| 75 | (Kalichman et al. 2023)  USA | Quantitative, Cohort | 140 | HMA, AA | HMA: 33% missed healthcare appointments; 66% reported provider-cancelled visits; AA: 20% experienced interruptions in accessing ART. | COVID-19 restrictions, lack of transportation, unstable housing, and food insecurity | Social support |
| 76 | (Barish et al. 2023)  USA | Quantitative, Cross-sectional | NA | HTE, HTS | HTE, HTS: In-person care dropped by 85.7% in Wave 1 and 21.4% in Wave 5 vs pre-pandemic; virtual care increased by 100% (Wave 1) and 42.9% (Wave 5); other services declined in Wave 1 but stabilized by Wave 5. | Structural barriers, inadequate infrastructure | Telecommunication |
| 77 | (Palacio-Vieira et al. 2023)  Spain | Quantitative, Cohort | 15,841 | HTE | HTE: increased loss to follow-up during pandemic | Being PWID, younger PLH, pandemic restrictions | NA |
| 78 | (De La Court et al. 2023)  Netherland | Quantitative, Cohort | 305 | PP, LRC | LRC and PP: significantly more than before pandemic | Even-driven PrEP using | Not worrying about COVID-19 infection, having chemsex, using daily PrEP |
| 79 | (Hentges et al. 2024)  Sweden | Quantitative, Cross-sectional | 1,138 | PP, HTS | PP: 23% less access to condom; HTS: 57% reduction in success to HIV/STI test | Being transgender or non-binary, COVID-19 restrictions, being foreign-born, financial problems, not functioning of postal service | NA |
| 80 | (Souleymanov et al. 2023)  Canada | Quantitative, Cross-sectional | 347 | PP, HTS | PP: 54,4% reduced access to condom; HTS: 27.7% reduced access to HIV testing | Living in medium-size city and in rural and remote areas, younger ages, health service reduction, professional' lack of competence | NA |
| 81 | (Camp, Chan, and Saberi 2023)  USA | Mixed-methods, Cross-sectional & Descriptive | 37 | PP | **PP: 75% used daily PrEP, 14% on 2-1-1, 11% discontinued; 60% of 2-1-1 users cited infrequent sex as reason; Qualitative: PrEP use paused due to reduced sexual activity, resumed with increased comfort post-vaccination and eased distancing.** | Limited in-person STI screening and PrEP services, young adults avoided telehealth due to privacy concerns at home | NA |
| 82 | (Hou et al. 2023)  China | Quantitative, Cross-sectional | 1017 | PP | PP: 23.6% reported sexual needs; ~20% were sexually active; loneliness linked to higher risk of heterosexual HIV transmission. | Access to smartphones, loneliness | NA |
| 83 | (Labban et al. 2023)  Saudi Arabia | Quantitative, Cross-sectional | 85 | AA, LRC | AA: 58.8% reported no change in ART adherence; 37.7% reported increased adherence; 3.5% reported decreased adherence; LRC: 63.5% accessed clinics without disruption; 36.5% faced disruptions; 10.6% couldn’t reach providers; 89.4% could disclose their providers. | NA | Phone clinic launched for PLWH during lockdown, extended ART refills by HIV pharmacists, mail delivery of prescriptions by ambulatory pharmacies, drive-thru pharmacy for emergency supplies |
| 84 | (Platt et al. 2023)  USA | Quantitative, Cohort | 137 | PP | PP: 18.1% decrease in PrEP start during pandemic | COVID-19 lockdown | Being older, non-Hispanic White participants, flexible models of care |
| 85 | (Pan et al. 2024)  Spain | Quantitative, Cohort | 17,738 | HMA, PP, VS | HMA: 17.1% decrease in overall medical visits; PP: no change in primary care visits; VS: significant reduction in laboratory monitoring | Lockdown, lack of knowledge, staff shortage, HIV service repurpose for fight against COVID-19 | Telehealth, remained visits in primary care |
| 86 | (Ward et al. 2023)  USA | Quantitative, Cross-sectional | 298 | AA | AA: 12.7% reduction in ART adherence | Difficulty accessing HIV physician, financial issues | Telehealth |
| 87 | (Hong et al. 2023)  USA | Quantitative, Cohort | 371 | HTS | HTS: significant decline in HIV testing | Lockdown | NA |
| 88 | (Van Beckhoven et al. 2022)  Belgium | Quantitative, Cohort | 725 | HTS, HTE, VS, AA, PP | HTS: 17.6% decrease in HIV testing; 47.1% drop in acute HIV diagnoses; 24.7% decrease in late diagnoses; HTE: 2.7% experienced HIV care interruption; VS: Monthly decline in patients receiving VL monitoring; AA: No significant changes in ART adherence; PP: Drop in overall PrEP purchases, number of purchasers, and new starters. | Limited access to testing, decrease in casual sex partners, lockdown, limited availability of HIV services, fear of COVID-19, poverty | telemedicine |
| 89 | (Kamadjou et al. 2024)  France | Mixed-methods, Cross-sectional & Descriptive | 94 | PP | PP: 38% discontinued PrEP during the initial COVID-19 lockdown.  Qualitative: While many experienced the lockdown positively, symptoms of anxiety, sleep disorders, isolation, and emotional deprivation were commonly reported. | NA | Health system organization supported PrEP provision in Northern France during COVID-19 |
| 90 | (Rosas Cancio-Suárez et al. 2023)  Spain | Quantitative, Retrospective Cohort | 3265 | HTS, VS | HTS: New PLWH visits dropped to 3.97% during the pandemic vs 6.71% pre-pandemic and 5.25% post-pandemic; VS: 65.2% higher proportion of patients had detectable viral load pre-pandemic compared to during the pandemic. | Limitations in ART adherence and viral suppression for individuals aware of their HIV status due to COVID-19 lockdowns | NA |
| 91 | (S. A. A. Abraham et al. 2022) Ghana | Qualitative, Descriptive | 12 | LRC, AA, HMA | LRC: 100% linkage to care maintained as ART clinic remained open throughout the pandemic; HMA: 66.6% never missed or defaulted on appointments; AA: No ARV shortage initially, but some later reported location-based shortages. | Fear of COVID-19 exposure at clinics, ART shortages, added costs for PPE and medications, increased transportation costs, non-adherence to safety protocols by providers, clinic overcrowding and long wait times | ART clinic remained open for accessibility, increased education on COVID-19 protocols, four-month medication dispensing |
| 92 | (S. A. Abraham et al. 2021)  Ghana | Qualitative, Descriptive | 15 | HTS, AA, HMA | HTS, HMA: Reduced attendance and use of HIV testing, counseling, and ART services during early pandemic stages; AA: Drug stock-outs led to reduced ART quantities dispensed to patients. | Fear of contracting COVID, lockdown measures (i.e. closure of borders) | Switching ARV combinations supply to patients, modifying staff work schedules, establishment of different workstations along the care continuum |
| 93 | (Ahmed et al. 2021)  Pakistan | Qualitative, Phenomenological | 25 | AA | AA: difficulty in restocking ART supply | Stigma and discrimination, lockdown, lack of social support, economic constraints, fear of disclosure of HIV, forgetfulness, religion, adverse drug reactions, police abuse, insufficient transportation funds | Family responsibilities, reminders, telephone consultations, courier delivery of ART, using ART to decrease fear of contract COVID-19 and long-term drug delivery during COVID-19. |
| 94 | (Benade et al. 2022)  South Africa | Quantitative, Cross-sectional | 4,017,406 | LRC | LRC: 28% reduction in ART initiation | Level 5 lockdown in late March 2020, declaration of state of disaster in mid-March | NA |
| 95 | (Diaz et al. 2021)  Peru | Quantitative, Cross-sectional | 156 | AA, HMA, HTE | AA: 24% had difficulty picking up ART; 97% maintained regular ART use; HMA: 26% missed clinic appointments; HTE: 37.2% reported difficulty accessing routine HIV care. | Barriers to telemedicine in Peru: inconsistent internet and lack of equipment, difficulty contacting HIV clinic, lack of transportation and closure of HIV clinics, trouble communicating with the clinic | NA |
| 96 | (Lyu et al. 2021)  China | Quantitative, Cross-sectional | 731 | HTS | HTS: Pre-pandemic, 64.8% had ever tested for HIV; 82.1% used self-test kits. During the pandemic, 58.4% needed testing, but only 64.9% received it, 84.1% of which were through self-testing. | MSM with weaker community connections less likely to undergo HIV testing during COVID-19 | MSM with strong community connections more eager to undergo HIV testing during COVID-19 |
| 97 | Magnani  Indonesia  2022 | Quantitative, Cohort | 23 districts | HTS, PP, ART, LRC | HTS: 53% drop in health facilities and 100% drop in mobile clinics offering HIV testing; 85% fewer female sex workers tested and 83% fewer positive cases detected, LRC: 57% decrease in female sex workers reached by HIV programs; PP: 62% decrease in condom distribution, AA: 94% drop in ART initiation among HIV+ female sex workers. | Travel restrictions | Community testing introduced in 15 of 23 districts to counteract drop in HIV testing, CSO staff provided ongoing education, prevention support, and facilitated HIV testing, supported treatment initiation and retention during the first three months |
| 98 | (Matsuda et al. 2022)  Brazil | Quantitative, Cross-sectional | 3 regions | AA, LRC, HTS, PP | AA: ART dispensations fell by 14.5% and 5.9%; ART initiation dropped 19.3% and 4.6%; LRC: PLWH linkage to care increased by 4.4% and 4.8%, HTS: HIV testing dropped 20.4% and rose 18%; self-testing rose 204% and declined 45.6%, PP: PrEP dispensations rose 41.3% and 22%. | Cancellation of appointments and test collections due to lockdown | ART multi-month dispensing, HIV rapid self-test availability, national efforts to increase PrEP dispensation |
| 99 | (McCrimmon et al. 2022)  Kazakhstan | Qualitative, Descriptive | 24 | HMA | HMA: NSPs were forced to close at the pandemic's peak due to underfunding, compromising care for key populations. | Lack of resources, restricted access to personal protective equipment, poor salaries, and the designation of NSPs as nonessential services. | Flexibility of service providers (taking on new roles and responsibilities) |
| 100 | (McFall et al. 2022)  India | Quantitative, Cross-sectional | 13,854 | HTS, LRC, HMA | LRC: 25% drop in average client numbers during second wave vs first wave of the pandemic, HTS: 50% reduction in HIV testing during the pandemic vs pre-pandemic, HMA: Access to HIV providers declined for MSM in the second wave but remained stable for PWID. | Lockdown, avoiding public transport/being around people | NA |
| 101 | (Nguyen Thu et al. 2022)  Vietnam | Mixed-methods, Cross-sectional & Descriptive | 22 | HTS, HMA, LRC, AA | LRC: Reduced access to HIV/AIDS care, essential services, and financial protection for key populations, HTS: Inability to access HIV testing, AA: Inability to access ART, HMA: Medical appointments were canceled. | Stigma, fear of getting COVID-19, lacked adequate resources. | NA |
| 102 | (Nitpolprasert et al. 2022)  Thailand | Qualitative, Descriptive | 26 | AA | AA: adequate access to ART and able to maintain optimal ART adherence during the COVID-19 epidemic | Some participants hesitant to opt for home delivery of ART due to fear of HIV status disclosure | Home delivery of ART, fast-track service for ART refill at the clinic |
| 103 | (Pollard et al. 2021)  India | Qualitative, Descriptive | 44 | AA | AA: Mixed outcomes, some faced challenges with regular ART use, while others reported improved access to ART. | Difficulty traveling for HIV testing or ART pickup, lockdown, and restricted mobility, living with family and keeping HIV status secret, shortage of medication, income concerns | Door-delivery of ART |
| 104 | (Shi et al. 2022)  China | Quantitative, Cross-sectional | 436 | HTS, PP, LRC | HTS: 4.5% had trouble accessing facility-based HIV testing, only 7.6% had trouble accessing HIV self-testing kit. PP: 45% decreased number of sexual partners. 80.3% reported condom use stayed the same. LRC: 20.6% increased difficulty in accessing health care. | Fear of contracting COVID, mental health problems | NA |
| 105 | (N. K. Tran, Vu, and DeSilva 2022)  Vietnam | Qualitative, Descriptive | 32 | AA | AA: 12.5% reduction in ART access for MSM; no change reported for PWD. | Closure of public transportation, less income | NA |
| 106 | (X. Yang et al. 2022)  China | Quantitative, Cross-sectional | 1029 | LRC, HTS, AA | LRC: 5.1% of providers reported HIV clinic service suspension; 7.4% reported suspended/ postponed follow-up services; HTS: 53.9% reported suspension or postponement of VCT services; AA: 67.3% reported suspension/ postponement of ART application services; 0.5% reported ART provision delays due to short supply. | COVID-19 restrictions, suspended or postponed HIV centers and care | NA |
| 107 | (Ballivian et al. 2020)  Argentina | Quantitative, Cross-sectional | 1336 | HMA, AA | HMA: 34.7% unable to obtain telehealth care; AA: 96.1% had no problems obtaining medication. | Emotional distress among PLWH, limited capacity to manage health, inability to access medical care due to lack of telehealth technology, difficulty obtaining medications | Telehealth |
| 108 | (Booton et al. 2021)  China | Quantitative, Descriptive cross-sectional | 731 | PP, HTS, AA, VS | PP: 62% reduced number of sexual partners; 25% reported less condom use vs pre-pandemic; HTS: 59% reduction in facility-based HIV testing in 2020 vs 2019; AA: 34% decrease in ART initiation; VS: No change in viral suppression; viral load testing remained stable. | Realistic disruptions | Smaller impact on infections and deaths (<3%) from disruptions to HIV testing and ART initiation, reduced partner numbers led to 11% to 23% fewer infections and 0.4% to 1.0% fewer deaths |
| 109 | (Celestin et al. 2021)  Haiti | Quantitative, Quasi-experimental | 157,766 (consultations) | HMA, AA | HMA: Weekly clinical visits per facility dropped from 121.5 to 92.5; AA: 42.5% of ART dispenses occurred post-COVID; >6-month MMD rose from 29.4% to 48.4%; DAC rose from 22.7% to 36.7%; timely ART refills declined from 51.9% to 43.8%. | Primary and secondary effects | Multi-month ART dispensing and ART dispenses in community-based settings |
| 110 | (Dorward et al. 2021)  South Africa | Quantitative, Quasi-experimental | 1315439 tests | HTS, AA, LRC | HTS: 47.6% decrease in HIV testing; AA: no significant change in ART collection visits pre- vs post- lockdown; LRC: 46.2% decrease in ART initiation | Lockdown, disruption to ART collections | Being women |
| 111 | (Dyer et al. 2021)  Kenya | Quantitative, Cross-sectional | 486 | LRC, HTE | LRC, HTE: 17% could not access their regular clinic; 3% couldn’t get medication refills; no significant age-related differences reported. | Social distancing | Offering remote peer-support or mental health care, continuing to offer differentiated care services, and considering financial support will support the health and well-being of ALHIV. |
| 112 | (Hegarty et al. 2021)  Indonesia | Qualitative, Descriptive | 20 | LRC | LRC: linkage of care was reported from most participants | Healthcare was close due to pandemic, difficulty in travelling for doing test due to restrictions, longer distance to healthcare services | NA |
| 113 | (Htun Nyunt et al. 2021)  Myanmar | Quantitative, Cross-sectional | NA | PP, HTS, LRC, AA | PP: Slight reduction in prevention outreach activities - no change in syringe distribution and 1.98 in million reductions in free condom distribution; HTS: HIV outreach activities and HIV testing were slightly affected during pandemic - no changes in FSW and MSM, reduction in PWID; LRC: 40% reduction in ART initiation; AA: 40% reduction in ART adherent | Pandemic restrictions, ART facilities closure | Refill ARV for longer time |
| 114 | (Maurya et al. 2022)  India | Quantitative, Cross-sectional | COVID-19 Period = 1182; Preceding year = 2769 | HTS | HTS: 56.9% decline in HIV testing (48.8% decline of client-initiated testing, 60.3% decline in the provider-initiated testing) | Fear of COVID-19 exposure by visiting hospitals, restricted access for non-COVID-19 patients to prevent SARS-CoV-2 spread, COVID-19 care prioritized in healthcare settings, massive job losses and reverse male migration limiting access to testing | Reduction in high-risk sexual encounters due to lockdown/pandemic |
| 115 | (Medina et al. 2021)  Guatemala | Quantitative, Cohort | 7360 + 16,218 HIV tests | HTS | HTS: 54.7% reduce on HIV testing, 43.7% decrease in clinical samples sent to the DLH for diagnosis of opportunistic infections | COVID-19 restrictions | NA |
| 116 | (Muhula et al. 2021)  Kenya | Quantitative, Cross-sectional | 176 | PP, HTE, LRC | LRC: 56% reduction in uptake of HIV services. 48% decrease in starting ART, 11% did not access health facilities; PP: 24% increase in pre- exposure prophylaxis uptake; HTE: 14% missed medications at the onset of the COVID-19 pandemic | Lack of food, pandemic restrictions, fear of contracting COVID-19 | NA |
| 117 | (Qiao, Yang, et al. 2021)  China | Quantitative, Cross-sectional | 1026 | AA, HTE | AA: 50.4% HIV patients could not get ART refill timely, 43.1% ART adherence compromised. HTE: 60.4% HIV patients could not receive regular follow-up service. | COVID-19 restrictions, clinics closure | NA |
| 118 | (Shi et al. 2021)  China | Quantitative, Cross-sectional | NA | HTS, LRC, HTE | HTS: 49.0% decrease in HIV screening tests, only 63% new diagnoses recorded; LRC: 36.6% drop in positive screening tests; HTE: 10.7% reduction in CD4 count, 28.6% of newly diagnosed PLWH became lost-to-follow-up. | NA | NA |
| 119 | (Suen, Chan, and Wong 2021)  China | Quantitative, Cross-sectional | 1457 | HTE | HTE: 22.9% moderate-to-high levels of difficulty in accessing HIV services, 33.9% mild difficulty and 43.2% no difficulty. | Psychological impacts of COVID-19 on mental health | NA |
| 120 | (Sun et al. 2020)  China | Quantitative, Cross-sectional | 5084 | AA | AA: 35.1% reported a risk of ART interruption (ATI) during outbreak, 18% at imminent risk of ATI and 2.7% experienced interruption. | Relying on receive ART by post, previous experience of ART interruption, travelling away from HIV care site, living in rural area, lockdown, purchase ART out of pocket, being student, fear of disclosing HIV status during receiving ART from post | Obtaining additional ART from government |
| 121 | (Parikh, Chaudhuri, Syam, Singh, et al. 2022)  India | Quantitative, Cross-sectional | 150 | AA, LRC | AA: Most reported increased commitment to ART, though some were unable to continue treatment; LRC: Decrease in seeking care due to HIV-related stigma; several PLWH expressed satisfaction with care, including home visits by HCPs during the pandemic. | Stigma associated with HIV, financial limitations, lack of privacy, inadequate care, logistical, language, and cultural challenges, lack of ART availability in facilities | Resilience and hope bolstered by support systems, medical delivery, telehealth, and counselling services; communities empowered to show compassion for others |
| 122 | (Salako et al. 2022)  Nigeria | Quantitative, Cross-sectional | 344 | AA | AA: challenges in Adherence to ART | COVID-19 restriction, psychological challenges, no significant relation with lack of food and financial and transport difficulties | Financial support (56%), treatment help (24.4%), food security (9.6%), only 4.1% received assistance from governmental or non-governmental groups |
| 123 | (Matsumoto et al. 2022)  Vietnam | Quantitative, Cross-sectional | 1243 | AA, LRC | AA: 95.5% no discontinuation of ART, 1.1% stopped taking ART; LRC: 77.5% received support for ART and HIV treatment at COVID-19,16.6% did not received support | Run out of medicine | HIV patients accepted temporary hospital transfers due to cultural hierarchy and trust in healthcare professionals, continued medication adherence supported by strong patient-authority connections |
| 124 | (Parikh, Chaudhuri, Syam, and Singh 2022)  India | Qualitative, Descriptive | 19 | HMA, HTS | HMA, HTS: Service delivery decreased due to staff shortages, unwillingness to travel, lack of crisis planning, and PLWH’s fear of contracting COVID-19. | Financial and healthcare provider shortage, fear of getting COVID-19 | NA |
| 125 | (Gutiérrez-Velilla et al. 2022)  Mexico | Quantitative, Cross-sectional | 141 | HTE, AA | HTE: 27.6% Follow-up failure (female 17.9%, male 9.2%), AA: 41% inadequate adherence ART. | Lower educational and socioeconomic level, longer times of transportation to the clinic, being attended by different doctors, detectable viral load, previous dropouts from treatment, inadequate antiretroviral adherence, limited HIV knowledge | NA |
| 126 | (Karjadi et al. 2021)  Indonesia | Quantitative, Cross-sectional | 545 | AA, HMA | AA: 3% stopped taking ART; HMA: 48% decrease in hospital visiting | Fear of getting COVID-19 from hospital | online hospital consultation, get medicine through expeditions |
| 127 | (Sun et al. 2021)  China | Qualitative, Case-study | 64 | AA | AA: decrease in ART adherence | Travel restrictions, Inadequate communication and bureaucratic obstacles when attempting to refill ART; Shortage in personnel at HIV care facilities; Privacy concerns of PLWH, stigma; Insufficient reserve in CDC clinics and hospitals and among drug vendors | Mail and home delivery of ART, help to protect privacy of PLWH, guide to refill ART and alternative sources of ART, mental health counselling |
| 128 | (Adugna, Azanaw, and Sharew Melaku 2021)  Ethiopia | Quantitative, Cross-sectional | 51,990 | HTS, AA | HTS: 32% decrease in provider-initiated counselling and testing from pre-COVID to lockdown; 22.5% increase post-lockdown; AA: 20.2% decrease in ART access during lockdown; 22.5% increase post-lockdown. | Fear of contracting COVID, COVID infections, lockdown, and quarantine measures | NA |
| 129 | (Tolossa et al. 2021)  Ethiopia | Quantitative, Cross-sectional | 361 | AA | AA: 21.3% poor adherence | Infrastructure issues, staff shortages, and home visit restrictions | NA |
| 130 | (Z. Wagner et al. 2021)  Uganda | Quantitative, Cohort | 14,632 | AA | AA: 14% higher risk of running out of medication during lockdown | Travel restrictions, food insecurity, disrupted daily routines | NA |
| 131 | (West et al. 2022)  Uganda | Qualitative, Descriptive | 26 | AA | AA: Indirect impact on ART access early in the pandemic | Fear of judgment, transportation barriers, increased travel costs | NA |
| 132 | (Linnemayr et al. 2021)  Uganda | Mixed-methods, Cross-sectional & Descriptive | 100 | AA, HTE | AA: 14% decrease in ART adherence; THE: 76% decrease in clinic access | Limited transportation, fear of COVID infection | NA |
| 133 | (Muwanguzi et al. 2021)  Uganda | Qualitative, Descriptive | 44 | LRC, HTE | LRC: Some newly initiated ART clients were unable to maintain treatment; HTE: A few participants reported treatment interruptions | Limited transportation, COVID-19 measures (stay-at-home directives), stigma/fear of partner | NA |
| 134 | (Thekkur et al. 2021)  Malawi | Quantitative, Cohort | - | HTS | HTS: 39% decrease in HIV testing from pre-COVID to during COVID | Transportation difficulties, community fear of health facilities | NA |
| 135 | (Palattiyil et al. 2022)  Uganda | Mixed-methods, Cross-sectional & descriptive | 229 | HMA, AA | HMA: 52% without access to community outreach had challenged to receive TB or HIV/AIDS services; AA: Difficulty in getting medications | Public transportation restrictions, expensive transport expenses | Reduction in long lines and hours of waiting, administration of medications via VHTs, prolonged dosing regimens |
| 136 | (Nalubega et al. 2021)  Uganda | Qualitative, grounded theory | 17 | LRC | LRC: reengagement and retention in care negatively impacted | Structural: Stigma from home delivery of ART; clinical: Fear of contracting COVID-19, food insecurity; psychological: Fear of HIV stigma and ART interruption; barriers to keeping HIV status secret from spouses during lockdown | Home delivery of HIV medicine |
| 137 | (G. J. Wagner et al. 2022)  Uganda | Three-arm randomized controlled trial & exploratory qualitative | 280 | AA | AA: 8.9% significant decrease in ART adherent | Restricted public transportation, elevated depressive symptoms, food insecurity | Improving mental health, depression treatment |
| 138 | (Chilot, Woldeamanuel, and Manyazewal 2021)  Ethiopia | Quantitative, Cross-sectional | 212 | HMA, HTE, VS | HTE: 27.4% missed refill visits, VS: 26.4% missed follow-up diagnostic test, HMA: 26% missed counseling services | age ≥ 55, fear of COVID-19, age ≥ 55, transport disruption, high cost of traveling to healthcare facilities, limited access to mask and sanitizer, partial lockdown | NA |
| 139 | (Parmley et al. 2023)  Zambia | Qualitative, Descriptive | 60 | PP, HTS, AA, HTE | PP: All participants reported reduced supply of condoms; HTS: all participants reported HIV testing barriers, also benefit from self-testing was positive impact; HTE: evasion of seeking care for HIV, also benefit from mobile clinics; AA: ART inventory depletion reported. | Fear of contracting COVID-19, lockdowns | Self-testing, mobile clinic, appointment reminders |
| 140 | (Kabami et al. 2023)  Uganda | Quantitative, Cohort | 7071 | LRC, HMA, VS | LRC: decline in ART initiation; HMA: less than 10% missed visits; VS: no significant difference | Lockdown | NA |
| 141 | (Mupambireyi et al. 2024)  Zimbabwe | Qualitative, Descriptive | 20 | HTE | HTE: Limited utilization of HIV care and prevention programs | Fear of contracting infection or putting families at risk. fear of COVID-19 and unintentional disclosure of HIV status, financial concerns, clinic closures, staff shortages, reduced operating times, and limitations on the number of patients seen per day | NA |
| 142 | (Kalua et al. 2022)  Malawi | Quantitative, Cohort | 556,281 | VL, HTS | VL: Viral suppression rates increased slightly during the COVID-19 pandemic, rising from 93% before COVID-19 to 94% during COVID-19; HTS: 40% decline in HIV testing | COVID-19 restrictions | Older age, longer time on ART, being women |
| 143 | (Shimels et al. 2023)  Ethiopia | Quantitative, Cross-sectional | 371 | LRC, AA | LRC: 19% challenges in follow-ups; AA: 13.5% availability of medications, 54% perfect ART adherence | COVID-19 restrictions | Having basic education, being married, attending to a health center, and having sleep disturbance |
| 144 | (Emmanuel, Loy, and Patrickson 2022)  Uganda | Quantitative, Cross-sectional | 9952 | AA | AA: 14.7% had inadequate ART utilization | Being obese | Community-focused approaches to ensure continuity of HIV clinic services, phone follow-up for missed appointments during the pandemic |
| 145 | (Lakoh et al. 2023)  Sierra Leone | Quantitative, Cross-sectional | 8538 | LRC, HTS | HTS: 41.2% and 35.7% decline in HIV testing services; LRC: Linkage to care was higher than before pandemic | Fear of COVID-19 exposure, restricted access to services, and disruptions to social life and livelihoods | NA |
| 146 | (Shah et al. 2022)  Congo | Quantitative, Cohort | 36,585 | AA | AA: 52.5% started ART before COVID-19, while 47.5% initiated during the COVID-19 period. | NA | Lockdowns possibly kept patients closer to HIV clinics, altered ART care models enabled wider access to multi-month dispensing, benefitting various age groups. |
| 147 | (Izudi et al. 2022)  Uganda | Quantitative, Quasi-experimental design | 9952 | HTS, VS, LRC, HTE | HTS: 7% decrease in viral load testing coverage; VS: 1% improvement in viral load suppression; LRC: 33.1% of comparison group vs 44.2% of exposed group received viral load testing; HTE: Only 19.1% of participants were retained in care. | COVID-19 lockdown | Nationwide guidelines for sustaining HIV services during restrictions, presidential Emergency Plan for AIDS Relief (PEPFAR), multiple service delivery models for distributing HIV medications |
| 148 | (Mukamba et al. 2022)  Zambia | Qualitative, Descriptive | 25 | AA, HTE, HMA | AA: reduction in ART adherence, also consistency in taking ART; HTE: decrease in seeking HIV care; HMA: decrease in hospital visits for HIV-related care | COVID-19 restrictions, bad attitude of healthcare workers, stigma, drug side effects, food insecurity | Long ART delivery service |
| 149 | (Paine et al. 2023)  Kazakhstan | Quantitative, Cross-sectional | 455 | HTS | HTS: 22% disruptions to HIV testing or care | Discrimination due to stigmatization, victimization of MSM and TSM | NA |
| 150 | (Fauk et al. 2023)  Indonesia | Qualitative, Descriptive | 21 | AA | AA: reduction in ART adherence. | Financial barriers, mandatory COVID vaccine certificate for travelers, lockdown restrictions, lack of ART delivery during lockdown, fear of COVID transmission | NA |
| 151 | (Enane et al. 2021)  Kenya | Quantitative, Cohort | 334 | AA | AA: 3.9% reported problems taking ART; 6.3% missed at least one dose in the past 7 days; 10.9% changed how they took ART during the pandemic. | Lack of food, avoiding interaction with others, pandemic-related challenges | NA |
| 152 | (Siedner et al. 2020)  South Africa | Quantitative, Cohort | 46 523 individuals, 89 476 clinic visits | HMA | HMA: No change in total clinic visits per day during level 5 lockdown; >50% drop in child healthcare visits (from 11.9 to 4.7 visits/day). | Unavailability of clinics due to pandemic | NA |
| 153 | (J. Yang et al. 2022)  China | Mixed method, Cross-sectional & Descriptive | 159 | HTS | HTS: HIV testing significantly increased 29% (from 37% to 66%) because the routine testing services resumed. | COVID-19 lockdown | Resuming HIV testing centers |
| 154 | (El-Krab et al. 2022)  South  Africa | Quantitative, Cross-sectional | 272 | AA, HMA | HMA: 36.4% had appointments canceled by clinics/doctors; 32.7% by service providers due to COVID-19; AA: 43% couldn’t get to the pharmacy; 39% couldn’t receive needed medication. | Clinic closure due to COVID-19, avoiding contracting COVID-19, avoiding social contact | Less education and social and psychological well-being were associated with higher ART adherence |
| 155 | (Luo et al. 2022)  China | Quantitative, Cohort | 2.32 Million individual 4.46 Million orders | HTS | HTS: 51.7% decrease in number of purchasers, 55.3% decrease in orders, and 54.9% decrease in HIV self-testing kits. | COVID-19 restrictions,  decrease in demand of HIV testing because of reduced opportunity to have sex with casual partners due to fear of exposure to COVID-19, severe disruption of express service due to COVID-19 | HIV self-testing through e-commerce platforms |
| 156 | (He et al. 2022)  China | Quantitative, Cross-sectional | 943 | AA, HMA, LRC, HTS | AA: ART interruption was higher in lockdown city (16.4%) vs non-lockdown city (6.9%); HMA: Untimely follow-up appointments were 33.4% vs 14.5%; LRC: Delayed ARV availability was 20.6% vs 8.1%; untimely ARV access via follow-up was 29.1% vs 8.1%; HTS: Delayed CD4 testing follow-up was 33.4% vs 13.9%; never tested during early lockdown was 37% vs 27%; Interruptions in HIV care were linked to lockdown, non-local residence, and mental health disorders. | Healthcare interruptions positively associated with age (18-30, 31-40, 41-50), marital status (married or divorced/widowed), non-local residents and lockdown restrictions | Healthcare interruptions were negatively associated with 1 or less than 1 years or ART, historical diagnosis of non-communicable diseases, and high school education. Availability of ARVs from places other than usual healthcare centers, get a larger volume of medicine each time, actions against discriminatory behavior, more engagement of volunteers/NGOs and psychological counseling centers. |
| 157 | (Enane et al. 2022)  Kenya | Qualitative, Case study | 22 | PP, LRC. HTE | PP: Increased pregnancies linked to family planning and antenatal care barriers; LRC: Clinic care capacity reduced due to funding cuts; HTE: Treatment engagement hindered by disruption of adolescent-friendly services. | Limited funding for adolescents-friendly services, transportation challenges, limited access to care, impaired family planning | NA |
| 158 | (Kalichman et al. 2020)  Georgia | Quantitative, Longitudinal study | 162 | HMA, AA | HMA: 19% canceled clinic/doctor appointments to avoid others; 45% reported cancellations by providers; AA: ART adherence improved during COVID-19 protective measures, though no direct association was found; higher protective actions linked to more nonmedical service cancellations and difficulty accessing medications. | Fear of getting COVID-19, | Health concern |
| 159 | (Gómez-Castro et al. 2022)  Mexico | Quantitative, Cross-sectional | 637 | PP | PP: 26% reduction of PrEP use during lockdown, 6.6% decrease in condom use | Younger age (18-25 years old) | Older than 25 years old, having university education, having less risky sexual behavior (using condom during sex), protective behaviors of getting COVID-19 |
| 160 | (Karaosmanoglu et al. 2022)  Turkey | Quantitative, Cross-sectional | Pre-pandemic 756 Pandemic 315 | HTS, VS | HTS: 58% decrease in new HIV diagnoses; VS: Viral suppression improved during the pandemic; higher proportion of low CD4 counts in pandemic area (36.4% vs 47.9%). | Fear of getting COVID-19 | Changes in sexual behavior during pandemic |
| 161 | (Wang et al. 2022)  Dominican Republic | Quantitative, Cross-sectional | 187 | HTE, HMA, AA | HTE: 34% stated COVID-19 impacted their HIV care and treatment; HMA: 24% missed HIV care visit; AA: 11% stopped taking HIV medicine | COVID-19 financial concern, mental health challenges, partner emotional abuse | NA |
| 162 | (Matambanadzo et al. 2021)  Zimbabwean | Quantitative, Cross-sectional | 6,539 | PP | PP: PrEP initiation rose from 16% in the first 3 months of 2020 (pre-lockdown) to 63% during the remaining 9 months of lockdown phases. | Reduced movement around the country due to transport limitations during pandemic | Reduced condoms use due to decreased client availability (bar and restaurant closures), increased concern about HIV risk |
| 163 | (Mulaudzi et al. 2022)  South Africa | Quantitative, Cross-sectional | 129 | PP | PP: 107 (83%) had a sexual partner, 65 (60.8%) had only one partner, 77 (72%) used condom, 3.7% had group sex activity | Being male (significantly more likely to have one night stand, change partner weekly, and use alcohol) | Being female (significantly were more likely to have one partner) |
| 164 | (K. C. Zhang et al. 2022)  China | Quantitative, Cross-sectional | 595 | HTS | HTS: 9.7% suspension of HIV testing service by providers, 10.6% reduction of HIV testing by providers, 7.1% difficulty in obtaining HIVST kits, 17% reduction in any kind of HIV testing | Cohabiting or being married to a woman, being heterosexual, avoiding crowded places | Before pandemic use of HIV testing |
| 165 | (Kimanga et al. 2023)  Kenya | Quantitative, Longitudinal Observational | 352,322 | LRC, AA, VS | LRC: 16.1% increase in ART initiation during pandemic compared to pre-pandemic; AA: ART non-adherence decreased by 5.7% during pandemic compared to pre-pandemic, VS: no difference | Pandemic restrictions | Success in scaling up more effective HIV interventions, early ART initiation, adoption of more effective dolutegravir (DTG)-based regimen |
| 166 | (Osei et al. 2023)  Ghana | Quantitative, Longitudinal Observational | NA | HTS, LRC | HTS: 40.3% decrease in HIV testing in April 2020; 26.5% decrease by December 2020; LRC: 39.2% increase in ART initiations in April 2020; average 10% monthly decrease from May to September 2020. | Fear of contracting COVID-19, lockdowns, mandatory mask wearing, reduces access to health services, HIV-related and COVID-19-related stigma, reduction of high-risk behaviors, shortage of PPEs, shortage of ARV | NA |
| 167 | (Ensor et al. 2023)  Botswana | Quantitative, Cross-sectional | 65 | PP, HMA, LRC, AA | LRC: 13% unable to access treatment; AA: 18% reported difficulty adhering to ART; HMA: 28% canceled HIV appointments; 20% missed appointments; PP: 24% had trouble accessing condoms; 17% faced issues with regular contraception. | Social distance measures, unavailability of services, inability to access transport, long queues at health services, fear of COVID-19, lack of clinician availability | NA |
| 168 | (Bocage et al. 2023)  Brazil | Quantitative, Cohort | 1810 | HMA, VS, AA | HMA: median number of care visits decreased from 5 to 3, adequate care visits decreased from 77% to 55.1%; VS: no statistically significant changes; AA: the median number of ART pharmacy pickups was 6 pre-pandemic and 5 post-pandemics | Social distancing. fear of exposure to COVID-19, transportation difficulties, clinic closures, staffing shortages | NA |
| 169 | (Andrade et al. 2023)  Brazil | Quantitative, Cohort | NA | HTS | HTS: 22.4% reduction in diagnosis | COVID-19 restrictions | NA |
| 170 | (Zeng et al. 2023)  China | Quantitative, Cross-sectional | 375 | AA | AA: 13.2% had difficulty accessing ART. | COVID-19 restrictions on drug delivery | NA |
| 171 | (K. Zhang et al. 2022)  China | Quantitative, Cohort | 412 | HTS | HTS: increase in facility-based HIV testing and HIV self-testing | COVID-19 prevention measures, not having a job, lower income | Unprotected sex with non-regular partner |
| 172 | (Núñez et al. 2023)  Mexico | Mixed- method, Longitudinal | 2703+13 | HTS | HTS: decrease in HIV diagnosis | Staff shortage, transportation interruption | Telemedicine |
| 173 | (Sukmaningrum et al. 2023)  Indonesia | Qualitative, Descriptive | 22 | AA, HMA | AA: difficulty to maintain consistent adherence during pandemic; HMA: difficulties in accessing HIV care | Changes in clinic hours and medication pick-up times, traveling expenses, fear of being exposed to COVID-19, financial strain, COVID-19 related stigma | NA |
| 174 | (Jaafari et al. 2023)  Iran | Qualitative, Descriptive | 55 | PP, HTS, HTE | PP, HTS: the most affected services; HTE: less affected service | Pandemic restriction, lack of or late diagnosis of HIV, mental problems, organizational problems | Telehealth, dispensing HIV drugs for longer period |
| 175 | (Skovdal et al. 2023)  Zimbabwe | Qualitative, Cohort | 16 | PP | PP: 100% interruption in condom supply | Center shut down, transport restrictions, fear of COVID-19, stock-out, de-prioritized to receive ART | Accessing prioritized health services, knowing the right people |
| 176 | (Joves, Matulac, and Pagcatipunan 2023)  Philippines | Quantitative, Cross-sectional | 116 | AA | AA: difficulty accessing ART due to being out of stock | Being far from treatment hubs, higher education, checkpoints, and border crossings, being from Northern Luzon, unemployed, aged 18 to 25, lacking psychosocial support, aged 26 to 30, facing issues with ARV stocks, and being employed. | NA |
| 177 | (Suryana et al. 2022)  Indonesia | Quantitative, Cross-sectional | 324 | AA | AA: 2.7% decline in ART adherence compared to pre-COVID-19 | Fear of getting COVID-19 | NA |
| 178 | (Stanton et al. 2023)  South Africa | Quantitative, Cohort | 319 | AA | AA: reduced access to ART | Left city and returned to rural areas with family, no access to permanent clinics, refusal of providers to offer medications to non-registered participants, reduced public transportation, clinic closures, financial difficulties | NA |
| 179 | (Mancuso et al. 2023)  Zimbabwe | Mixed-method | 731 | HMA, PP | HMA: decrease in healthcare access; PP: slight negative impact on access to HIV prevention | Adverse mental health, economic issues, increased violence due to COVID-19 | NA |
| 180 | (Jaafari et al. 2022)  Iran | Qualitative, Descriptive | 18 | LRC, AA | LRC: access to healthcare services became adversely affected; AA: reduced access to ART | Misconception of COVID-19, anxiety and psychological effects, fear of seeking healthcare services, limited access to healthcare, socioeconomic status. | NA |
| 181 | (Moyo, Tshivhase, and Mavhandu-Mudzusi 2022)  Zimbabwe | Qualitative, Phenomenological | 10 | LRC | LRC: limited access to HIV services | Misconception of COVID-19, anxiety and psychological effects, fear of seeking healthcare services, limited access to healthcare, socioeconomic status, reduction in public transit | NA |
| 182 | (Piran et al. 2023)  Brazil | Quantitative, Case-control | 136 | AA | AA: 18.7% abandoned ART therapy | Social isolation/distancing, mean age of 22.8 years old, long distance from the service | NA |
| 183 | (Gutiérrez-Velilla et al. 2023)  Mexico | Quantitative, Cross-sectional | 1259 | AA | AA: 7.7% reduction in adherence to ART | Psychological factors are correlated with poor ART adherence. | NA |
| 184 | (Cunha et al. 2022)  Kenya | Quantitative, Cross-sectional | 150 | AA | AA: reduction in ART adherence | Significant barriers: having seronegative partner, being diagnosed 5 to 10 years ago | Having Sero concordant partner, being diagnosed 11 years ago or more |
| 185 | (D. N. Tran et al. 2023)  Nigeria | Qualitative, Descriptive | 10 | HMA, LRC, PP, HTC, VS, HTE | HMA: constraints of healthcare delivery at both the health facility and health-provider levels; LRC, PP, HTC, VS, HTE, AA: interruptions in providing high-quality patient care at each stage of the HIV care continuum. | COVID-19 interruptions of HIV care, | Telemedicine |
| 186 | (Uzim and Lee 2023)  Brazil | Qualitative, Descriptive | 20 | LRC, AA | LRC: clinical interruptions; AA: challenges in medication adherence | Clinical hostilities against young patients, psychological response to the pandemic, stigmas and prejudice, concealment as a self-protection strategy | NA |
| 187 | (Kerzner et al. 2022) (21 PEPFAR-funded countries) | Mixed-methods, Retrospective | 21 countries | HTS, PP | PP: 157.2% increase in PrEP uptake across 21 countries; 100% rise in PrEP initiators; PrEP to need ratio (PnR) increased by 214%. Pre-COVID target met at 91% vs 87% during COVID; HTS: 174% increase in three-month HIV testing among PrEP clients during follow-up. | Prolonged lockdowns and curfews restricted movement and PrEP access, worker strikes, COVID-19 reassignment, and quarantine led to PrEP healthcare worker unavailability, closure of community delivery channels (safe spaces and drop-in centers), school closures limited access to AGYW (Adolescent girls and young women) | Adjustments to preserve access to services: MMD of PrEP, virtual demand creation, community and/or virtual service delivery introduced as best practices |
| 188 | (Santos et al. 2022)  Global study | Quantitative, Cross-sectional | 21795 | PP, AA | AA/PP: COVID-19 worsened economic conditions and hindered HIV prevention and treatment access for sexual and gender minorities; AA: 91.3% of PLWH were on ART; 18.9% reported difficulty accessing or refilling ART, especially among transgender/gender-diverse individuals, those with depression, and disabilities; PP: 11.6% discontinued PrEP during the pandemic; 86.8% reported unchanged or improved access to condoms and lubricants. | Barriers to HIV prevention and sexual health services due to social stigma and structural barriers, younger participants, transgender, or gender-diverse individuals, those with depression or disabilities, sex work history, income reduction, unmet basic needs, and racial/ethnic minorities reported limited access to condoms or lubricants | NA |
| 189 | (Santos et al. 2021)  103 countries | Quantitative, Cross-sectional | 2732 | HTE, LRC | HTE, HTS, AA, PP: Gay men and other MSM faced economic, mental health, and HIV service interruptions, with greater impact among PLWH, racial/ethnic minorities, immigrants, sex workers, and socioeconomically disadvantaged groups. | Severe economic impact on gay men and other MSM due to COVID-19, loss of employment and anticipated income reductions reported by many gay men and other MSM | NA |
| 190 | (Siewe Fodjo et al. 2021)  26 Countries | Quantitative, Cross-sectional | 247 | HMA, LRC, AA | HTE: 27.9% screened positive for anxiety/depression; only 48.6% of PLWH reported HIV care had returned to pre-COVID levels; 43.7% had no HIV follow-up in the past month; AA: 3.6% unable to refill ART; only 44.2% had face-to-face visits with their HIV physician in the past month. | National lockdown that caused social isolation and economic distress | NA |
| 191 | (Kowalska et al. 2020)  20 countries | Quantitative, Cross-sectional | 22 | HMA | HMA: No HIV clinic closures reported; 31.6% of countries had normal operations, 15.4% had reduced hours, and 52.6% reported fully normal activity. | COVID-19 restrictions | NA |
| 192 | (Harris et al. 2022)  11 Sub-Saharan African Countries | Quantitative, Cohort | 1059 health facilities | HTS, LRC, VS, PP | HTS: 3.3% drop in HIV testing and 4.9% drop in HIV-positive results during restrictions; 10.6% increase in testing and 8.8% increase in positives during fewer restrictions; viral load testing increased during restrictions; LRC: 9.8% decrease in new ART initiations during restrictions, followed by a 9.8% increase during fewer restrictions.  VS: No decline in viral load suppression; PP: HIV testing dropped 3.3% from Q2 to Q3 (4.9% drop in positives); increased 10.6% from Q3 to Q4 (8.8% rise in positives). | COVID-19 restrictions, limited access to testing sites, self-imposed reduction in travelling to healthcare centers, lockdowns | Multi month medication dispensing, Virtual adherence consultations |
| 193 | (Rick et al. 2022)  44 countries | Quantitative, Cohort | HTS in 2019: n=2749320; HMA in 2019: n=1903667 | HTS, HMA | HTS: HIV testing dropped globally by 35.4% in 2020 vs 2019, highest in Latin America and the Caribbean (44.6%), lowest in Europe (26.2%); HMA: In-person consultations declined by 7.1% in Africa and 24.3% in Latin America; slight increases observed in Europe and Asia. | Fear of getting COVID-19 | NA |
| 194 | (Restar et al. 2021)  Global study | Quantitative, Cross-sectional | 902 | LRC, HTE | LRC, HTE: 85.8% of PLWH were on treatment, 69.2% had access to an HIV provider, 48.3% had access to treatment, and 44.2% opted for remote prescription refill. | Loss job, less than college education | Older than age group 18-29 years old, college education, higher socioeconomic status |
| 195 | (Rao et al. 2021)  20 countries | Quantitative, Cross-sectional | 10,654 | PP, HTS, LRC, | PP: 56% PrEP interruptions; 10% condom access interruptions; 4% drop in PrEP access per 10-point lockdown increase.  HTS: 38% in-person testing interruptions; 55% HIV self-testing interruptions; 3% and 6% drops in access per 10-point lockdown increase.  LRC: 19.7% could not access HIV providers; 14% accessed providers via telemedicine. | Unable to reach HIV provider in-person or virtually | Telemedicine |
| 196 | (Cordie et al. 2022)  Egypt, Sudan, Tunisia | Quantitative, Cross-sectional | 369 | AA | AA: 93.2% on ART; 31.4% reported increased adherence; 60.5% no change in daily intake; VS: 33.3% virally suppressed; HMA: 59.3% had regular HIV follow-ups. | Financial, medical, and quarantine restrictions. Those greater than 60 years old were more likely to face difficulties in seeking HIV treatment | Longer lasting refills, telephone consultations and web-based self-monitoring |
| 197 | (Wiessing et al. 2023)  Europe, North America, Israel | Qualitative, Descriptive | 13 sites | PP, HTS, AA, HTE | PP: 100% of sites reported decreased or suspended screening; HTS: 53.8% of sites reported decreased HIV case reporting; AA: 38.5% of sites reported reduced ART adherence; HTE: 92.3% of sites reported treatment continuation problems. | COVID-19 lockdown | Telemedicine, longer duration prescription |
| 198 | (Brazier et al. 2022)  Asia-Pacific, CCASAnet, multiple African regions, North America | Quantitative, Cross-sectional | 225 HIV care sites | PP, HTS, LRC, HMA, AA | PP: 6% PrEP and 8% HIV test kit stockouts; HTS: 26% HIV testing suspension; 71% community-based testing suspension; LRC: 10% suspension of new patient enrollment; 6% ART initiation suspension; HMA: 42% postponed non-urgent appointments; AA: 8% ART service suspension; 42% community refill suspension; 81% given extra ART supply; 23% used community pick-up. | Pandemic related restrictions, travel restrictions, | Additional stocks of ART, community-based ART pick-up, expanding same-day ART initiation |
| 199 | (Hung et al. 2022)  10 Asian countries | Quantitative, Cross-sectional | 702 PLWH, 551 KPs, 145 HVPs | HMA, VS, AA, PP | HMA: 35.9% decrease in medical visit; VS: 21.9% reduces HIV RNA viral load testing; AA: 22.3% interruption in ART therapy; PP: 40.9% decrease/complete stop of PrEP | Lockdown, transfer limitations | Telemedicine |
| 200 | (A. Rogers et al. 2022)  Burundi, Cameroon, Democratic Republic of Congo, Republic of Congo, Rwanda | Quantitative, Cross-sectional | 51 clinics | AA, HTS | HTS: In Round 1, 65% reported suspension of community HIV testing, 75% of support groups, and 71% of community tracing; in Round 2, these dropped to 18%, 20%, and 5%, though gaps remained in Rwanda. | Disruptions of HIV service delivery | The study reported the implementation of measures aimed at reducing non-urgent visits to the clinic and providing additional supplies of ART. |

*PP: Prevention and PrEP use; HTS: HIV Testing Services; HMA: HIV Medical Appointments; AA: ART Adherence; LRC: Linkage and Receipt of Care; THE: HIV Treatment Engagement; VS: Viral Suppression

References

Abraham, Susanna Aba Aba, Patience Fakornam Doe, Gifty Osei Berchie, Elizabeth Agyare, Stephen Ayisi Addo, and Dorcas Obiri-Yeboah. 2022. “Explorative–Descriptive Study on the Effects of COVID-19 on Access to Antiretroviral Therapy Services: The Case of a Teaching Hospital in Ghana.” *BMJ Open* 12 (5): e056386. https://doi.org/10.1136/bmjopen-2021-056386.

Abraham, Susanna Aba, Gifty Osei Berchie, Patience Fakornam Doe, Elizabeth Agyare, Stephen Ayisi Addo, and Dorcas Obiri-Yeboah. 2021. “Effects of COVID-19 Pandemic on ART Service Delivery: Perspectives of Healthcare Workers in a Teaching Hospital in Ghana.” *BMC Health Services Research* 21 (1): 1295. https://doi.org/10.1186/s12913-021-07330-2.

Adugna, Asmamaw, Jember Azanaw, and Mequannent Sharew Melaku. 2021. “The Effect of COVID-19 on Routine HIV Care Services from Health Facilities in Northwest Ethiopia.” *HIV/AIDS (Auckland, N.Z.)* 13:1159–68. https://doi.org/10.2147/HIV.S341012.

Ahmed, Ali, Juman Abdulelah Dujaili, Musarat Jabeen, Malik Muhammad Umair, Lay-Hong Chuah, Furqan Khurshid Hashmi, Ahmed Awaisu, and Nathorn Chaiyakunapruk. 2021. “Barriers and Enablers for Adherence to Antiretroviral Therapy Among People Living With HIV/AIDS in the Era of COVID-19: A Qualitative Study From Pakistan.” *Frontiers in Pharmacology* 12:807446. https://doi.org/10.3389/fphar.2021.807446.

Andrade, Lucas Almeida, Thiago De França Amorim, Wandklebson Silva Da Paz, Mariana Do Rosário Souza, Emerson Lucas S. Camargo, Débora Dos Santos Tavares, Shirley Verônica M. A. Lima, et al. 2023. “Reduced HIV/AIDS Diagnosis Rates and Increased AIDS Mortality Due to Late Diagnosis in Brazil during the COVID-19 Pandemic.” *Scientific Reports* 13 (1): 23003. https://doi.org/10.1038/s41598-023-50359-y.

Ballivian, Jamile, Maria L. Alcaide, Diego Cecchini, Deborah L. Jones, John M. Abbamonte, and Isabel Cassetti. 2020. “Impact of COVID-19-Related Stress and Lockdown on Mental Health Among People Living With HIV in Argentina.” *Journal of Acquired Immune Deficiency Syndromes (1999)* 85 (4): 475–82. https://doi.org/10.1097/QAI.0000000000002493.

Barish, Nicole, Shannon Barth, Anne K. Monroe, Alan E. Greenberg, Amanda D. Castel, DC Cohort Executive Committee, Natella Rakhmanina, et al. 2023. “Site Assessment Survey to Assess the Impact of the COVID-19 Pandemic on HIV Clinic Site Services and Strategies for Mitigation in Washington, DC.” *BMC Health Services Research* 23 (1): 1130. https://doi.org/10.1186/s12913-023-10069-7.

Benade, Mariet, Lawrence Long, Sydney Rosen, Gesine Meyer-Rath, Jeanne-Marie Tucker, and Jacqui Miot. 2022. “Reduction in Initiations of HIV Treatment in South Africa during the COVID Pandemic.” *BMC Health Services Research* 22 (1): 428. https://doi.org/10.1186/s12913-022-07714-y.

Bleasdale, Jacob, Lucia A. Leone, Gene D. Morse, Yu Liu, Shelby Taylor, and Sarahmona M. Przybyla. 2022. “Socio-Structural Factors and HIV Care Engagement among People Living with HIV during the COVID-19 Pandemic: A Qualitative Study in the United States.” *Tropical Medicine and Infectious Disease* 7 (10): 259. https://doi.org/10.3390/tropicalmed7100259.

Bocage, Anne E., Lara E. Coelho, Jordan E. Lake, Jesse L. Clark, Thiago S. Torres, Emília M. Jalil, Sandra W. Cardoso, et al. 2023. “The Impact of COVID-19 on HIV Care in Rio de Janeiro, Brazil 2019-2021: Disparities by Age and Gender.” *AIDS and Behavior* 27 (8): 2629–41. https://doi.org/10.1007/s10461-023-03988-3.

Bogart, Laura M., Bisola O. Ojikutu, Keshav Tyagi, David J. Klein, Matt G. Mutchler, Lu Dong, Sean J. Lawrence, Damone R. Thomas, and Sarah Kellman. 2021. “COVID-19 Related Medical Mistrust, Health Impacts, and Potential Vaccine Hesitancy Among Black Americans Living With HIV.” *Journal of Acquired Immune Deficiency Syndromes (1999)* 86 (2): 200–207. https://doi.org/10.1097/QAI.0000000000002570.

Booton, Ross D., Gengfeng Fu, Louis MacGregor, Jianjun Li, Jason J. Ong, Joseph D. Tucker, Katherine Me Turner, Weiming Tang, Peter Vickerman, and Kate M. Mitchell. 2021. “The Impact of Disruptions Due to COVID-19 on HIV Transmission and Control among Men Who Have Sex with Men in China.” *Journal of the International AIDS Society* 24 (4): e25697. https://doi.org/10.1002/jia2.25697.

Brazier, Ellen, Rogers Ajeh, Fernanda Maruri, Beverly Musick, Aimee Freeman, C. William Wester, Man‐Po Lee, et al. 2022. “Service Delivery Challenges in HIV Care during the First Year of the COVID‐19 Pandemic: Results from a Site Assessment Survey across the Global IeDEA Consortium.” *Journal of the International AIDS Society* 25 (12): e26036. https://doi.org/10.1002/jia2.26036.

Brown, Jack Rg, David Reid, Alison R. Howarth, Hamish Mohammed, John Saunders, Caisey V. Pulford, Gwenda Hughes, and Catherine H. Mercer. 2022. “Changes in STI and HIV Testing and Testing Need among Men Who Have Sex with Men during the UK’s COVID-19 Pandemic Response.” *Sexually Transmitted Infections* 99 (4): 226–38. https://doi.org/10.1136/sextrans-2022-055429.

Camp, Christina E., Carrie T. Chan, and Parya Saberi. 2023. “Young Adult Perspectives on Sex, Dating, and PrEP Use During the Pandemic and Improving the Future of PrEP Care.” *AIDS and Behavior* 27 (7): 2430–38. https://doi.org/10.1007/s10461-022-03970-5.

Carbonero-Lechuga, Pablo, Javier Castrodeza-Sanz, Iván Sanz-Muñoz, Pilar Marqués-Sánchez, Jose M. Eiros, Carlos Dueñas-Gutiérrez, and Camino Prada-García. 2023. “Impact of COVID-19 on Adherence to Treatment in Patients with HIV.” *Healthcare* 11 (9): 1299. https://doi.org/10.3390/healthcare11091299.

Celestin, Kemar, Adrien Allorant, Michelle Virgin, Elisma Marinho, Kesner Francois, Jean Guy Honoré, Christina White, et al. 2021. “Short-Term Effects of the COVID-19 Pandemic on HIV Care Utilization, Service Delivery, and Continuity of HIV Antiretroviral Treatment (ART) in Haiti.” *AIDS and Behavior* 25 (5): 1366–72. https://doi.org/10.1007/s10461-021-03218-8.

Chang, Jennifer J., Qiaoling Chen, Jodie Dionne-Odom, Rulin C. Hechter, and Katia J. Bruxvoort. 2022. “Changes in Testing and Diagnoses of Sexually Transmitted Infections and HIV During the COVID-19 Pandemic.” *Sexually Transmitted Diseases* 49 (12): 851–54. https://doi.org/10.1097/OLQ.0000000000001639.

Chilot, Dagmawi, Yimtubezinash Woldeamanuel, and Tsegahun Manyazewal. 2021. “COVID-19 Burden on HIV Patients Attending Antiretroviral Therapy in Addis Ababa, Ethiopia: A Multicenter Cross-Sectional Study.” *Research Square*, July, rs.3.rs-699963. https://doi.org/10.21203/rs.3.rs-699963/v1.

Chow, Eric P. F., Jane S. Hocking, Jason J. Ong, Tina Schmidt, Andrew Buchanan, Elena Rodriguez, Kate Maddaford, Prital Patel, and Christopher K. Fairley. 2020. “Changing the Use of HIV Pre-Exposure Prophylaxis Among Men Who Have Sex With Men During the COVID-19 Pandemic in Melbourne, Australia.” *Open Forum Infectious Diseases* 7 (7): ofaa275. https://doi.org/10.1093/ofid/ofaa275.

Chow, Eric P. F., Jason J. Ong, Basil Donovan, Rosalind Foster, Tiffany R. Phillips, Anna McNulty, and Christopher K. Fairley. 2021. “Comparing HIV Post-Exposure Prophylaxis, Testing, and New Diagnoses in Two Australian Cities with Different Lockdown Measures during the COVID-19 Pandemic.” *International Journal of Environmental Research and Public Health* 18 (20): 10814. https://doi.org/10.3390/ijerph182010814.

Cordie, Ahmed, Mohamed AbdAllah, Eman El Desouky, Sara Gabrallah Mohamed Kheir, Ikbal Kooli, Mohamed Awad Mousnad, Fatima Haj Idris, Heba Abdella, Mohamed Chakroun, and Gamal Esmat. 2022. “The Evolving Challenges Confronting Adults Living with HIV in Three North African Countries during the COVID-19 Crisis: A Survey-Based Study.” *Transactions of the Royal Society of Tropical Medicine and Hygiene* 116 (5): 462–68. https://doi.org/10.1093/trstmh/trab157.

Corneli, Amy, Brian Perry, Jamilah Taylor, Jeremy Beckford, Nneka Molokwu, Susan Reif, Johnny Wilson, et al. 2022. “HIV Prevention During the COVID-19 Pandemic: Sexual Activity and PrEP Use Among Black Same-Gender-Loving Men and Black Cisgender Women.” *AIDS Education and Prevention: Official Publication of the International Society for AIDS Education* 34 (2): 142–57. https://doi.org/10.1521/aeap.2022.34.2.142.

Cunha, Gilmara Holanda Da, Maria Amanda Correia Lima, Larissa Rodrigues Siqueira, Marina Soares Monteiro Fontenele, Ane Kelly Lima Ramalho, and Paulo César De Almeida. 2022. “Lifestyle and Adherence to Antiretrovirals in People with HIV in the COVID-19 Pandemic.” *Revista Brasileira de Enfermagem* 75 (suppl 2): e20210644. https://doi.org/10.1590/0034-7167-2021-0644.

Dark, Tyra, Sitaji Gurung, Mary Dooley, Kit N. Simpson, Seyram A. Butame, and Sylvie Naar. 2022. “Impact of the COVID-19 Pandemic on the Care Continuum of Youth Living with HIV: Qualitative Study of the Scale It Up Program Clinical Sites.” *AIDS and Behavior* 26 (12): 4026–33. https://doi.org/10.1007/s10461-022-03728-z.

Davis, Amelia J., Meredith Greene, Jacob Walker, and Kristine M. Erlandson. 2023. “Perspectives of People Living with HIV Age 50 and over Regarding Barriers and Resources for Care.” *AIDS Care* 35 (4): 581–90. https://doi.org/10.1080/09540121.2022.2162840.

De La Court, Feline, Anders Boyd, Liza Coyer, Mark Van Den Elshout, Henry J. C. De Vries, Amy Matser, Elske Hoornenborg, Maria Prins, and the HIV Transmission Elimination AMsterdam, H‐TEAM) Consortium. 2023. “The Impact of COVID ‐19‐related Restrictions in 2020 on Sexual Healthcare Use, Pre‐exposure Prophylaxis Use, and Sexually Transmitted Infection Incidence among Men Who Have Sex with Men in Amsterdam, the Netherlands.” *HIV Medicine* 24 (2): 212–23. https://doi.org/10.1111/hiv.13374.

Devlin, Samantha A., Amy K. Johnson, Moira C. McNulty, Olivier L. Joseph, André Hall, and Jessica P. Ridgway. 2022. “‘Even If I’m Undetectable, I Just Feel like I Would Die’: A Qualitative Study to Understand the Psychological and Socioeconomic Impacts of the COVID-19 Pandemic on Women Living with HIV (WLWH) in Chicago, IL.” *BMC Women’s Health* 22 (1): 218. https://doi.org/10.1186/s12905-022-01812-z.

Di Ciaccio, Marion, Virginie Villes, David Michels, Stéphane Morel, Rosemary M. Delabre, Daniela Rojas Castro, and Annie Velter. 2022. “Impact of the Early 2020 COVID-19 Crisis and Lockdown on PrEP Use among Men Who Have Sex with Men (MSM) in France.” *Sexually Transmitted Infections* 98 (7): 510–17. https://doi.org/10.1136/sextrans-2021-055189.

Diaz, Monica M., Diego M. Cabrera, Marcela Gil-Zacarias, Valeria Ramirez, Manuel Saavedra, Cesar Cárcamo, Evelyn Hsieh, and Patricia J. Garcia. 2021. “Knowledge and Impact of COVID-19 on Middle-Aged and Older People Living with HIV in Lima, Peru.” *Journal of the International Association of Providers of AIDS Care* 20:23259582211056760. https://doi.org/10.1177/23259582211056760.

Dorward, Jienchi, Thokozani Khubone, Kelly Gate, Hope Ngobese, Yukteshwar Sookrajh, Siyabonga Mkhize, Aslam Jeewa, et al. 2021. “The Impact of the COVID-19 Lockdown on HIV Care in 65 South African Primary Care Clinics: An Interrupted Time Series Analysis.” *The Lancet. HIV* 8 (3): e158–65. https://doi.org/10.1016/S2352-3018(20)30359-3.

Dyer, Jessica, Kate Wilson, Jacinta Badia, Kawango Agot, Jillian Neary, Irene Njuguna, James Kibugi, et al. 2021. “The Psychosocial Effects of the COVID-19 Pandemic on Youth Living with HIV in Western Kenya.” *AIDS and Behavior* 25 (1): 68–72. https://doi.org/10.1007/s10461-020-03005-x.

Eckardt, Paula, Jianli Niu, and Sheila Montalvo. 2021. “Emergency Room ‘Opt-Out’ HIV Testing Pre- and During COVID-19 Pandemic in a Large Community Health System.” *Journal of the International Association of Providers of AIDS Care* 20:23259582211041260. https://doi.org/10.1177/23259582211041260.

Ejima, Keisuke, Yoshiki Koizumi, Nao Yamamoto, Molly Rosenberg, Christina Ludema, Ana I. Bento, Daisuke Yoneoka, Seiichi Ichikawa, Daisuke Mizushima, and Shingo Iwami. 2021. “HIV Testing by Public Health Centers and Municipalities and New HIV Cases During the COVID-19 Pandemic in Japan.” *Journal of Acquired Immune Deficiency Syndromes (1999)* 87 (2): e182–87. https://doi.org/10.1097/QAI.0000000000002660.

El Moussaoui, Majdouline, Nicolas Lambert, Nathalie Maes, Karine Fombellida, Dolores Vaira, Michel Moutschen, and Gilles Darcis. 2021. “Impact of the COVID-19 Pandemic Situation on HIV Care in Liège, Belgium.” *HIV Research & Clinical Practice* 22 (3): 63–70.

El-Krab, Renee, Seth Kalichman, Darshini Govindasamy, Ellen Banas, Moira Kalichman, and Catherine Mathews. 2022. “Subjective Well-Being and COVID-19 Prevention Practices among People Living with HIV in Cape Town, South Africa.” *Global Public Health* 17 (1): 1–12. https://doi.org/10.1080/17441692.2021.2005113.

El-Nahal, Walid G., Nicola M. Shen, Jeanne C. Keruly, Joyce L. Jones, Anthony T. Fojo, Bryan Lau, Yukari C. Manabe, et al. 2022. “Telemedicine and Visit Completion among People with HIV during the Coronavirus Disease 2019 Pandemic Compared with Prepandemic.” *AIDS (London, England)* 36 (3): 355–62. https://doi.org/10.1097/QAD.0000000000003119.

El-Nahal, Walid G., Nicola M. Shen, Jeanne C. Keruly, Joyce L. Jones, Anthony T. Fojo, Yukari C. Manabe, Richard D. Moore, Kelly A. Gebo, Geetanjali Chander, and Catherine R. Lesko. 2022. “Time Between Viral Loads for People With HIV During the COVID-19 Pandemic.” *Journal of Acquired Immune Deficiency Syndromes (1999)* 91 (1): 109–16. https://doi.org/10.1097/QAI.0000000000003026.

Emmanuel, Sendaula, Alupo Anne Loy, and Ayella Patrickson. 2022. “Utilization of ART Services Among People Living with HIV During the COVID-19 Pandemic: A Case of Kampala District.” *The Open AIDS Journal* 16 (1): e187461362208170. https://doi.org/10.2174/18746136-v16-e2208170.

Enane, Leslie A., Edith Apondi, Josephine Aluoch, Giorgos Bakoyannis, Jayne Lewis Kulzer, Zachary Kwena, Rami Kantor, et al. 2021. “Social, Economic, and Health Effects of the COVID-19 Pandemic on Adolescents Retained in or Recently Disengaged from HIV Care in Kenya.” *PloS One* 16 (9): e0257210. https://doi.org/10.1371/journal.pone.0257210.

Enane, Leslie A, Edith Apondi, Claire Liepmann, Judith J Toromo, Mark Omollo, Salim Bakari, Michael Scanlon, Kara Wools-Kaloustian, and Rachel C Vreeman. 2022. “‘We Are Not Going Anywhere’: A Qualitative Study of Kenyan Healthcare Worker Perspectives on Adolescent HIV Care Engagement during the COVID-19 Pandemic.” *BMJ Open* 12 (3): e055948. https://doi.org/10.1136/bmjopen-2021-055948.

Ensor, Samuel, Imogen Mechie, Rebecca Ryan, Aamirah Mussa, Bame Bame, Lefhela Tamuthiba, Neo Moshashane, and Chelsea Morroni. 2023. “Measuring the Impact of COVID-19 Social Distancing Measures on Sexual Health Behaviours and Access to HIV and Sexual and Reproductive Health Services for People Living with HIV in Botswana.” *Frontiers in Global Women’s Health* 4:981478. https://doi.org/10.3389/fgwh.2023.981478.

Fauk, Nelsensius Klau, Hailay Abrha Gesesew, Alfonsa Liquory Seran, and Paul Russell Ward. 2023. “Barriers to Access to Antiretroviral Therapy by People Living with HIV in an Indonesian Remote District during the COVID-19 Pandemic: A Qualitative Study.” *BMC Infectious Diseases* 23 (1): 296. https://doi.org/10.1186/s12879-023-08221-z.

Gabster, Amanda, Jennifer Toller Erausquin, Kristien Michielsen, Philippe Mayaud, Juan Miguel Pascale, Carles Pericas, Michael Marks, et al. 2022. “How Did COVID-19 Measures Impact Sexual Behaviour and Access to HIV/STI Services in Panama? Results from a National Cross-Sectional Online Survey.” *Sexually Transmitted Infections* 98 (5): 332–40. https://doi.org/10.1136/sextrans-2021-054985.

Galaviz, Karla I., N. Sarita Shah, Mariana Gutierrez, Lauren F. Collins, Cecile D. Lahiri, Caitlin A. Moran, Brittany Szabo, et al. 2022. “Patient Experiences with Telemedicine for HIV Care During the First COVID-19 Wave in Atlanta, Georgia.” *AIDS Research and Human Retroviruses* 38 (5): 415–20. https://doi.org/10.1089/AID.2021.0109.

Gaspar, Mark, Cornel Grey, Alex Wells, Mark Hull, Darrell H. S. Tan, Nathan Lachowsky, and Daniel Grace. 2022. “Public Health Morality, Sex, and COVID-19: Sexual Minority Men’s HIV Pre-Exposure Prophylaxis (PrEP) Decision-Making during Ontario’s First COVID-19 Lockdown.” *Critical Public Health* 32 (1): 116–26. https://doi.org/10.1080/09581596.2021.1970720.

Gillespie, D., Z. Couzens, M. de Bruin, D. A. Hughes, A. Jones, R. Ma, A. Williams, et al. 2022. “PrEP Use, Sexual Behaviour, and PrEP Adherence Among Men Who Have Sex with Men Living in Wales Prior to and During the COVID-19 Pandemic.” *AIDS and Behavior* 26 (8): 2746–57. https://doi.org/10.1007/s10461-022-03618-4.

Gómez-Castro, Jose, Diego Cerecero-García, Heleen Vermandere, and Sergio Bautista-Arredondo. 2022. “Changes in Sexual Behavior, PrEP Use, and COVID-19 Experience among Men Who Have Sex with Men in Mexico.” *AIDS and Behavior* 26 (10): 3451–58. https://doi.org/10.1007/s10461-022-03688-4.

Gutiérrez-Velilla, Ester, Vania Barrientos-Casarrubias, María Gómez-Palacio Schjetnan, Lydia E. Perrusquia-Ortiz, Rosa Cruz-Maycott, Claudia Alvarado-de La Barrera, Santiago Ávila-Ríos, and Nancy Patricia Caballero-Suárez. 2023. “Mental Health and Adherence to Antiretroviral Therapy among Mexican People Living with HIV during the COVID-19 Pandemic.” *AIDS Research and Therapy* 20 (1): 34. https://doi.org/10.1186/s12981-023-00532-0.

Gutiérrez-Velilla, Ester, Alicia Piñeirúa-Menéndez, Santiago Ávila-Ríos, and Nancy Patricia Caballero-Suárez. 2022. “Clinical Follow-Up in People Living with HIV During the COVID-19 Pandemic in Mexico.” *AIDS and Behavior* 26 (8): 2798–2812. https://doi.org/10.1007/s10461-022-03626-4.

Gwadz, Marya, Stephanie Campos, Robert Freeman, Charles M. Cleland, Leo Wilton, Dawa Sherpa, Amanda S. Ritchie, et al. 2021. “Black and Latino Persons Living with HIV Evidence Risk and Resilience in the Context of COVID-19: A Mixed-Methods Study of the Early Phase of the Pandemic.” *AIDS and Behavior* 25 (5): 1340–60. https://doi.org/10.1007/s10461-021-03177-0.

Hammoud, Mohamed A., Andrew Grulich, Martin Holt, Lisa Maher, Dean Murphy, Fengyi Jin, Benjamin Bavinton, et al. 2021. “Substantial Decline in Use of HIV Preexposure Prophylaxis Following Introduction of COVID-19 Physical Distancing Restrictions in Australia: Results From a Prospective Observational Study of Gay and Bisexual Men.” *Journal of Acquired Immune Deficiency Syndromes (1999)* 86 (1): 22–30. https://doi.org/10.1097/QAI.0000000000002514.

Harkness, Audrey, Elliott R. Weinstein, Pranusha Atuluru, Daniel Mayo, Ronald Vidal, Carlos E. Rodríguez-Díaz, and Steven A. Safren. 2022. “Latinx Sexual Minority Men’s Access to HIV and Behavioral Health Services in South Florida During COVID-19: A Qualitative Study of Barriers, Facilitators, and Innovations.” *The Journal of the Association of Nurses in AIDS Care: JANAC* 33 (1): 9–21. https://doi.org/10.1097/JNC.0000000000000280.

Harris, Tiffany G., Edward Jaszi, Matthew R. Lamb, Carlos A. Laudari, Maria Lúcia Mendes Furtado, Bonaparte Nijirazana, Ndayizeye Aimé, et al. 2022. “Effects of the Coronavirus Disease 2019 Pandemic on Human Immunodeficiency Virus Services: Findings from 11 Sub-Saharan African Countries.” *Clinical Infectious Diseases: An Official Publication of the Infectious Diseases Society of America* 75 (1): e1046–53. https://doi.org/10.1093/cid/ciab951.

Hazell, George A., Veronica R. Nott, Sara Ayres, Graham Frize, Natalie Kirkhope, Sarah Fidler, and Caroline Foster. 2024. “Impact of SARS-CoV-2 Pandemic on Viral Suppression for Young Adults Living with Perinatally Acquired HIV Infection.” *AIDS Care* 36 (3): 320–25. https://doi.org/10.1080/09540121.2022.2114986.

He, Jiayu, Yingying Ding, Frank Y. Wong, and Na He. 2022. “Health-Care Access and Utilization among HIV-Infected Men Who Have Sex with Men in Two Chinese Municipalities with or without Lockdown amidst Early COVID-19 Pandemic.” *AIDS Care* 34 (11): 1390–99. https://doi.org/10.1080/09540121.2022.2041163.

Hegarty, Benjamin, Amalia Handayani, Sandeep Nanwani, and Ignatius Praptoraharjo. 2021. “Chasing Targets in a Pandemic: The Impact of COVID-19 on HIV Outreach Workers for MSM (Men Who Have Sex with Men) in Jakarta, Indonesia.” *Global Public Health* 16 (11): 1681–95. https://doi.org/10.1080/17441692.2021.1980599.

Hensley, Kathryn S., Carlijn C. E. Jordans, Jeroen J. A. van Kampen, Femke P. N. Mollema, Elisabeth H. Gisolf, Rachida El Moussaoui, Gonneke Hermanides, et al. 2022. “Significant Impact of Coronavirus Disease 2019 (COVID-19) on Human Immunodeficiency Virus (HIV) Care in Hospitals Affecting the First Pillar of the HIV Care Continuum.” *Clinical Infectious Diseases: An Official Publication of the Infectious Diseases Society of America* 74 (3): 521–24. https://doi.org/10.1093/cid/ciab445.

Hentges, Maike, Anna E. Kågesten, Gunnar Brandén, Kyriaki Kosidou, Kristien Michielsen, Anna Mia Ekström, and Elin C. Larsson. 2024. “Effects of COVID-19 Measures on Access to HIV/STI Testing and Condoms among Adults in Sweden: A Cross-Sectional Online Survey.” *Scandinavian Journal of Public Health* 52 (3): 299–308. https://doi.org/10.1177/14034948231217020.

Hill, Brandon J., Brie Anderson, and Li Lock. 2021. “COVID-19 Pandemic, Pre-Exposure Prophylaxis (PrEP) Care, and HIV/STI Testing Among Patients Receiving Care in Three HIV Epidemic Priority States.” *AIDS and Behavior* 25 (5): 1361–65. https://doi.org/10.1007/s10461-021-03195-y.

Hong, Chenglin, Keith J. Horvath, Rob Stephenson, Kimberly M. Nelson, Andrew E. Petroll, Jennifer L. Walsh, and Steven A. John. 2022. “PrEP Use and Persistence Among Young Sexual Minority Men 17-24 Years Old During the COVID-19 Pandemic.” *AIDS and Behavior* 26 (3): 631–38. https://doi.org/10.1007/s10461-021-03423-5.

Hong, Chenglin, David Huh, Rebecca Schnall, Robert Garofalo, Lisa M. Kuhns, Josh Bruce, D. Scott Batey, et al. 2023. “Changes in High-Risk Sexual Behavior, HIV and Other STI Testing, and PrEP Use during the COVID-19 Pandemic in a Longitudinal Cohort of Adolescent Men Who Have Sex with Men 13 to 18 Years Old in the United States.” *AIDS and Behavior* 27 (4): 1133–39. https://doi.org/10.1007/s10461-022-03850-y.

Hongsermeier-Graves, Natasha, Rohan Khazanchi, Jasmine R. Marcelin, and Nada Fadul. 2022. “Structural Vulnerability among Patients with HIV and SARS-CoV-2 Co-Infection: Descriptive Case Series from the U.S. Midwest.” *AIDS Care* 34 (11): 1372–77. https://doi.org/10.1080/09540121.2021.1981224.

Hou, Yushan, Chang Cai, Houlin Tang, Yichen Jin, Fangfang Chen, Dandan Niu, and Fan Lv. 2023. “Sexual Behavior and Perceived Loneliness in Elderly People Living with HIV in China during the COVID-19 Pandemic.” *International Journal of Environmental Research and Public Health* 20 (3): 2714. https://doi.org/10.3390/ijerph20032714.

Howarth, Alison R., John Saunders, David Reid, Isabelle Kelly, Sonali Wayal, Peter Weatherburn, Gwenda Hughes, and Catherine H. Mercer. 2022. “‘Stay at Home …’: Exploring the Impact of the COVID-19 Public Health Response on Sexual Behaviour and Health Service Use among Men Who Have Sex with Men: Findings from a Large Online Survey in the UK.” *Sexually Transmitted Infections* 98 (5): 346–52. https://doi.org/10.1136/sextrans-2021-055039.

Htun Nyunt, Oo, Nanda Myo Aung Wan, Pyae Soan, Oussama Tawil, Myo Kyaw Lwin, May Thu Aung Hsan, Khin Mar Win, and Fabio Mesquita. 2021. “How Myanmar Is Working to Maintain Essential Services for People Living With HIV and Key Populations During the Covid-19 Pandemic.” *Journal of the International Association of Providers of AIDS Care* 20:23259582211017742. https://doi.org/10.1177/23259582211017742.

Hung, Chien-Ching, Sumita Banerjee, Ishwar Gilada, Kimberly Green, Yoji Inoue, Adeeba Kamarulzaman, Kate Leyritana, et al. 2022. “Impact of COVID-19 on the HIV Care Continuum in Asia: Insights from People Living with HIV, Key Populations, and HIV Healthcare Providers.” Edited by Fahad Jibran. *PLOS ONE* 17 (7): e0270831. https://doi.org/10.1371/journal.pone.0270831.

Izudi, Jonathan, Agnes N. Kiragga, Philip Kalyesubula, Stephen Okoboi, and Barbara Castelnuovo. 2022. “Effect of the COVID-19 Pandemic Restrictions on Outcomes of HIV Care among Adults in Uganda.” *Medicine* 101 (36): e30282. https://doi.org/10.1097/MD.0000000000030282.

Jaafari, Zahra, Sana Eybpoosh, Hamid Sharifi, and Mohammad Karamouzian. 2022. “Exploration of the Impact of Coronavirus Disease 2019 on People Living With HIV in Kerman, Iran: A Qualitative Study.” *Journal of the Association of Nurses in AIDS Care* 33 (4): 386–94. https://doi.org/10.1097/JNC.0000000000000303.

Jaafari, Zahra, Hossein Mirzaei, Yousef Moradi, Naser Nasiri, Soheil Mehmandoost, Mehrdad Khezri, Fatemeh Tavakoli, Samaneh Abbaszadeh, and Hamid Sharifi. 2023. “The Impact of the COVID-19 Pandemic on the Provision of HIV/AIDS-Related Services in Iran: A Qualitative Study.” *BMC Health Services Research* 23 (1): 430. https://doi.org/10.1186/s12913-023-09407-6.

Joseph, Olivier L., André Hall, Samantha A. Devlin, Jared Kerman, Jessica Schmitt, Moira C. McNulty, and Jessica P. Ridgway. 2022. “‘When You Have an Immune Disease like HIV and There Is a Pandemic, You Still Have to Pay Your Bills’: COVID-19-Related Challenges among People Living with HIV and Lessons for Care Delivery.” *AIDS Care* 34 (11): 1405–12. https://doi.org/10.1080/09540121.2022.2067314.

Joves, Philip John M., Melgar O. Matulac, and Rodolfo S. Pagcatipunan. 2023. “Barriers to Antiretroviral Medication Adherence in People Living with HIV (PLHIV) at the Time of the COVID-19 Pandemic in the Philippines.” *Tropical Medicine and Infectious Disease* 8 (10): 461. https://doi.org/10.3390/tropicalmed8100461.

Kabami, Jane, Asiphas Owaraganise, Brian Beesiga, Jaffer Okiring, Elijah Kakande, Yea-Hung Chen, Florence Mwangwa, et al. 2023. “Effect of the COVID-19 Lockdown on the HIV Care Continuum in Southwestern Uganda: A Time Series Analysis.” *PloS One* 18 (8): e0289000. https://doi.org/10.1371/journal.pone.0289000.

Kalichman, Seth C., Lisa A. Eaton, Marcie Berman, Moira O. Kalichman, Harold Katner, Soya S. Sam, and Angela M. Caliendo. 2020. “Intersecting Pandemics: Impact of SARS-CoV-2 (COVID-19) Protective Behaviors on People Living With HIV, Atlanta, Georgia.” *Journal of Acquired Immune Deficiency Syndromes (1999)* 85 (1): 66–72. https://doi.org/10.1097/QAI.0000000000002414.

Kalichman, Seth C., Lisa A. Eaton, Moira O. Kalichman, Soya S. Sam, and Angela M. Caliendo. 2023. “Prepandemic Predictors of Medication Adherence and HIV Viral Load During the First Year of COVID-19.” *JAIDS Journal of Acquired Immune Deficiency Syndromes* 92 (3): 242–49. https://doi.org/10.1097/QAI.0000000000003129.

Kalua, Thokozani, Matthias Egger, Andreas Jahn, Tiwonge Chimpandule, Rose Kolola, and Nanina Anderegg. 2022. “HIV Suppression Was Maintained during the COVID-19 Pandemic in Malawi: A Program-Level Cohort Study.” *Journal of Clinical Epidemiology* 150 (October):116–25. https://doi.org/10.1016/j.jclinepi.2022.06.019.

Kamadjou, Audrey, Anna Decock, Thomas Huleux, Alma Depreux, Emmanuelle Aissi, Laura Landre, Véronique Baclet, et al. 2024. “Impact of the COVID-19 Pandemic on Sexual Behaviour and Welfare of HIV Preexposure Prophylaxis Users: A Mixed-Method Study.” *AIDS Care* 36 (3): 343–50. https://doi.org/10.1080/09540121.2023.2206100.

Karaosmanoglu, Hayat Kumbasar, Birgul Mete, Alper Gunduz, Dilek Yildiz Sevgi, Ozlem Altuntas Aydin, Ilyas Dokmetas, and Fehmi Tabak. 2022. “Changing Characteristics of Patients Living with HIV/AIDS After the COVID-19 Pandemic in Turkey.” *Current HIV Research* 20 (3): 236–41. https://doi.org/10.2174/1570162X20666220303103805.

Karjadi, Teguh Harjono, Suzy Maria, Evy Yunihastuti, Alvina Widhani, Nia Kurniati, and Darma Imran. 2021. “Knowledge, Attitude, Behavior, and Socioeconomic Conditions of People Living with HIV in Indonesia During the COVID-19 Pandemic: A Cross-Sectional Study.” *HIV/AIDS (Auckland, N.Z.)* 13:1045–54. https://doi.org/10.2147/HIV.S333469.

Keane, A., S. O. Regan, L. Quinn, D. Murphy, B. O. Kelly, A. Lynam, F. Lyons, and E. Devitt. 2022. “Evaluation of the Impact of Human Immunodeficiency Virus Pre-Exposure Prophylaxis on New Human Immunodeficiency Virus Diagnoses during the COVID-19 Pandemic.” *International Journal of STD & AIDS* 33 (1): 99–102. https://doi.org/10.1177/09564624211054587.

Kerzner, Michael, Anindya K. De, Randy Yee, Ryan Keating, Gaston Djomand, Sharon Stash, Sangeeta Rana, et al. 2022. “Pre-Exposure Prophylaxis (PrEP) Uptake and Service Delivery Adaptations during the First Wave of the COVID-19 Pandemic in 21 PEPFAR-Funded Countries.” *PloS One* 17 (4): e0266280. https://doi.org/10.1371/journal.pone.0266280.

Kimanga, Davies O., Valeria N. B. Makory, Amin S. Hassan, Faith Ngari, Margaret M. Ndisha, Kennedy J. Muthoka, Lydia Odero, Gonza O. Omoro, Appolonia Aoko, and Lucy Ng’ang’a. 2023. “Impact of the COVID-19 Pandemic on Routine HIV Care and Antiretroviral Treatment Outcomes in Kenya: A Nationally Representative Analysis.” *PloS One* 18 (11): e0291479. https://doi.org/10.1371/journal.pone.0291479.

Kowalska, J. D., A. Skrzat-Klapaczyńska, D. Bursa, T. Balayan, J. Begovac, N. Chkhartishvili, D. Gokengin, et al. 2020. “HIV Care in Times of the COVID-19 Crisis - Where Are We Now in Central and Eastern Europe?” *International Journal of Infectious Diseases: IJID: Official Publication of the International Society for Infectious Diseases* 96 (July):311–14. https://doi.org/10.1016/j.ijid.2020.05.013.

Krist, Lizette C., Hanne M. L. Zimmermann, Mart van Dijk, Sarah E. Stutterheim, and Kai J. Jonas. 2022. “PrEP Use in Times of COVID-19 in the Netherlands: Men Who Have Sex With Men (MSM) on PrEP Test Less for HIV and Renal Functioning During a COVID-19 Related Lockdown.” *AIDS and Behavior* 26 (11): 3656–66. https://doi.org/10.1007/s10461-022-03693-7.

Labban, Hatun M, Raghad T. Alhuthil, Majda S Al-Attas, Yahya A. Mahzari, Aljohara A Alzaydi, Osailan Y Mohammed, Adel A. Otaif, Abrar M Barnawi, Sahar A Al-Ansari, and Mohammed Y Ogdi. 2023. “The Impact of COVID-19 Lockdown on HIV Care: A Cross-Sectional Study in a Tertiary Center.” *Bahrain Medical Bulletin* 45 (3): 1685–90.

Lakoh, Sulaiman, Moses M. Bangura, Olukemi Adekanmbi, Umu Barrie, Darlinda F. Jiba, Matilda N. Kamara, Daniel Sesay, et al. 2023. “Impact of COVID-19 on the Utilization of HIV Testing and Linkage Services in Sierra Leone: Experience from Three Public Health Facilities in Freetown.” *AIDS and Behavior*, August. https://doi.org/10.1007/s10461-023-04149-2.

Lee, Dooyeon, Eric P. F. Chow, Ivette Aguirre, Christopher K. Fairley, and Jason J. Ong. 2021. “Access to HIV Antiretroviral Therapy among People Living with HIV in Melbourne during the COVID-19 Pandemic.” *International Journal of Environmental Research and Public Health* 18 (23): 12765. https://doi.org/10.3390/ijerph182312765.

Lee, Jeong-A., Yeni Kim, and Jun Yong Choi. 2021. “Impact of the COVID-19 Pandemic on HIV Services in Korea: Results from a Cross-Sectional Online Survey.” *Infection & Chemotherapy* 53 (4): 741–52. https://doi.org/10.3947/ic.2021.0112.

Lesko, Catherine R., Jeanne C. Keruly, Richard D. Moore, Nicola M. Shen, Jarratt D. Pytell, Bryan Lau, Anthony T. Fojo, et al. 2022. “COVID-19 and the HIV Continuum in People Living with HIV Enrolled in Collaborating Consortium of Cohorts Producing NIDA Opportunities (C3PNO) Cohorts.” *Drug and Alcohol Dependence* 241 (December):109355. https://doi.org/10.1016/j.drugalcdep.2022.109355.

Levy, Itzchak, Shahar Michael, Liraz Olmer, Ruth Gofen, Oleg Davidson, Roy Zucker, and Gal Wagner-Kolasko. 2022. “The Impact of COVID-19 Lockdown on Men Having Sex with Men (MSM).” *AIDS Care* 34 (11): 1400–1404. https://doi.org/10.1080/09540121.2022.2049197.

Linnemayr, Sebastian, Larissa Jennings Mayo-Wilson, Uzaib Saya, Zachary Wagner, Sarah MacCarthy, Stewart Walukaga, Susan Nakubulwa, and Yvonne Karamagi. 2021. “HIV Care Experiences During the COVID-19 Pandemic: Mixed-Methods Telephone Interviews with Clinic-Enrolled HIV-Infected Adults in Uganda.” *AIDS and Behavior* 25 (1): 28–39. https://doi.org/10.1007/s10461-020-03032-8.

Liu, Wang-Da, Hsiu-Yin Wang, Sih-Cheng Du, and Chien-Ching Hung. 2022. “Impact of the Initial Wave of COVID-19 Pandemic in Taiwan on Local HIV Services: Results from a Cross-Sectional Online Survey.” *Journal of Microbiology, Immunology, and Infection = Wei Mian Yu Gan Ran Za Zhi* 55 (6 Pt 2): 1135–43. https://doi.org/10.1016/j.jmii.2022.03.002.

Luo, Ganfeng, Lingyun Su, Yuqing Hu, Yiguo Zhou, Yinghui Sun, Anping Feng, Yi-Fan Lin, Xinsheng Wu, and Huachun Zou. 2022. “The Impact of COVID-19 Restrictions on Online Sales of HIV Self-Test Kits and Implications for HIV Prevention: Analysis of Transaction Data from a Leading E-Commerce Platform in China.” *Journal of Acquired Immune Deficiency Syndromes (1999)* 90 (4): 408–17. https://doi.org/10.1097/QAI.0000000000002997.

Lyu, Hang, Yi Zhou, Wencan Dai, Shihan Zhen, Shanzi Huang, Lanlan Zhou, Liqun Huang, and Weiming Tang. 2021. “Solidarity and HIV Testing Willingness During the COVID-19 Epidemic: A Study Among Men Who Have Sex With Men in China.” *Frontiers in Public Health* 9:752965. https://doi.org/10.3389/fpubh.2021.752965.

MacNeill, Justin J., Jacqueline C. Linnes, Randolph D. Hubach, and Natalia M. Rodriguez. 2022. “From Crisis to Crisis: Impacts of the COVID-19 Pandemic on People Living with HIV and HIV/AIDS Service Organizations in Indiana.” *BMC Health Services Research* 22 (1): 622. https://doi.org/10.1186/s12913-022-07998-0.

Mancuso, Noah, Florence Mathebula, Miria Chitukuta, Kudzai V. Matambanadzo, Siyanda Tenza, Krishnaveni Reddy, Lumka Nobula, Doreen Kemigisha, and Marie C. D. Stoner. 2023. “The Impact of COVID-19 on Sexual Behavior, HIV Prevention Interest, General Healthcare Access, and Other HIV Risk Factors among Trial Participants in Malawi, South Africa, Uganda, and Zimbabwe.” *Frontiers in Reproductive Health* 5 (October):1270419. https://doi.org/10.3389/frph.2023.1270419.

Matambanadzo, Primrose, Joanna Busza, Haurovi Mafaune, Lillian Chinyanganya, Fortunate Machingura, Getrude Ncube, Richard Steen, Andrew Phillips, and Frances Mary Cowan. 2021. “‘It Went through the Roof’: An Observation Study Exploring the Rise in PrEP Uptake among Zimbabwean Female Sex Workers in Response to Adaptations during Covid‐19.” *Journal of the International AIDS Society* 24 (S6). https://doi.org/10.1002/jia2.25813.

Matsuda, Elaine Monteiro, Isabela Penteriche de Oliveira, Laura Ballesteros Bao, Fernanda Matsuda Manzoni, Norberto Camilo Campos, Beatriz Brajal Varejão, Maristelly Pereira Leal, Vania Barbosa Nascimento, and Luís Fernando de Macedo Brígido. 2022. “Impact of Covid-19 on People Living with HIV-1: Care and Prevention Indicators at a Local and Nationwide Level, Santo André, Brazil.” *Rev. Saúde Pública* 56. https://doi.org/10.11606/s1518-8787.2022056004314.

Matsumoto, Shoko, Moeko Nagai, Dieu An Dang Luong, Hoai Dung Thi Nguyen, Dung Thi Nguyen, Trang Van Dinh, Giang Van Tran, Junko Tanuma, Thach Ngoc Pham, and Shinichi Oka. 2022. “Evaluation of SARS-CoV-2 Antibodies and the Impact of COVID-19 on the HIV Care Continuum, Economic Security, Risky Health Behaviors, and Mental Health Among HIV-Infected Individuals in Vietnam.” *AIDS and Behavior* 26 (4): 1095–1109. https://doi.org/10.1007/s10461-021-03464-w.

Maurya, Shesh Prakash, Ashutosh Sharma, Ravinder Singh, Hitender Gautam, and Bimal Kumar Das. 2022. “HIV Testing & Diagnosis in 2020 at the Apex Tertiary Referral Hospital of India: Impact of COVID-19 Pandemic.” *AIDS Care* 34 (7): 828–31. https://doi.org/10.1080/09540121.2021.1975631.

Mazzitelli, Maria, Arturo Ciccullo, Gianmaria Baldin, Roberto Cauda, Stefano Rusconi, Andrea Giacomelli, Letizia Oreni, et al. 2021. “Has COVID-19 Changed the Approach to HIV Diagnosis?: A Multicentric Italian Experience.” *Medicine* 100 (41): e27418. https://doi.org/10.1097/MD.0000000000027418.

McCrimmon, Tara, Anne Sundelson, Meruyert Darisheva, Louisa Gilbert, Timothy Hunt, Assel Terlikbayeva, Sholpan Primbetova, and Nabila El-Bassel. 2022. “HIV Care Continuum Services for People Who Inject Drugs in Kazakhstan During COVID-19: A Qualitative Study of Service Provider Perspectives.” *Global Health, Science and Practice* 10 (2): e2100619. https://doi.org/10.9745/GHSP-D-21-00619.

McFall, Allison M., Neia Prata Menezes, Aylur K. Srikrishnan, Sunil S. Solomon, Santhanam Anand, Jiban J. Baishya, Gregory M. Lucas, David D. Celentano, and Shruti H. Mehta. 2022. “Impact of the COVID-19 Pandemic on HIV Prevention and Care Services among Key Populations across 15 Cities in India: A Longitudinal Assessment of Clinic-Based Data.” *Journal of the International AIDS Society* 25 (7): e25960. https://doi.org/10.1002/jia2.25960.

McGinnis, Kathleen A., Melissa Skanderson, Amy C. Justice, Kathleen M. Akgün, Janet P. Tate, Joseph T. King, Christopher T. Rentsch, et al. 2021. “HIV Care Using Differentiated Service Delivery during the COVID-19 Pandemic: A Nationwide Cohort Study in the US Department of Veterans Affairs.” *Journal of the International AIDS Society* 24 Suppl 6 (Suppl 6): e25810. https://doi.org/10.1002/jia2.25810.

McKay, Emily, Emmanuela Ojukwu, Saima Hirani, Tatiana Sotindjo, Ijeoma Okedo-Alex, and Patience Magagula. 2023. “How the COVID-19 Pandemic Influenced HIV Care: Are We Prepared Enough for Future Pandemics? An Assessment of Factors Influencing Access, Utilization, Affordability, and Motivation to Engage with HIV Services amongst African, Caribbean, and Black Women.” *International Journal of Environmental Research and Public Health* 20 (11): 6051. https://doi.org/10.3390/ijerph20116051.

Medina, Narda, Ana Alastruey-Izquierdo, Oscar Bonilla, Brenan Ortíz, Osmar Gamboa, Luis Roberto Salazar, Danicela Mercado, et al. 2021. “Impact of the COVID-19 Pandemic on HIV Care in Guatemala.” *International Journal of Infectious Diseases: IJID: Official Publication of the International Society for Infectious Diseases* 108 (July):422–27. https://doi.org/10.1016/j.ijid.2021.06.011.

Mistler, Colleen B., Christine M. Curley, Aviana O. Rosen, Renee El-Krab, Jeffrey A. Wickersham, Michael M. Copenhaver, Antoine Khati, and Roman Shrestha. 2021. “The Impact of COVID-19 on Access to HIV Prevention Services Among Opioid-Dependent Individuals.” *Journal of Community Health* 46 (5): 960–66. https://doi.org/10.1007/s10900-021-00979-0.

Moitra, Ethan, Jun Tao, Joseph Olsen, Riley D. Shearer, Brian R. Wood, Andrew M. Busch, Andrea LaPlante, Jason V. Baker, and Philip A. Chan. 2022. “Impact of the COVID-19 Pandemic on HIV Testing Rates across Four Geographically Diverse Urban Centres in the United States: An Observational Study.” *Lancet Regional Health. Americas* 7 (March):100159. https://doi.org/10.1016/j.lana.2021.100159.

Moyo, Idah, Livhuwani Tshivhase, and Azwihangwisi Helen Mavhandu-Mudzusi. 2022. “Utilisation of HIV Services by Female Sex Workers in Zimbabwe during the COVID-19 Pandemic: A Descriptive Phenomenological Study.” *African Journal of AIDS Research* 21 (2): 183–93. https://doi.org/10.2989/16085906.2022.2101934.

Muhula, Samuel, Yvonne Opanga, Violet Oramisi, Catherine Ngugi, Caroline Ngunu, Jane Carter, Enock Marita, Joachim Osur, and Peter Memiah. 2021. “Impact of the First Wave of the COVID-19 Pandemic on HIV/AIDS Programming in Kenya: Evidence from Kibera Informal Settlement and COVID-19 Hotspot Counties.” *International Journal of Environmental Research and Public Health* 18 (11): 6009. https://doi.org/10.3390/ijerph18116009.

Mukamba, Njekwa, Anjali Sharma, Chanda Mwamba, Herbert Nyirenda, Marksman Foloko, Kasapo Lumbo, Katerina Christopoulos, et al. 2022. “HIV Care Experiences and Health Priorities during the First Wave of COVID-19: Clients’ Perspectives – a Qualitative Study in Lusaka, Zambia.” *BMC Public Health* 22 (1): 2238. https://doi.org/10.1186/s12889-022-14493-y.

Mulaudzi, Mamakiri, Peace Kiguwa, Campion Zharima, Kennedy Otwombe, Khuthadzo Hlongwane, and Janan J. Dietrich. 2022. “Sexual Risk Behaviors Among Youth in Soweto, South Africa During the COVID-19 National Lockdown.” *Sexual Medicine* 10 (2): 100487. https://doi.org/10.1016/j.esxm.2021.100487.

Mupambireyi, Zivai, Frances M. Cowan, Elizabeth Chappell, Anesu Chimwaza, Ngoni Manika, Catherine J. Wedderburn, Hannah Gannon, et al. 2024. “‘Getting Pregnant during COVID-19 Was a Big Risk Because Getting Help from the Clinic Was Not Easy’: COVID-19 Experiences of Women and Healthcare Providers in Harare, Zimbabwe.” Edited by Keshena Naidoo. *PLOS Global Public Health* 4 (1): e0002317. https://doi.org/10.1371/journal.pgph.0002317.

Muwanguzi, Patience A., Paul Kutyabami, Charles Peter Osingada, Esther M. Nasuuna, Freddy Eric Kitutu, Tom Denis Ngabirano, Joyce Nankumbi, et al. 2021. “Conducting an Ongoing HIV Clinical Trial during the COVID-19 Pandemic in Uganda: A Qualitative Study of Research Team and Participants’ Experiences and Lessons Learnt.” *BMJ Open* 11 (4): e048825. https://doi.org/10.1136/bmjopen-2021-048825.

Nalubega, Sylivia, Joshua Kyenkya, Irene Bagaya, Sylvia Nabukenya, Nelson Ssewankambo, Damalie Nakanjako, and Agnes N. Kiragga. 2021. “COVID-19 May Exacerbate the Clinical, Structural and Psychological Barriers to Retention in Care among Women Living with HIV in Rural and Peri-Urban Settings in Uganda.” *BMC Infectious Diseases* 21 (1): 980. https://doi.org/10.1186/s12879-021-06684-6.

Nguyen, Annie L., Mariam Davtyan, Jeff Taylor, Christopher Christensen, Michael Plankey, Stephen Karpiak, and Brandon Brown. 2021. “Living With HIV During the COVID-19 Pandemic: Impacts for Older Adults in Palm Springs, California.” *AIDS Education and Prevention: Official Publication of the International Society for AIDS Education* 33 (4): 265–75. https://doi.org/10.1521/aeap.2021.33.4.265.

Nguyen Thu, Ha, Anh Nguyen Quynh, Oanh Khuat Hai, Ha Le Thi Thanh, and Huong Nguyen Thanh. 2022. “Impact of the COVID-19 Pandemic on Provision of HIV/AIDS Services for Key Populations.” *The International Journal of Health Planning and Management* 37 (5): 2852–68. https://doi.org/10.1002/hpm.3508.

Nitpolprasert, Chattiya, Tarandeep Anand, Nittaya Phanuphak, Peter Reiss, Jintanat Ananworanich, and Holly Landrum Peay. 2022. “A Qualitative Study of the Impact of Coronavirus Disease (COVID-19) on Psychological and Financial Wellbeing and Engagement in Care among Men Who Have Sex with Men Living with HIV in Thailand.” *HIV Medicine* 23 (3): 227–36. https://doi.org/10.1111/hiv.13190.

Niu, Jianli, Candice Sareli, and Paula A. Eckardt. 2022. “Impact of the COVID-19 Pandemic on an Emergency Department-Based Opt-out HIV Screening Program in a South Florida Hospital: An Interrupted Time Series Analysis, July 2018-March 2021.” *American Journal of Infection Control* 50 (9): 994–98. https://doi.org/10.1016/j.ajic.2022.05.004.

Norwood, Jamison, Asghar Kheshti, Bryan E. Shepherd, Peter F. Rebeiro, Aimalohi Ahonkhai, Sean Kelly, and Celestine Wanjalla. 2022. “The Impact of COVID-19 on the HIV Care Continuum in a Large Urban Southern Clinic.” *AIDS and Behavior* 26 (8): 2825–29. https://doi.org/10.1007/s10461-022-03615-7.

Núñez, Isaac, Ana Amuchastegui, Alejandra Vásquez-Salinas, Steven Díaz, and Yanink Caro-Vega. 2023. “Challenges to the HIV Care Continuum During the COVID-19 Pandemic in Mexico: A Mixed Methods Study.” *AIDS and Behavior*, October. https://doi.org/10.1007/s10461-023-04195-w.

Osei, Eric, Hubert Amu, Gideon Kye-Duodu, Mavis Pearl Kwabla, Evans Danso, Fred N. Binka, and So Yoon Kim. 2023. “Impact of COVID-19 Pandemic on Tuberculosis and HIV Services in Ghana: An Interrupted Time Series Analysis.” *PloS One* 18 (9): e0291808. https://doi.org/10.1371/journal.pone.0291808.

Paine, Emily Allen, Yong Gun Lee, Gaukhar Mergenova, Vitaliy Vinogradov, Caitlin I. Laughney, Alissa Davis, Assel Terlikbayeva, Sholpan Primbetova, Timothy Hunt, and Elwin Wu. 2023. “Compounding Vulnerabilities: Victimization and Discrimination Is Associated with COVID-19 Disruptions to HIV-Related Care among Gay, Bisexual, and Other Men and Transgender and Nonbinary People Who Have Sex with Men in Kazakhstan.” *AIDS Care* 35 (5): 651–57. https://doi.org/10.1080/09540121.2022.2148956.

Palacio-Vieira, Jorge, Sergio Moreno-Fornés, Yesika Díaz, Jordi Aceitón, Andreu Bruguera, Daniel K. Nomah, Josep M. Llibre, et al. 2023. “Who Is Lost to Follow-up in HIV Care? Assessment of Care Retention over Time and the Impact of COVID-19. Longitudinal Analysis of the PISCIS Cohort.” *HIV Medicine* 24 (9): 965–78. https://doi.org/10.1111/hiv.13486.

Palattiyil, George, Peter Kisaakye, Hadijah Mwenyango, Simon Katongole, Francis Mulekya, Dina Sidhva, Harish Nair, and Paul Bukuluki. 2022. “Access to HIV/AIDS or TB Care among Refugees in Kampala, Uganda: Exploring the Enablers and Barriers during the COVID-19 Pandemic.” *Journal of Migration and Health* 5:100098. https://doi.org/10.1016/j.jmh.2022.100098.

Pampati, Sanjana, Kayla Emrick, Aaron J. Siegler, and Jeb Jones. 2021. “Changes in Sexual Behavior, PrEP Adherence, and Access to Sexual Health Services Because of the COVID-19 Pandemic Among a Cohort of PrEP-Using MSM in the South.” *Journal of Acquired Immune Deficiency Syndromes (1999)* 87 (1): 639–43. https://doi.org/10.1097/QAI.0000000000002640.

Pan, Yi-Hua, Daniel K. Nomah, Marcos Montoro-Fernandez, Sergio Moreno-Fornés, Yesika Díaz, Jordi Aceitón, Andreu Bruguera, et al. 2024. “The Impact of the COVID-19 Pandemic on Healthcare Services Utilization among People Living with HIV in Catalonia, Spain: A Population-Based Cohort Study.” *Enfermedades Infecciosas Y Microbiologia Clinica (English Ed.)*, January, S2529-993X(24)00001-7. https://doi.org/10.1016/j.eimce.2023.09.004.

Parikh, Neha, Angela Chaudhuri, Syama B. Syam, and Pratishtha Singh. 2022. “Fostering Resilient Health Systems in India: Providing Care for PLHIV Under the Shadow of COVID-19.” *Frontiers in Public Health* 10:836044. https://doi.org/10.3389/fpubh.2022.836044.

Parikh, Neha, Angela Chaudhuri, Syama B. Syam, Pratishtha Singh, Prachi Pal, and Praneeth Pillala. 2022. “Diseases and Disparities: The Impact of COVID-19 Disruptions on Sexual and Reproductive Health Services Among the HIV Community in India.” *Archives of Sexual Behavior* 51 (1): 315–29. https://doi.org/10.1007/s10508-021-02211-5.

Parmley, Lauren E., Tepa Nkumbula, Lophina Chilukutu, Lazarus Chelu, Chipili Mulemfwe, Brave Hanunka, John Mwale, et al. 2023. “Impacts of COVID-19 on Sexual Risk Behaviors, Safe Injection Practices, and Access to HIV Services among Key Populations in Zambia: Findings from a Rapid Qualitative Formative Assessment.” *PloS One* 18 (8): e0289007. https://doi.org/10.1371/journal.pone.0289007.

Petrova, Mariya, Michael Miller-Perusse, Sabina Hirshfield, Adam Carrico, and Keith Horvath. 2022. “Effect of the COVID-19 Pandemic on Stimulant Use and Antiretroviral Therapy Adherence Among Men Who Have Sex With Men Living With HIV: Qualitative Focus Group Study.” *JMIR Formative Research* 6 (5): e30897. https://doi.org/10.2196/30897.

Piran, Camila Moraes Garollo, Alana Vitória Escritori Cargnin, Bianca Machado Cruz Shibukawa, Natan Nascimento De Oliveira, Marcelo Da Silva, and Marcela Demitto Furtado. 2023. “Antiretroviral Therapy Abandonment among Adolescents and Young People with HIV/AIDS during COVID-19: A Case-Control Study.” *Revista Latino-Americana de Enfermagem* 31 (December):e3947. https://doi.org/10.1590/1518-8345.6497.3948.

Platt, Laura, Fatma M. Shebl, Yiqi Qian, Nicholas Spanos, Cody P. Nolan, Kevin L. Ard, and Ingrid V. Bassett. 2023. “Pre-Exposure Prophylaxis Persistence at a Diverse Sexual Health Clinic: Comparison of the Pre-COVID-19 Era to the COVID-19 Era.” *AIDS and Behavior* 27 (8): 2731–40. https://doi.org/10.1007/s10461-023-03996-3.

Pollard, Rose, Usha Gopinath, Yeruva A. Reddy, Bogam R. Kumar, Parthasarathy Mugundu, Canjeevaram K. Vasudevan, Aylur K. Srikrishnan, et al. 2021. “HIV Service Delivery in the Time of COVID-19: Focus Group Discussions with Key Populations in India.” *Journal of the International AIDS Society* 24 Suppl 6 (Suppl 6): e25800. https://doi.org/10.1002/jia2.25800.

Qiao, Shan, Zhenlong Li, Sharon Weissman, Xiaoming Li, Bankole Olatosi, Christal Davis, and Ali B. Mansaray. 2021. “Disparity in HIV Service Interruption in the Outbreak of COVID-19 in South Carolina.” *AIDS and Behavior* 25 (1): 49–57. https://doi.org/10.1007/s10461-020-03013-x.

Qiao, Shan, Xueying Yang, Shufang Sun, Xiaoming Li, Tianyue Mi, Yuejiao Zhou, and Zhiyong Shen. 2021. “Challenges to HIV Service Delivery and the Impacts on Patient Care during COVID-19: Perspective of HIV Care Providers in Guangxi, China.” *AIDS Care* 33 (5): 559–65. https://doi.org/10.1080/09540121.2020.1849532.

Quiros-Roldan, Eugenia, Ilaria Izzo, Canio Carriero, Melania Degli Antoni, Samuele Storti, Giorgio Tiecco, Giulia Gardini, Emanuele Focà, and Francesco Castelli. 2021. “Decrease in New Diagnosis of HIV/AIDS in the Two Years Period 2019-2020: Impact of COVID-19 Pandemic.” *Journal of Public Health Research* 11 (1): 2256. https://doi.org/10.4081/jphr.2021.2256.

Quiros-Roldan, Eugenia, Paola Magro, Canio Carriero, Annacarla Chiesa, Issa El Hamad, Elena Tratta, Raffaella Fazio, Beatrice Formenti, and Francesco Castelli. 2020. “Consequences of the COVID-19 Pandemic on the Continuum of Care in a Cohort of People Living with HIV Followed in a Single Center of Northern Italy.” *AIDS Research and Therapy* 17 (1): 59. https://doi.org/10.1186/s12981-020-00314-y.

Rao, Amrita, Katherine Rucinski, Brooke A. Jarrett, Benjamin Ackerman, Sara Wallach, Julia Marcus, Tyler Adamson, et al. 2021. “Perceived Interruptions to HIV Prevention and Treatment Services Associated With COVID-19 for Gay, Bisexual, and Other Men Who Have Sex With Men in 20 Countries.” *Journal of Acquired Immune Deficiency Syndromes (1999)* 87 (1): 644–51. https://doi.org/10.1097/QAI.0000000000002620.

Restar, Arjee Javellana, Henri M. Garrison-Desany, Tyler Adamson, Chase Childress, Gregorio Millett, Brooke A. Jarrett, Sean Howell, Jennifer L. Glick, S. Wilson Beckham, and Stefan Baral. 2021. “HIV Treatment Engagement in the Context of COVID-19: An Observational Global Sample of Transgender and Nonbinary People Living with HIV.” *BMC Public Health* 21 (1): 901. https://doi.org/10.1186/s12889-021-10977-5.

Rhodes, Scott D., Lilli Mann-Jackson, Jorge Alonzo, Manuel Garcia, Amanda E. Tanner, Benjamin D. Smart, Danielle N. Horridge, Cornelius N. Van Dam, and Aimee M. Wilkin. 2021. “A Rapid Qualitative Assessment of the Impact of the COVID-19 Pandemic on a Racially/Ethnically Diverse Sample of Gay, Bisexual, and Other Men Who Have Sex with Men Living with HIV in the US South.” *AIDS and Behavior* 25 (1): 58–67. https://doi.org/10.1007/s10461-020-03014-w.

Rick, Fernanda, Bruno Issao Ishigami, François José Figueiroa, Lucas Rafael de Castro Cahete, Renato Chuster H. Humar, Roberto de Jesus, Marcos A. Costa Junior, Adele S. Benzaken, and Vivian I. Avelino-Silva. 2021. “Impact of COVID-19 on Income, Prevention Attitudes, and Access to Healthcare among Male Clients in a Sexually Transmitted Infections Clinic.” *The Brazilian Journal of Infectious Diseases: An Official Publication of the Brazilian Society of Infectious Diseases* 25 (5): 101617. https://doi.org/10.1016/j.bjid.2021.101617.

Rick, Fernanda, Wilfred Odoke, Jan van den Hombergh, Adele S. Benzaken, and Vivian I. Avelino-Silva. 2022. “Impact of Coronavirus Disease (COVID-19) on HIV Testing and Care Provision across Four Continents.” *HIV Medicine* 23 (2): 169–77. https://doi.org/10.1111/hiv.13180.

Rogers, Ajeh, Ellen Brazier, Anastase Dzudie, Adebola Adedimeji, Marcel Yotebieng, Benjamin Muhoza, Christella Twizere, et al. 2022. “COVID-19 Associated Changes in HIV Service Delivery over Time in Central Africa: Results from Facility Surveys during the First and Second Waves of the Pandemic.” Edited by Brian C. Zanoni. *PLOS ONE* 17 (11): e0275429. https://doi.org/10.1371/journal.pone.0275429.

Rogers, Brooke G., Jun Tao, Spencer C. Darveau, Michaela Maynard, Alexi Almonte, Siena Napoleon, Matthew Murphy, and Philip A. Chan. 2022. “The Impact of COVID-19 on Sexual Behavior and Psychosocial Functioning in a Clinical Sample of Men Who Have Sex with Men Using HIV Pre-Exposure Prophylaxis.” *AIDS and Behavior* 26 (1): 69–75. https://doi.org/10.1007/s10461-021-03334-5.

Rogers, Brooke G., Jun Tao, Michaela Maynard, Christina Chu, Elizabeth Silva, Emily Toma, Katherine Nagel, Siena Napoleon, and Philip A. Chan. 2021. “Characterizing the Impact of COVID-19 on Pre-Exposure Prophylaxis (PrEP) Care.” *AIDS and Behavior* 25 (11): 3754–57. https://doi.org/10.1007/s10461-021-03337-2.

Rosas Cancio-Suárez, Marta, Cecilia Alonso, María Jesús Vivancos, María Jesús Pérez-Elías, María José Cárdenas, Manuel Vélez-Díaz-Pallarés, María Dolores Corbacho, et al. 2023. “Impact of COVID-19 on the Care of Patients with HIV Infection.” *Journal of Clinical Medicine* 12 (12): 3882. https://doi.org/10.3390/jcm12123882.

Rosen, Joseph G., Leanne Zhang, Danielle Pelaez, Jenell S. Coleman, C. To, Lyra Cooper, Praise F. Olatunde, Teagan Toomre, Jennifer L. Glick, and Ju Nyeong Park. 2022. “Provider Perspectives on HIV Pre-Exposure Prophylaxis Service Disruptions and Adaptations During the COVID-19 Pandemic in Baltimore, Maryland: A Qualitative Study.” *AIDS Patient Care and STDs* 36 (8): 313–20. https://doi.org/10.1089/apc.2022.0058.

Salako, A. O., O. O. Odubela, H. O. Ohwodo, B. Opaneye, S. O. Ojuko, N. L. Nwankwo, Q. E. Ejiga, A. N. David, O. C. Ezechi, and B. L. Salako. 2022. “Challenges of Accessing Care in Art Clinic during COVID-19 Lockdown in Lagos.” *Nigerian Journal of Clinical Practice* 25 (1): 49–54. https://doi.org/10.4103/njcp.njcp_391_20.

Sanchez, Travis H., Maria Zlotorzynska, Mona Rai, and Stefan D. Baral. 2020. “Characterizing the Impact of COVID-19 on Men Who Have Sex with Men Across the United States in April, 2020.” *AIDS and Behavior* 24 (7): 2024–32. https://doi.org/10.1007/s10461-020-02894-2.

Santos, Glenn-Milo, Benjamin Ackerman, Amrita Rao, Sara Wallach, George Ayala, Erik Lamontage, Alex Garner, et al. 2021. “Economic, Mental Health, HIV Prevention and HIV Treatment Impacts of COVID-19 and the COVID-19 Response on a Global Sample of Cisgender Gay Men and Other Men Who Have Sex with Men.” *AIDS and Behavior* 25 (2): 311–21. https://doi.org/10.1007/s10461-020-02969-0.

Santos, Glenn-Milo, Chenglin Hong, Natalie Wilson, Jerry John Nutor, Orlando Harris, Alex Garner, Ian Holloway, George Ayala, and Sean Howell. 2022. “Persistent Disparities in COVID-19-Associated Impacts on HIV Prevention and Care among a Global Sample of Sexual and Gender Minority Individuals.” *Global Public Health* 17 (6): 827–42. https://doi.org/10.1080/17441692.2022.2063362.

Schmidt, Mark A., Suzanne B. Salas, Judy L. Donald, Thomas L. Gift, and Guoyu Tao. 2023. “Impact of the Early COVID-19 Pandemic on the Number of HIV Preexposure Prophylaxis Uses and the Proportion of Preexposure Prophylaxis Users Receiving Sexually Transmitted Infection Testing Services.” *Sexually Transmitted Diseases* 50 (5): 304–9. https://doi.org/10.1097/OLQ.0000000000001726.

Shah, Gulzar H., Gina D. Etheredge, Stacy W. Smallwood, Lievain Maluantesa, Kristie C. Waterfield, Osaremhen Ikhile, John Ditekemena, et al. 2022. “HIV Viral Load Suppression before and after COVID-19 in Kinshasa and Haut Katanga, Democratic Republic of the Congo.” *Southern African Journal of HIV Medicine* 23 (1): 1421. https://doi.org/10.4102/sajhivmed.v23i1.1421.

Shi, Lingen, Guangxia Liu, Gengfeng Fu, Nick Zaller, Chongyi Wei, Cui Yang, and Hongjing Yan. 2022. “Psychosocial and Behavioral Correlates with HIV Testing among Men Who Have Sex with Men during the COVID-19 Pandemic in China.” *PloS One* 17 (1): e0262472. https://doi.org/10.1371/journal.pone.0262472.

Shi, Lingen, Weiming Tang, Haiyang Hu, Tao Qiu, Gifty Marley, Xiaoyan Liu, Yuheng Chen, Yunting Chen, and Gengfeng Fu. 2021. “The Impact of COVID-19 Pandemic on HIV Care Continuum in Jiangsu, China.” *BMC Infectious Diseases* 21 (1): 768. https://doi.org/10.1186/s12879-021-06490-0.

Shilo, Guy, and Zohar Mor. 2020. “COVID-19 and the Changes in the Sexual Behavior of Men Who Have Sex With Men: Results of an Online Survey.” *The Journal of Sexual Medicine* 17 (10): 1827–34. https://doi.org/10.1016/j.jsxm.2020.07.085.

Shimels, Tariku, Rodas A. Kassu, Gelila Bogale, Mahteme Bekele, Melsew Getnet, Abrham Getachew, Zewdneh Shewamene, and Mebratu Abraha. 2023. “Adherence to Antiretroviral Medications Among People Living With HIV in the Era of COVID-19 in Central Ethiopia and Perceived Impact of the Pandemic.” *Community Health Equity Research & Policy* 44 (1): 99–107. https://doi.org/10.1177/0272684X221094151.

Siedner, Mark J., John D. Kraemer, Mark J. Meyer, Guy Harling, Thobeka Mngomezulu, Patrick Gabela, Siphephelo Dlamini, et al. 2020. “Access to Primary Healthcare during Lockdown Measures for COVID-19 in Rural South Africa: A Longitudinal Cohort Study.” *medRxiv: The Preprint Server for Health Sciences*, May, 2020.05.15.20103226. https://doi.org/10.1101/2020.05.15.20103226.

Siewe Fodjo, Joseph Nelson, Edlaine Faria de Moura Villela, Stijn Van Hees, Pieter Vanholder, Patrick Reyntiens, and Robert Colebunders. 2021. “Follow-Up Survey of the Impact of COVID-19 on People Living with HIV during the Second Semester of the Pandemic.” *International Journal of Environmental Research and Public Health* 18 (9): 4635. https://doi.org/10.3390/ijerph18094635.

Skovdal, Morten, Tanyaradzwa Maunzagona, Freedom Dzamatira, Phyllis Magoge-Mandizvidza, Rufurwokuda Maswera, Brian Kumbirai Moyo, Constance Nyamukapa, and Simon Gregson. 2023. “‘Condoms Are Hard to Get by’: Access to HIV Prevention Methods during Lockdown of the COVID-19 Epidemic in Eastern Zimbabwe.” *Global Health Action* 16 (1): 2206207. https://doi.org/10.1080/16549716.2023.2206207.

Souleymanov, Rusty, Sana Amjad, Albert McLeod, Michael Payne, Laurie Ringaert, Linda Larcombe, Gayle Restall, and David J. Brennan. 2023. “Impact of the COVID-19 Pandemic on Access to HIV Testing and Condom Use among Two-Spirit, Gay, Bisexual, and Queer (2SGBQ+) Men in Manitoba.” *AIDS Care* 35 (9): 1306–13. https://doi.org/10.1080/09540121.2023.2208324.

Stanton, Amelia M., Abigail P. Blyler, Nzwakie Mosery, Georgia R. Goodman, Rachel Vanderkruik, Kedibone Sithole, C. Andres Bedoya, Jennifer Smit, and Christina Psaros. 2023. “‘I Am Scared, I Do Not Want to Lie’: Exploring the Impacts of COVID-19 on Engagement in Care, Perceived Health, Relationship Dynamics, and Parenting among Postpartum Women with HIV in South Africa.” Preprint. In Review. https://doi.org/10.21203/rs.3.rs-2463315/v1.

Stephenson, Rob, Tanaka M. D. Chavanduka, Matthew T. Rosso, Stephen P. Sullivan, Renée A. Pitter, Alexis S. Hunter, and Erin Rogers. 2021. “Sex in the Time of COVID-19: Results of an Online Survey of Gay, Bisexual and Other Men Who Have Sex with Men’s Experience of Sex and HIV Prevention During the US COVID-19 Epidemic.” *AIDS and Behavior* 25 (1): 40–48. https://doi.org/10.1007/s10461-020-03024-8.

Suen, Yiu Tung, Randolph C. H. Chan, and Eliz Miu Yin Wong. 2021. “An Exploratory Study of Factors Associated with Difficulties in Accessing HIV Services during the COVID-19 Pandemic among Chinese Gay and Bisexual Men in Hong Kong.” *International Journal of Infectious Diseases: IJID: Official Publication of the International Society for Infectious Diseases* 106 (May):358–62. https://doi.org/10.1016/j.ijid.2021.04.005.

Sukmaningrum, Evi, Judith Levy, Made Diah Negara, Devika N/A, Brigitta Dhyah K. Wardhani, Luh Putu Lila Wulandari, and Pande Putu Januraga. 2023. “Lived Experience, Social Support, and Challenges to Health Service Use during the COVID-19 Pandemic among HIV Key Populations in Indonesia.” *Research Square*, September, rs.3.rs-3282353. https://doi.org/10.21203/rs.3.rs-3282353/v1.

Sun, Yinghui, Hui Li, Ganfeng Luo, Xiaojun Meng, Wei Guo, Thomas Fitzpatrick, Yunlong Ao, et al. 2020. “Antiretroviral Treatment Interruption among People Living with HIV during COVID-19 Outbreak in China: A Nationwide Cross-Sectional Study.” *Journal of the International AIDS Society* 23 (11): e25637. https://doi.org/10.1002/jia2.25637.

Sun, Yinghui, Yuewei Zhan, Hui Li, Tanwei Yuan, Yanxiao Gao, Bowen Liang, Anping Feng, et al. 2021. “Stakeholder Efforts to Mitigate Antiretroviral Therapy Interruption among People Living with HIV during the COVID-19 Pandemic in China: A Qualitative Study.” *Journal of the International AIDS Society* 24 (9): e25781. https://doi.org/10.1002/jia2.25781.

Suryana, Ketut, Hamong Suharsono, Agung Wiwiek Indrayani, Luh Nyoman Arya Wisma Ariani, Wayan Wahyu Semara Putra, and Ni Made Dwita Yaniswari. 2022. “Factors Associated with Anti-Retroviral Therapy Adherence among Patients Living with HIV during the COVID-19 Pandemic: A Cross-Sectional Study.” *Frontiers in Psychiatry* 13:824062. https://doi.org/10.3389/fpsyt.2022.824062.

Tamargo, Javier A., Haley R. Martin, Janet Diaz-Martinez, Mary Jo Trepka, Ivan Delgado-Enciso, Angelique Johnson, Raul N. Mandler, Suzanne Siminski, Pamina M. Gorbach, and Marianna K. Baum. 2021. “COVID-19 Testing and the Impact of the Pandemic on the Miami Adult Studies on HIV Cohort.” *Journal of Acquired Immune Deficiency Syndromes (1999)* 87 (4): 1016–23. https://doi.org/10.1097/QAI.0000000000002680.

Thekkur, Pruthu, Hannock Tweya, Sam Phiri, James Mpunga, Thokozani Kalua, Ajay M. V. Kumar, Srinath Satyanarayana, et al. 2021. “Assessing the Impact of COVID-19 on TB and HIV Programme Services in Selected Health Facilities in Lilongwe, Malawi: Operational Research in Real Time.” *Tropical Medicine and Infectious Disease* 6 (2): 81. https://doi.org/10.3390/tropicalmed6020081.

Tolossa, Tadesse, Bizuneh Wakuma, Diriba Mulisa, Merga Besho, Reta Tsegaye, Mekdes Tigistu, Habtamu Kebebe, et al. 2021. “ART Adherence Among People Living with HIV Seeking Services from Public Health Facilities in Western Ethiopia.” *HIV/AIDS (Auckland, N.Z.)* 13:1149–58. https://doi.org/10.2147/HIV.S336647.

Torres, Thiago S., Brenda Hoagland, Daniel R. B. Bezerra, Alex Garner, Emilia M. Jalil, Lara E. Coelho, Marcos Benedetti, Cristina Pimenta, Beatriz Grinsztejn, and Valdilea G. Veloso. 2021. “Impact of COVID-19 Pandemic on Sexual Minority Populations in Brazil: An Analysis of Social/Racial Disparities in Maintaining Social Distancing and a Description of Sexual Behavior.” *AIDS and Behavior* 25 (1): 73–84. https://doi.org/10.1007/s10461-020-02984-1.

Tran, Dan N., Jennifer Ching, Catherine Kafu, Juddy Wachira, Hillary Koros, Maya Venkataramani, Jamil Said, Sonak D. Pastakia, Omar Galárraga, and Becky L. Genberg. 2023. “Interruptions to HIV Care Delivery During Pandemics and Natural Disasters: A Qualitative Study of Challenges and Opportunities From Frontline Healthcare Providers in Western Kenya.” *Journal of the International Association of Providers of AIDS Care (JIAPAC)* 22 (January):232595822311520. https://doi.org/10.1177/23259582231152041.

Tran, Nhu Kieu, Bach Ngoc Vu, and Mary Bachman DeSilva. 2022. “Impacts of the COVID-19 Pandemic on People Living with HIV Who Are Members of Vulnerable Groups in Vietnam.” *AIDS and Behavior* 26 (9): 2855–65. https://doi.org/10.1007/s10461-022-03630-8.

Trepka, Mary Jo, Melissa K. Ward, Robert A. Ladner, Diana M. Sheehan, Tan Li, Cynthia Ibarra, Semiu O. Gbadamosi, Gladys E. Ibañez, and Michele Jean-Gilles. 2022. “HIV Care Access During the COVID-19 Pandemic as Perceived by Racial/Ethnic Minority Groups Served by the Ryan White Program, Miami-Dade County, Florida.” *Journal of the International Association of Providers of AIDS Care* 21:23259582221084536. https://doi.org/10.1177/23259582221084536.

Unigwe, Ikenna F., Robert L. Cook, Jennifer W. Janelle, and Haesuk Park. 2023. “Trends in Recommended Screening and Monitoring Tests for Users of HIV Pre-Exposure Prophylaxis Before and During the COVID-19 Pandemic.” *AJPM Focus* 2 (4): 100134. https://doi.org/10.1016/j.focus.2023.100134.

Uzim, Elochukwu Ernest, and Po-Han Lee. 2023. “Lost to Follow up: The (Non)Psychosocial Barriers to HIV/AIDS Care in Southeast Nigeria.” *AIDS Care*, September, 1–10. https://doi.org/10.1080/09540121.2023.2253507.

Van Beckhoven, Dominique, Ben Serrien, Marion Montourcy, Chris Verhofstede, Dorien Van Den Bossche, Agnes Libois, Deborah De Geyter, et al. 2022. “Impact of COVID-19 on the Belgian HIV Epidemic: Slowdown of HIV Transmission and Testing and Adaptation of Care.” *BMC Infectious Diseases* 22 (1): 901. https://doi.org/10.1186/s12879-022-07879-1.

Vanbaelen, Thibaut, Anke Rotsaert, Bart K. M. Jacobs, Eric Florence, Chris Kenyon, Bea Vuylsteke, Marie Laga, and Reyniers Thijs. 2022. “Why Do HIV Pre-Exposure Prophylaxis Users Discontinue Pre-Exposure Prophylaxis Care? A Mixed Methods Survey in a Pre-Exposure Prophylaxis Clinic in Belgium.” *AIDS Patient Care and STDs* 36 (4): 159–67. https://doi.org/10.1089/apc.2021.0197.

Voisin, Dexter R., Travonne Edwards, Lois M. Takahashi, Silvia Valadez-Tapia, Habiba Shah, Carter Oselett, Nora Bouacha, Andrea Dakin, and Katherine Quinn. 2023. “COVID-19, Retention in HIV Care, and Access to Ancillary Services for Young Black Men Living with HIV in Chicago.” *AIDS and Behavior* 27 (2): 535–44. https://doi.org/10.1007/s10461-022-03789-0.

Wagner, Glenn J., Zachary Wagner, Mahlet Gizaw, Uzaib Saya, Sarah MacCarthy, Barbara Mukasa, Peter Wabukala, and Sebastian Linnemayr. 2022. “Increased Depression during COVID-19 Lockdown Associated with Food Insecurity and Antiretroviral Non-Adherence among People Living with HIV in Uganda.” *AIDS and Behavior* 26 (7): 2182–90. https://doi.org/10.1007/s10461-021-03371-0.

Wagner, Zachary, Barbara Mukasa, Josephine Nakakande, Chad Stecher, Uzaib Saya, and Sebastian Linnemayr. 2021. “Impact of the COVID-19 Pandemic on Use of HIV Care, Antiretroviral Therapy Adherence, and Viral Suppression: An Observational Cohort Study From Uganda.” *Journal of Acquired Immune Deficiency Syndromes (1999)* 88 (5): 448–56. https://doi.org/10.1097/QAI.0000000000002811.

Wang, Yan, Tahilin S. Karver, Clare Barrington, Yeycy Donastorg, Martha Perez, Hoisex Gomez, Wendy Davis, Noya Galai, and Deanna Kerrigan. 2022. “Structural and Psychosocial Impacts of the COVID-19 Pandemic on HIV Care and Treatment Outcomes Among Female Sex Workers in the Dominican Republic.” *Journal of Acquired Immune Deficiency Syndromes (1999)* 89 (5): 481–88. https://doi.org/10.1097/QAI.0000000000002901.

Ward, Melissa K., Stephanie Aleite, Diana M. Sheehan, Tan Li, Semiu O. Gbadamosi, Michèle Jean-Gilles, Robert A. Ladner, and Mary Jo Trepka. 2023. “Self-Reported Nonadherence to Antiretroviral Therapy Among Miami-Dade Ryan White Program Clients During the COVID-19 Pandemic: A Cross-Sectional Study.” *Journal of the Association of Nurses in AIDS Care* 34 (2): 198–206. https://doi.org/10.1097/JNC.0000000000000382.

Weerasuria, Mihiri, Christy Ko, Adam Ehm, Jessica O’Bryan, James McMahon, Ian Woolley, Jennifer Hoy, and Jillian Lau. 2021. “The Impact of the COVID-19 Pandemic on People Living with HIV in Victoria, Australia.” *AIDS Research and Human Retroviruses* 37 (4): 322–28. https://doi.org/10.1089/AID.2021.0007.

Wenlock, Rhys D., Chante Shillingford, John Mear, Duncan Churchill, Jaime H. Vera, and Gillian Dean. 2022. “The Impact of COVID-19 on HIV Testing in the UK’s First Fast-Track HIV City.” *HIV Medicine* 23 (7): 790–96. https://doi.org/10.1111/hiv.13235.

West, N. S., W. Ddaaki, N. Nakyanjo, D. Isabirye, R. Nakubulwa, F. Nalugoda, J. Kagaayi, and C. E. Kennedy. 2022. “‘A Double Stress’: The Mental Health Impacts of the COVID-19 Pandemic Among People Living with HIV in Rakai, Uganda.” *AIDS and Behavior* 26 (1): 261–65. https://doi.org/10.1007/s10461-021-03379-6.

Wiessing, Lucas, V. Sypsa, A. O. Abagiu, A. Arble, N. Berndt, A. Bosch, S. Buskin, et al. 2023. “Impact of COVID-19 & Response Measures on HIV-HCV Prevention Services and Social Determinants in People Who Inject Drugs in 13 Sites with Recent HIV Outbreaks in Europe, North America and Israel.” *AIDS and Behavior* 27 (4): 1140–53. https://doi.org/10.1007/s10461-022-03851-x.

Wion, Rachel K., and Wendy R. Miller. 2021. “The Impact of COVID-19 on HIV Self-Management, Affective Symptoms, and Stress in People Living with HIV in the United States.” *AIDS and Behavior* 25 (9): 3034–44. https://doi.org/10.1007/s10461-021-03335-4.

Wu, Pei-Ying, Hsin-Yun Sun, Wang-Huei Sheng, Szu-Min Hsieh, Yu-Chung Chuang, Yu-Shan Huang, Wang-Da Liu, et al. 2022. “Impact of Coronavirus Disease 2019 on the HIV Testing and Health Care Delivery at a University Hospital in Taiwan, 2020–2021.” *Journal of Microbiology, Immunology and Infection* 55 (6): 1005–12. https://doi.org/10.1016/j.jmii.2022.10.001.

Yang, Jiahui, Mengyao Yi, Han-Zhu Qian, Yuqing Chen, Qidi Zhou, and Xianhong Li. 2022. “Post-Lockdown Rebounding High-Risk Behaviors and HIV Testing Among MSM in China in the Era of the COVID-19 Pandemic.” *Current HIV Research* 20 (4): 287–95. https://doi.org/10.2174/1570162X20666220613120735.

Yang, Xueying, Chengbo Zeng, Cheuk Chi Tam, Shan Qiao, Xiaoming Li, Zhiyong Shen, and Yuejiao Zhou. 2022. “HIV Service Interruptions During the COVID-19 Pandemic in China: The Role of COVID-19 Challenges and Institutional Response from Healthcare Professional’s Perspective.” *AIDS and Behavior* 26 (4): 1270–78. https://doi.org/10.1007/s10461-021-03484-6.

Zapata, Juan Pablo, Madeline Dang, Katherine G. Quinn, Keith J. Horvath, Rob Stephenson, Julia Dickson-Gomez, and Steven A. John. 2022. “COVID-19-Related Disruptions to HIV Testing and Prevention Among Young Sexual Minority Men 17-24 Years Old: A Qualitative Study Using Synchronous Online Focus Groups, April-September 2020.” *Archives of Sexual Behavior* 51 (1): 303–14. https://doi.org/10.1007/s10508-021-02166-7.

Zeng, Jing, Jing Zhang, Jinzhao Xie, Chun Hao, Jinghua Li, Linghua Li, and Jing Gu. 2023. “Association Between the Awareness of Antiretroviral Drugs-Related Services and Drug Accessibility During the COVID-19 Pandemic Among Patients Undergoing Antiretroviral Therapy: A Cross-Sectional Study.” *AIDS and Behavior* 27 (3): 891–900. https://doi.org/10.1007/s10461-022-03825-z.

Zhang, Ke Chun, Yuan Fang, He Cao, Hongbiao Chen, Tian Hu, Ya Qi Chen, Xiaofeng Zhou, and Zixin Wang. 2022. “The Impacts of the COVID-19 Pandemic on HIV Testing Utilization Among Men Who Have Sex With Men in China: Cross-Sectional Online Survey.” *JMIR Public Health and Surveillance* 8 (5): e30070. https://doi.org/10.2196/30070.

Zhang, Kechun, Siyu Chen, Paul Shing-Fong Chan, Yuan Fang, He Cao, Hongbiao Chen, Tian Hu, Yaqi Chen, Xiaofeng Zhou, and Zixin Wang. 2022. “Changes in HIV Testing Utilization Among Chinese Men Who Have Sex With Men During the COVID-19 Pandemic in Shenzhen, China: An Observational Prospective Cohort Study.” *Frontiers in Medicine* 9:842121. https://doi.org/10.3389/fmed.2022.842121.

Zubiago, Julia, Meghan Murphy, Rubeen Guardado, Denise Daudelin, Dustin Patil, and Alysse Wurcel. 2021. “Increased HIV Testing in People Who Use Drugs Hospitalized in the First Wave of the COVID-19 Pandemic.” *Journal of Substance Abuse Treatment* 124 (May):108266. https://doi.org/10.1016/j.jsat.2020.108266.
